# Support vector machine with quantile hyper-spheres for pattern classification

**DOI:** 10.1371/journal.pone.0212361

**Published:** 2019-02-15

**Authors:** Maoxiang Chu, Xiaoping Liu, Rongfen Gong, Jie Zhao

**Affiliations:** 1 School of Electronic and Information Engineering, University of Science and Technology Liaoning, Anshan, Liaoning, China; 2 Department of Electrical Engineering, Lakehead University, Thunder Bay, Ontario, Canada; 3 State Key Laboratory of Robotics and System (HIT), Harbin, Heilongjiang, China; Ulm University, GERMANY

## Abstract

This paper formulates a support vector machine with quantile hyper-spheres (QHSVM) for pattern classification. The idea of QHSVM is to build two quantile hyper-spheres with the same center for positive or negative training samples. Every quantile hyper-sphere is constructed by using pinball loss instead of hinge loss, which makes the new classification model be insensitive to noise, especially the feature noise around the decision boundary. Moreover, the robustness and generalization of QHSVM are strengthened through maximizing the margin between two quantile hyper-spheres, maximizing the inner-class clustering of samples and optimizing the independent quadratic programming for a target class. Besides that, this paper proposes a novel local center-based density estimation method. Based on it, *ρ*-QHSVM with surrounding and clustering samples is given. Under the premise of high accuracy, the execution speed of *ρ*-QHSVM can be adjusted. The experimental results in artificial, benchmark and strip steel surface defects datasets show that the QHSVM model has distinct advantages in accuracy and the *ρ*-QHSVM model is fit for large-scale datasets.

## 1. Introduction

Support vector machine (SVM) [1] proposed by Vapnik and his cooperators has become an excellent tool for machine learning. SVM is a comprehensive technology by integrating the margin maximization principle, kernel skill and dual method. It has perfect statistical theory, which makes SVM be widely applied in many fields [2–4]. In spite of that, great efforts are needed to improve SVM. So, SVMs with different attributes have been proposed, such as least squares SVM (LS-SVM) [5], proximal SVM (PSVM) [6], *v*-SVM [7], fuzzy SVM (FSVM) [8] and pinball loss SVM (Pin-SVM) [9].

In 2007, Jayadeva et al. proposed a twin support vector machine (TWSVM) [10] for pattern classification. TWSVM is derived from generalized eigenvalue proximal SVM (GEPSVM) [11]. GEPSVM and the other multi-surface classifiers [12–13] are used to solve the XOR problems and reduce the computing time of SVM. Similarly, the TWSVM classifier determines two nonparallel separating hyper-planes by solving two quadratic programming problems (QPPs) with smaller size. TWSVM has advantages in classification speed and generalization, which makes TWSVM become a new popular tool for machine learning. Based on TWSVM, some extended TWSVMs have been proposed, such as least squares TWSVM (LS-TSVM) [14], twin bounded SVM (TBSVM) [15], twin parametric-margin SVM (TPMSVM) [16], Laplacian TWSVM (LTWSVM) [17] and weighted TWSVM with local information (WLTSVM) [18].

Support vector data description (SVDD) [19] inspired by support vector classifier is a one-class learning tool. SVDD implements the minimum volume description by building a hyper-sphere for target samples. When negative samples can be used, [19] provided a new SVDD with negative examples (SVDD_neg). SVDD_neg merges negative samples into training dataset to improve the description of hyper-sphere with the minimum volume. Different versions of classifiers have been extended from SVDD because the inner-class of samples can be gathered to the greatest extent. These classifiers include maximal-margin spherical-structured multi-class SVM (MSM-SVM) [20], twin support vector hyper-sphere (TSVH) [21], twin-hypersphere support vector machine (THSVM) [22], maximum margin and minimum volume hyper-spheres machine with pinball loss (Pin-M^3^HM) [23] and least squares twin support vector hyper-sphere (LS-TSVH) [24].

A main challenge for all versions of SVM is to avoid the adverse impact of noise. As mentioned in [9], classification problems may have label noise and feature noise. So, anti-noise versions of SVM have been proposed. [13] proposed L1-norm twin projection support vector machine. In [13], L1-norm is shown to be robust to noise and outliers in data. [25] overcame noise impact on LS-SVM with weight varying. [26] adopted a robust optimization method in SVM to deal with uncertain noise. [27] built a total margin SVM with separating hyperplane which is insensitive to noise. [8] built a fuzzy SVM by applying a fuzzy member into each input sample. Fuzzy SVM can restrain the adverse effect brought by noise. These versions of SVMs have achieved some success in avoiding the adverse impact of noise, but they are not good at dealing with the feature noise around the decision boundary. In 2014, Huang et al. [9] designed a novel Pin-SVM by introducing pinball loss. Pin-SVM uses pinball loss to replace hinge loss, which makes Pin-SVM not only maintain the good property of SVM, but also be less sensitive to noise, especially the feature noise around the decision boundary. As such, the pinball loss has been successively introduced into different versions of SVM in [23], [28] and [29].

In this paper, a novel support vector machine with quantile hyper-spheres (QHSVM) for pattern classification is proposed. It inherits the excellent genes of SVDD_neg, TWSVM and Pin-SVM. QHSVM has the following attributes and advantages.

- QHSVM adopts pinball losses instead of hinge losses. The hinge losses with maximizing the shortest distance between two classes of samples are sensitive to noise. The pinball losses adopt quantile distance to replace the shortest distance. The quantile distance depending on many samples reduces the sensitivity to noise, especially the feature noise around the decision boundary. So, QHSVM improves the anti-noise ability of hyper-spheres by using the pinball losses.
- QHSVM searches for two quantile hyper-spheres with the same center for positive or negative samples. On the premise of using pinball losses, the volume of one quantile hyper-sphere is required to be as small as possible, while that of the other one is required to be as big as possible. Moreover, QHSVM requires the target samples to be close to the same center of two hyper-spheres as much as possible. These attributes ensure that the margin maximization principle and the inner-class clustering maximization of samples are implemented.
- QHSVM has a QPP for positive or negative samples. The QPP makes one class as a target class and makes the other class as a negative class. QHSVM explores the potential information of target samples to the greatest extent. And the negative samples are used to improve the description of hyper-sphere. These attributes improve the generalization of QHSVM.
- In order to meet the classification requirement of high efficiency, a new local center-based density estimation method is proposed. And QHSVM with surrounding and clustering samples (*ρ*-QHSVM) is given. The local center-based density estimation method can appropriately split training samples into surrounding samples and clustering samples. The hyper-spheres of *ρ*-QHSVM will be described by sparse surrounding samples, while the center of hyper-spheres will be clustered by clustering samples.

In [23], Pin-M^3^HM also has the genes of THSVM and Pin-SVM. It seems that our QHSVM is similar to Pin-M^3^HM. In fact, our QHSVM is different from Pin-M^3^HM in the above attributes (b), (c) and (d). Furthermore, our QHSVM formulates two QPPs with the same structures, but Pin-M^3^HM has two QPPs with different structures.

This paper is organized as follows. Section 2 reviews related work. Section 3 proposes the model of QHSVM and the local center-based density estimation method. Section 4 solves the new QHSVM and *ρ*-QHSVM. Section 5 deals with experimental results and Section 6 contains concluding remarks.

## 2. Related work

### 2.1. Support vector machines with hinge loss and pinball loss

For binary classification, the hinge loss is widely used. The hinge loss proposed in [1] brings popular standard SVM classifier. Suppose a training dataset *T*_*r*_ = {(***X***_1_,*y*_1_),(***X***_2_,*y*_2_),⋯,(***X***_*m*_,*y*_*m*_)}, where $X_{i}\in {ℜ}^{d\times 1}$ and *y*_*i*_∈{1,−1}. Standard SVM searches for an optimal separating hyperplane *w*^*T*^*φ*(***x***)+*b* = 0 by convex optimization, where $w\in {ℜ}^{d\times 1}$, b∈ℜ and *φ*(⋅) is a nonlinear feature mapping function. Its corresponding optimization problem can be described as follows:

$$\underset{w,b}{\min}\frac{1}{2}{\left\|w\right\|}^{2}+c\sum_{i=1}^{m}{\left(L_{h}\right)}_{i},$$ (1)

where *c* is a trade-off parameter. The hinge loss (*L*_*h*_)_*i*_ is given by

$${\left(L_{h}\right)}_{i}=\max\{0,u_{i}\},$$ (2)

where *u*_*i*_ = 1−*y*_*i*_(*w*^*T*^*φ*(***X***_*i*_)+*b*). Substituting (2) into (1), the final QPP of SVM can be obtained:

$$\begin{array}{l} \underset{w,b}{\min}\frac{1}{2}{\left\|w\right\|}^{2}+c\sum_{i=1}^{m}\xi_{i}\\ s.t.\;y_{i}(w^{T}\varphi (X_{i})+b)\geq 1-\xi_{i},\\ \;\xi_{i}\geq 0,i=1,2,\cdots ,m. \end{array}$$ (3)

QPP (3) of SVM searches for two support hyper-planes *w*^*T*^*φ*(***x***)+*b* = ±1 by maximizing the shortest distance between two classes of samples. The support hyper-planes belong to boundary hyper-planes. So, SVM is sensitive to noise. In 2014, Huang et al. [9] proposed a Pin-SVM classifier by introducing the pinball loss into standard SVM. Pin-SVM has the good property of standard SVM and is insensitive to noise, especially the feature noise around the decision boundary. The pinball loss in [9] is just like the following:

$${\left(L_{\tau }\right)}_{i}=\left\{ \begin{array}{l} u_{i},\;u_{i}\geq 0,\\ -\tau u_{i},u_{i}<0, \end{array}\right.$$ (4)

where *τ* is an adjusting parameter. Replacing (*L*_*h*_)_*i*_ in (1) with (*L*_*τ*_)_*i*_, the QPP of Pin-SVM can be obtained:

$$\begin{array}{l} \underset{w,b}{\min}\frac{1}{2}{\left\|w\right\|}^{2}+c\sum_{i=1}^{m}\xi_{i}\\ s.t.\;y_{i}(w^{T}\varphi (X_{i})+b)\geq 1-\xi_{i},\\ \;y_{i}(w^{T}\varphi (X_{i})+b)\leq 1+\frac{1}{\tau }\xi_{i},\\ \;i=1,2,\cdots ,m. \end{array}$$ (5)

Pin-SVM is insensitive to noise because the pinball loss is correlated with quantiles [30–31]. The pinball loss in (5) changes the idea of (3) into maximizing the quantile distance. Specially, when *τ*→0, Pin-SVM reduces to SVM. And the decision functions of QPPs (3) and (5) can be determined by using Lagrangian function, Karush-Kuhn-Tucker (KKT) condition and kernel function. Their formulas can be found in [1] and [9].

### 2.2. Twin support vector machine

TWSVM determines two nonparallel hyperplanes by optimization two QPPs, which is different from standard SVM. Each QPP of TWSVM is very much in line with standard SVM. Its size is smaller than single QPP of SVM. So, TWSVM is comparable with SVM in classification accuracy and has higher efficiency. Moreover, TWSVM is excellent at dealing with the dataset with cross planes.

Suppose that $X_{i}^{+}\in {ℜ}^{d\times 1}$ is a sample in class+1 and $X_{j}^{-}\in {ℜ}^{d\times 1}$ is a sample in class-1, where *i* = 1,2,⋯,*m*^+^ and *j* = 1,2,⋯,*m*^−^. Two QPPs of TWSVM can be described as follows:

$$\begin{array}{l} \underset{w^{+},b^{+}}{\min}\frac{1}{2}\sum_{i=1}^{m^{+}}{\left({\left(w^{+}\right)}^{T}\varphi (X_{i}^{+})+b^{+}\right)}^{2}+c_{1}^{+}\sum_{j=1}^{m^{-}}\xi_{j}\\ s.t.\;-\left({\left(w^{+}\right)}^{T}\varphi (X_{j}^{-})+b^{+}\right)\geq 1-\xi_{j},\\ \;\xi_{j}\geq 0,j=1,2,\cdots ,m^{-}, \end{array}$$ (6)

$$\begin{array}{l} \underset{w^{-},b^{-}}{\min}\frac{1}{2}\sum_{j=1}^{m^{-}}{\left({\left(w^{-}\right)}^{T}\varphi (X_{j}^{-})+b^{-}\right)}^{2}+c_{1}^{-}\sum_{i=1}^{m^{+}}\xi_{i}\\ s.t.\;{\left(w^{-}\right)}^{T}\varphi (X_{i}^{+})+b^{-}\geq 1-\xi_{i},\\ \;\xi_{i}\geq 0,i=1,2,\cdots ,m^{+}, \end{array}$$ (7)

where $c_{1}^{+}$ and $c_{1}^{-}$ are trade-off parameters. QPPs (6) and (7) determine two nonparallel support hyper-planes (***w***^+^)^*T*^*φ*(***x***)+*b*^+^ = 0 and (***w***^−^)^*T*^*φ*(***x***)+*b*^−^ = 0. A new sample is assigned to class +1 or -1 depending on the following decision function:

$$f(x)=\arg\;\min\left\{\left|{\left(w^{+}\right)}^{T}\varphi (x)+b^{+}|,|{\left(w^{-}\right)}^{T}\varphi (x)+b^{-}\right|\right\}.$$ (8)

### 2.3. Support vector data description with negative examples

SVDD is an efficient method to solve a one-class data description problem. It builds a hyper-sphere to cover one class of target samples by the description of the minimum volume. The hyper-sphere embodies the inner-class clustering maximization of samples. Based on SVDD, SVDD_neg adds negative samples. When negative samples can be used, they can improve the hyper-sphere description of target samples. QPP of SVDD_neg can be given by

$$\begin{array}{l} \underset{R^{2},C}{\min}R^{2}+c_{1}\sum_{i=1}^{m^{+}}\xi_{i}+c_{2}\sum_{j=1}^{m^{-}}\xi_{j}\\ s.t.\;\|\varphi (X_{i}^{+})-C{\|}^{2}\leq R^{2}+\xi_{i},\xi_{i}\geq 0,\\ \;\|\varphi (X_{j}^{-})-C{\|}^{2}\geq R^{2}-\xi_{j},\xi_{j}\geq 0,\\ \;i=,2,\cdots ,m^{+},j=1,2,\cdots ,m^{-}, \end{array}$$ (9)

where *R* and ***C*** are the radius and center of the hyper-sphere respectively. QPP (9) requires the target samples be inside of the hyper-sphere and the negative samples be outside of the hyper-sphere. On one hand, this requirement ensures that the hyper-sphere describes a closed boundary around the target samples well. On the other hand, it can be used to distinguish the target samples and negative samples. Inspired by SVDD_neg, some classifiers with hyper-sphere have been proposed in [20–24].

## 3. Support vector machine with quantile hyper-spheres

### 3.1. Pinball losses for quantile hyper-spheres

**Fig 1:**
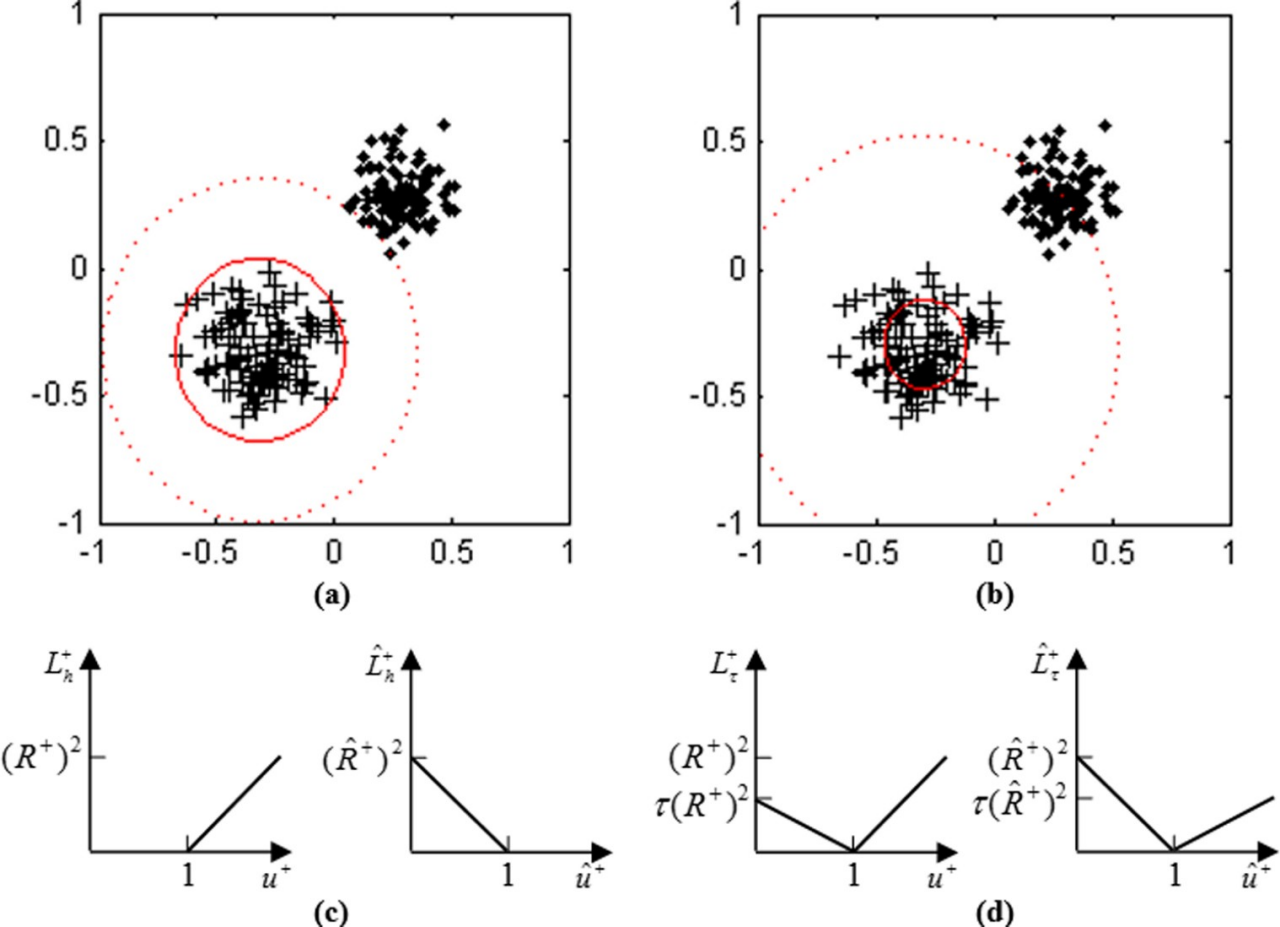
The illustration of boundary hyper-spheres, quantile hyper-spheres, hinge losses and pinball losses. (a) Two boundary hyper-spheres for BHSVM, (b) Two quantile hyper-spheres for QHSVM, (c) Hinge losses for BHSVM and (d) Pinball losses for QHSVM.

The idea of QHSVM is similar to SVDD_neg in building a hyper-sphere. However, it needs to build two hyper-spheres with the same center for target samples, which is different from SVDD_neg. We consider a support vector machine with boundary hyper-spheres (BHSVM). BHSVM has two boundary hyper-spheres with the same center for target samples, which are shown in **Fig 1(A)**. For binary classification, $X_{i}^{+}$ is firstly considered as a target sample. So, $X_{j}^{-}$ is considered as a negative sample. These two boundary hyper-spheres must satisfy the following inequality constraints:

$$\|\varphi (X_{i}^{+})-C^{+}{\|}^{2}\leq {\left(R^{+}\right)}^{2}+\xi_{i}^{+},\xi_{i}^{+}\geq 0,$$ (10)

$$\|\varphi (X_{j}^{-})-C^{+}{\|}^{2}\geq {\left({\hat{R}}^{+}\right)}^{2}-{\hat{\xi }}_{j}^{+},{\hat{\xi }}_{j}^{+}\geq 0,$$ (11)

where *R*^+^ is the radius of the boundary hyper-sphere covering the target samples and ${\hat{R}}^{+}$ is the radius of the other boundary hyper-sphere. ***C***^+^ is the center of the two hyper-spheres. And the negative samples are outside of the hyper-sphere with the radius ${\hat{R}}^{+}$. $\xi_{i}^{+}$ and ${\hat{\xi }}_{j}^{+}$ are the corresponding slack variables. Moreover, BHSVM requires min *R*^+^ and $\max{\hat{R}}^{+}$. So, BHSVM satisfying (10) and (11) maximizes the shortest distance between two classes of samples. Hinge losses are adopted in (10) and (11), which can be given as

$$\left\{ \begin{array}{l} {\left(L_{h}^{+}\right)}_{i}=\max\left\{0,(R^{+}{)}^{2}\left(u_{i}^{+}-1\right)\right\},\\ {\left({\hat{L}}_{h}^{+}\right)}_{i}=\max\left\{0,({\hat{R}}^{+}{)}^{2}\left(1-{\hat{u}}_{i}^{+}\right)\right\}, \end{array}\right.$$ (12)

where

$$\left\{ \begin{array}{l} u_{i}^{+}=\frac{\|\varphi (X_{i}^{+})-C^{+}{\|}^{2}}{{\left(R^{+}\right)}^{2}},\\ {\hat{u}}_{i}^{+}=\frac{\|\varphi (X_{j}^{-})-C^{+}{\|}^{2}}{{\left({\hat{R}}^{+}\right)}^{2}}. \end{array}\right.$$ (13)

The hinge losses (12) are shown in **Fig 1(C)**. It is known that the hinge losses are sensitive to noise [9]. In order to reduce the adverse effect brought by noise, QHSVM is generated by introducing the pinball losses to BHSVM. At this term, QHSVM inherits the ideas of Pin-SVM. The pinball losses for quantile hyper-spheres can be expressed as follows:

$$\begin{array}{l} {\left(L_{\tau }^{+}\right)}_{i}=\left\{ \begin{array}{l} (R^{+}{)}^{2}\left(u_{i}^{+}-1\right),u_{i}^{+}\geq 1,\\ -\tau (R^{+}{)}^{2}\left(u_{i}^{+}-1\right),u_{i}^{+}<1, \end{array}\right.\\ {\left({\hat{L}}_{\tau }^{+}\right)}_{i}=\left\{ \begin{array}{l} ({\hat{R}}^{+}{)}^{2}\left(1-{\hat{u}}_{i}^{+}\right),{\hat{u}}_{i}^{+}\leq 1,\\ -\tau ({\hat{R}}^{+}{)}^{2}\left(1-{\hat{u}}_{i}^{+}\right),{\hat{u}}_{i}^{+}>1. \end{array}\right. \end{array}$$ (14)

The pinball losses (14) are shown in **Fig 1(D)**. If the hinge losses in (10) and (11) are replaced by (14), then two inequality constraints with pinball losses can be obtained:

$$\|\varphi (X_{i}^{+})-C^{+}{\|}^{2}\leq (R^{+}{)}^{2}+\xi_{i}^{+},\|\varphi (X_{i}^{+})-C^{+}{\|}^{2}\geq (R^{+}{)}^{2}-\frac{1}{\tau }\xi_{i}^{+},$$ (15)

$$\|\varphi (X_{j}^{-})-C^{+}{\|}^{2}\geq ({\hat{R}}^{+}{)}^{2}-{\hat{\xi }}_{j}^{+},\|\varphi (X_{j}^{-})-C^{+}{\|}^{2}\leq ({\hat{R}}^{+}{)}^{2}+\frac{1}{\tau }{\hat{\xi }}_{j}^{+}.$$ (16)

Under the constraints of (15) and (16), the hyper-spheres of QHSVM are insensitive to noise because they are quantile hyper-spheres. The quantile hyper-spheres are shown in **Fig 1(B)**. Maximizing the quantile distance instead of maximizing the shortest distance is implemented. Compared with (10), (15) requires that some samples must be distributed outside of the hyper-sphere, which can be controlled with parameter *τ*. That is to say, maximizing the quantile distance of QHSVM depends on a number of samples. So, QHSVM is insensitive to noise, especially the feature noise around the decision boundary. When *τ*→0, (15) becomes (10). For (16), a similar conclusion can be drawn.

For binary classification, the other case is that $X_{j}^{-}$ is a target sample and $X_{i}^{+}$ is a negative sample. Similarly, the corresponding pinball losses can be obtained as

$$\begin{array}{l} {\left(L_{\tau }^{-}\right)}_{j}=\left\{ \begin{array}{l} (R^{-}{)}^{2}\left(u_{j}^{-}-1\right),u_{j}^{-}\geq 1,\\ -\tau (R^{-}{)}^{2}\left(u_{j}^{-}-1\right),u_{j}^{-}<1, \end{array}\right.\\ {\left({\hat{L}}_{\tau }^{-}\right)}_{j}=\left\{ \begin{array}{l} ({\hat{R}}^{-}{)}^{2}\left(1-{\hat{u}}_{j}^{-}\right),{\hat{u}}_{j}^{-}\leq 1,\\ -\tau ({\hat{R}}^{-}{)}^{2}\left(1-{\hat{u}}_{j}^{-}\right),{\hat{u}}_{j}^{-}>1, \end{array}\right. \end{array}$$ (17)

where

$$\left\{ \begin{array}{l} u_{j}^{-}=\frac{\|\varphi (X_{j}^{-})-C^{-}{\|}^{2}}{{\left(R^{‑}\right)}^{2}},\\ {\hat{u}}_{j}^{-}=\frac{\|\varphi (X_{i}^{+})-C^{-}{\|}^{2}}{{\left({\hat{R}}^{‑}\right)}^{2}}. \end{array}\right.$$ (18)

And the inequality constraints with pinball losses can be expressed as:

$$\|\varphi (X_{j}^{-})-C^{-}{\|}^{2}\leq {\left(R^{-}\right)}^{2}+\xi_{j}^{-},\|\varphi (X_{j}^{-})-C^{-}{\|}^{2}\geq {\left(R^{-}\right)}^{2}-\frac{1}{\tau }\xi_{j}^{-},$$ (19)

$$\|\varphi (X_{i}^{+})-C^{-}{\|}^{2}\geq ({\hat{R}}^{-}{)}^{2}-{\hat{\xi }}_{i}^{-},\|\varphi (X_{i}^{+})-C^{-}{\|}^{2}\leq ({\hat{R}}^{-}{)}^{2}+\frac{1}{\tau }{\hat{\xi }}_{i}^{-},$$ (20)

where ***C***^−^ is the center of two quantile hyper-spheres. *R*^−^ and ${\hat{R}}^{-}$ are the radii of the two quantile hyper-spheres respectively. $\xi_{j}^{-}$ and ${\hat{\xi }}_{i}^{-}$ are the corresponding slack variables.

### 3.2. Primal formulation and analysis

For binary classification, consider two datasets $X^{+}=\{X_{i}^{+}|i=1,2,\cdots ,m^{+}\}$ and $X^{-}=\{X_{j}^{-}|j=1,2,\cdots ,m^{-}\}$. Next, we formulate two QPPs with the inequality constraints (15), (16), (19) and (20):

$$\begin{array}{l} \underset{C^{+},(R^{+}{)}^{2},({\hat{R}}^{+}{)}^{2}}{\min}(R^{+}{)}^{2}-({\hat{R}}^{+}{)}^{2}+c_{2}^{+}\sum_{i=1}^{{\tilde{m}}^{+}}\xi_{i}^{+}+c_{3}^{+}\sum_{j=1}^{{\tilde{m}}^{-}}{\hat{\xi }}_{j}^{+}+v^{+}\sum_{l=1}^{{\bar{m}}^{+}}\varsigma_{l}^{+}\\ s.t.\;\|\varphi ({\tilde{X}}_{i}^{+})-C^{+}{\|}^{2}\leq (R^{+}{)}^{2}+\xi_{i}^{+},\|\varphi ({\tilde{X}}_{i}^{+})-C^{+}{\|}^{2}\geq (R^{+}{)}^{2}-\frac{1}{\tau }\xi_{i}^{+},\\ \;\|\varphi ({\tilde{X}}_{j}^{-})-C^{+}{\|}^{2}\geq ({\hat{R}}^{+}{)}^{2}-{\hat{\xi }}_{j}^{+},\|\varphi ({\tilde{X}}_{j}^{-})-C^{+}{\|}^{2}\leq ({\hat{R}}^{+}{)}^{2}+\frac{1}{\tau }{\hat{\xi }}_{j}^{+},\\ \;\|\varphi ({\bar{X}}_{l}^{+})-C^{+}{\|}^{2}=\varsigma_{l}^{+},({\hat{R}}^{+}{)}^{2}-(R^{+}{)}^{2}\geq 0,\\ \;i=1,2,\cdots ,{\tilde{m}}^{+},j=1,2,\cdots ,{\tilde{m}}^{-},l=1,2,\cdots ,{\bar{m}}^{+}, \end{array}$$ (21)

$$\begin{array}{l} \underset{C^{-},(R^{-}{)}^{2},({\hat{R}}^{-}{)}^{2}}{\min}(R^{-}{)}^{2}-({\hat{R}}^{-}{)}^{2}+c_{2}^{-}\sum_{j=1}^{{\tilde{m}}^{-}}\xi_{j}^{-}+c_{3}^{-}\sum_{i=1}^{{\tilde{m}}^{+}}{\hat{\xi }}_{i}^{-}+v^{-}\sum_{l=1}^{{\bar{m}}^{-}}\varsigma_{l}^{-}\\ s.t.\;\|\varphi ({\tilde{X}}_{j}^{-})-C^{-}{\|}^{2}\leq (R^{-}{)}^{2}+\xi_{j}^{-},\|\varphi ({\tilde{X}}_{j}^{-})-C^{-}{\|}^{2}\geq (R^{-}{)}^{2}-\frac{1}{\tau }\xi_{j}^{-},\\ \;\|\varphi ({\tilde{X}}_{i}^{+})-C^{-}{\|}^{2}\geq ({\hat{R}}^{-}{)}^{2}-{\hat{\xi }}_{i}^{-},\|\varphi ({\tilde{X}}_{i}^{+}-C^{-}){\|}^{2}\leq ({\hat{R}}^{-}{)}^{2}+\frac{1}{\tau }{\hat{\xi }}_{i}^{-},\\ \;\|\varphi ({\bar{X}}_{l}^{-})-C^{-}{\|}^{2}=\varsigma_{l}^{-},({\hat{R}}^{-}{)}^{2}-(R^{-}{)}^{2}\geq 0,\\ \;j=1,2,\cdots ,{\tilde{m}}^{-},i=1,2,\cdots ,{\tilde{m}}^{+},l=1,2,\cdots ,{\bar{m}}^{-}, \end{array}$$ (22)

where ${\tilde{X}}^{+}$ and ${\bar{X}}^{+}$ represent two datasets in class +1. ${\tilde{X}}^{-}$ and ${\bar{X}}^{-}$ represent two datasets in class -1. The numbers of samples in four datasets are ${\tilde{m}}^{+}$, ${\tilde{m}}^{-}$, ${\bar{m}}^{+}$ and ${\bar{m}}^{-}$ respectively. For QHSVM, these datasets are specified as ***X***^+^ or ***X***^−^. So, for QHSVM, QPPs (21) and (22) need to satisfy the following condition:

$$\begin{array}{l} {\tilde{X}}^{+}=X^{+},{\bar{X}}^{+}=X^{+},{\tilde{X}}^{-}=X^{-},{\bar{X}}^{-}=X^{-},\\ {\tilde{m}}^{+}={\bar{m}}^{+}=m^{+},{\tilde{m}}^{-}={\bar{m}}^{-}=m^{-}. \end{array}$$ (23)

For QPP (21) with the condition (23), $X_{i}^{+}$ is the target sample, and $X_{j}^{-}$ is the negative sample. QPP (21) searches for two quantile hyper-spheres: Ω^+^ and ${\hat{\Omega }}^{+}$. Their radii are *R*^+^ and ${\hat{R}}^{+}$. And the two hyper-spheres have the same center ***C***^+^. The first term of objective function in QPP (21) minimizes (*R*^+^)^2^, which tends to keep the volume of Ω^+^ as small as possible. The second term maximizes $({\hat{R}}^{+}{)}^{2}$, which is to force the volume of ${\hat{\Omega }}^{+}$ as big as possible. On the other hand, minimizing (*R*^+^)^2^ and maximizing $({\hat{R}}^{+}{)}^{2}$ mean to keep the margin between Ω^+^ and ${\hat{\Omega }}^{+}$ as big as possible, which embodies the margin maximization principle. The first and the second constraint conditions in QPP (21) make Ω^+^ be a quantile hyper-sphere controlled by *τ* instead of boundary hyper-sphere because some target samples fall outside of Ω^+^. The third and the fourth constraint conditions in QPP (21) also make ${\hat{\Omega }}^{+}$ be a quantile hyper-sphere controlled by *τ* because some negative samples fall inside of ${\hat{\Omega }}^{+}$. These constraints make the maximum margin depend on many samples instead of few samples, which ensures QPP (21) is insensitive to noise, especially the feature noise around the decision boundary. The third and the fourth terms of objective function in QPP (21) are to minimize the sum of slack variables caused by some samples not satisfying the constraint conditions. The fifth term and constraint condition require the target samples to be distributed in the center of Ω^+^ as much as possible. In other words, the center of Ω^+^ is close to the cluster of target samples. This means our QHSVM exploits the prior structural information of target samples. Our QHSVM should be not sensitive to the structure of the data distribution. So, the term ensures that the inner-class clustering of samples is maximized. The last constraint condition ensures the radius of Ω^+^ is not smaller than that of ${\hat{\Omega }}^{+}$. $c_{2}^{+}$, $c_{3}^{+}$ and *v*^+^ are trade-off parameters.

For QPP (22) with the condition (23), $X_{j}^{-}$ is the target sample, and $X_{i}^{+}$ is the negative sample. QPP (22) is similar to QPP (21) in attribute and conclusion. So, it is not necessary to analyze again.

Similar to TWSVM, QHSVM builds two support hyper-spheres for binary classification. For QPP (21) with the condition (23), Ω^+^ with parameters ***C***^+^ and *R*^+^ is referred to as the support hyper-sphere of the target sample $X_{i}^{+}$. The negative sample $X_{j}^{-}$ is only used to improve the description of Ω^+^. Ω^+^ is described by using the margin maximization principle and inner-class clustering maximization of samples. It is insensitive to noise. $X_{j}^{-}$ is only used to implement the margin maximization principle. Similarly, for QPP (22) with the condition (23), Ω^−^ with parameters ***C***^−^ and *R*^−^ is reckoned as the support hyper-sphere of $X_{j}^{-}$. $X_{i}^{+}$ is only used to improve the description of Ω^−^. All mentioned above is helpful to improve the generalization of QHSVM.

For binary QHSVM, the following decision function can be obtained.

$$f(x)=\arg\;\min\{\frac{\|\varphi (x)-C^{+}{\|}^{2}}{{\left(R^{+}\right)}^{2}},\frac{\|\varphi (x)-C^{-}{\|}^{2}}{{\left(R^{-}\right)}^{2}}\}.$$ (24)

### 3.3 QHSVM with surrounding and clustering samples

For QPPs (21) and (22) with the condition (23), all training samples are used for optimization with inequality constraints, which means QHSVM is fit for classification without high efficiency requirement. For a highly efficient classification problem, we provide a QHSVM with surrounding and clustering samples, which is called *ρ*-QHSVM. The surrounding samples refer to samples that are distributed near the boundary of the quantile hyper-spheres. In the case of ***X***^+^, its surrounding samples are distributed near the boundary of Ω^+^. The clustering samples refer to samples that are distributed near the center of the quantile hyper-spheres. The quantile hyper-spheres of *ρ*-QHSVM can be obtained by using sparse surrounding samples rather than all samples. So, these training samples should be divided into surrounding samples and clustering samples. In order to achieve it, a novel local center-based density estimation method is proposed.

Local center-based density estimation is originated from kernel density estimation in [32]. Kernel density estimation yields Gaussian weight by calculating the distance between a sample and its *K*-nearest neighbors. This kernel density weight can efficiently characterize the local geometry of samples manifold, but it can't capture surrounding samples in the training dataset. So, the local center-based density estimation method is designed.

Consider a training dataset ***X*** = {***X***_*i*_|*i* = 1,2,⋯,m}. Firstly, the kernel function *Ψ*(***X***_*i*_,***X***_*l*_) = *φ*(***X***_*i*_)⋅*φ*(***X***_*l*_) is introduced. Then, the steps for a local center-based density estimation method are given in nonlinear feature mapping space:

Step 1: Calculate the square distance between each sample ***X***_*i*_ and the others.

$$d_{il}^{2}=\|\varphi (X_{i})-\varphi (X_{l}){\|}^{2},where\;l=1,2,\cdots ,m.$$ (25)

Step2: Search for *K*-nearest neighbors in nonlinear feature mapping space for each sample ***X***_*i*_.

$$N_{i}=\left\{X_{i_{k}}|d_{ii_{k}}^{2}<d_{il}^{2};i_{k},l\in \{1,2,\cdots ,m\};i_{k}\neq l;i_{k},l\neq i;k=1,2,\cdots ,K\right\}$$ (26)

Step 3: Calculate the mean of square distances for the training dataset.

$${\bar{d}}^{2}=\frac{1}{Km}\sum_{i=1}^{m}\sum_{k=1}^{K}d_{ii_{k}}^{2}.$$ (27)

Step 4: Calculate the kernel density weight for each sample ***X***_*i*_.

$$\rho_{i}^{w}=1+w_{i},where\;w_{i}=\sum_{k=1}^{K}e^{-\frac{\|\varphi (X_{i})-\varphi (X_{i_{k}}){\|}^{2}}{{\bar{d}}^{2}}}.$$ (28)

Step 5: Determine the center of *K*-nearest neighbors for sample ***X***_*i*_.

$$\varphi (X_{ic})=\frac{1}{K}\sum_{k=1}^{K}\varphi (X_{i_{k}}).$$ (29)

Step 6: The local center-based density of ***X***_*i*_ is estimated as follows.

$$\rho_{i}=1+q_{i}w_{i},where\;q_{i}=e^{-\frac{\|\varphi (X_{i})-\varphi (X_{ic}){\|}^{2}}{{\bar{d}}^{2}}}.$$ (30)

**Fig 2:**
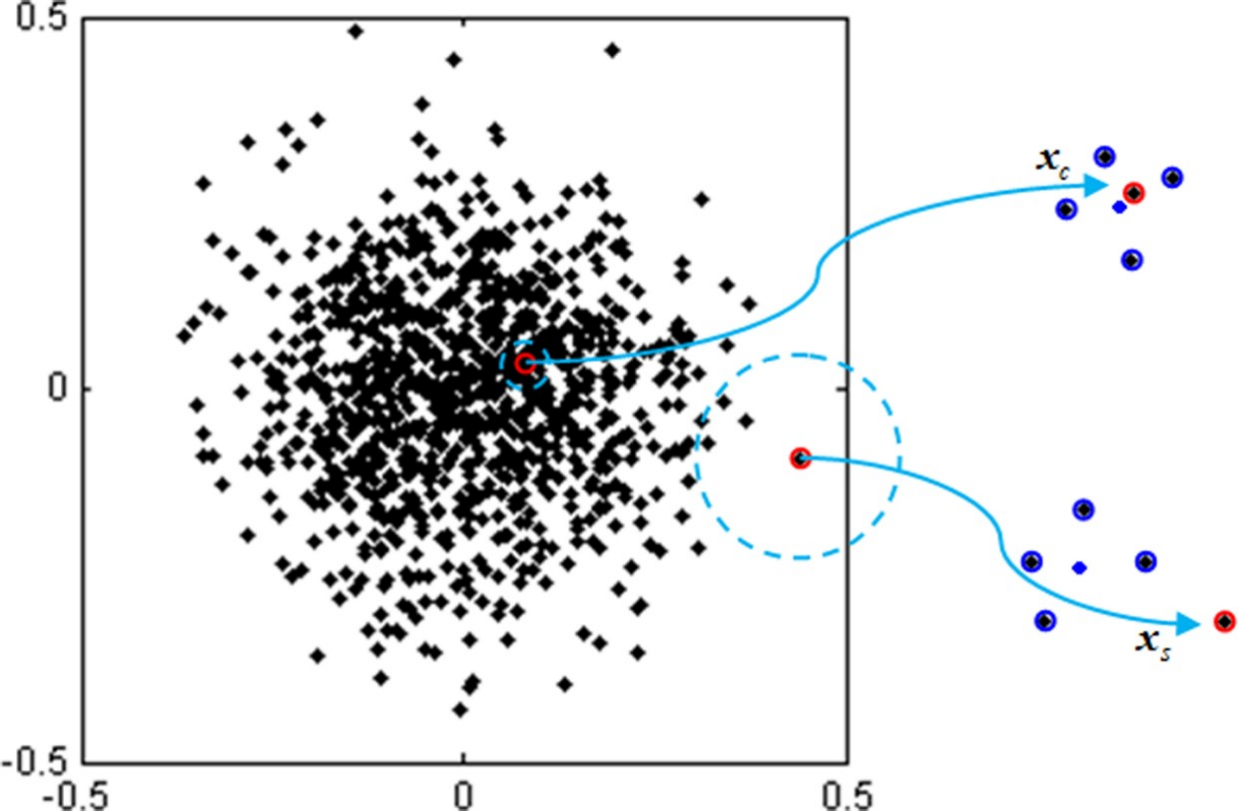
The illustration of surrounding sample, clustering sample and the centers of *K*-nearest neighbors. The surrounding sample *x*_*s*_ and the clustering sample *x*_*c*_ (black dots with red circles), *K*-nearest neighbors (black dots with blue circles) and the centers of *K*-nearest neighbors (blue dots).

It can be seen from the above steps that the local center-based density of ***X***_*i*_ is estimated with the distance between the sample and its *K*-nearest neighbors, where *K* is given by user. Moreover, the local center-based density is a Gaussian kernel density. When *q*_*i*_ = 1, $\rho_{i}=\rho_{i}^{w}$. A bigger $\rho_{i}^{w}$ indicates that ***X***_*i*_ is closer to its *K*-nearest neighbors. So, $\rho_{i}^{w}$ can be used to check if ***X***_*i*_ is a clustering sample or an isolated sample. However, $\rho_{i}^{w}$ can’t be used to identify surrounding samples. The training dataset can be divided into clustering samples and surrounding samples from center to outside. The surrounding samples distributed near the boundary of the quantile hyper-spheres deviate the center of *K*-nearest neighbors. **Fig 2** shows that the surrounding sample ***x***_*s*_ is far from the center of *K*-nearest neighbors, while the clustering sample ***x***_*c*_ is close to that center of *K*-nearest neighbors. This is their distinctive characteristics. So, *q*_*i*_ is used to represent the deviation degree. When *q*_*i*_≠1, each *ρ*_*i*_ must be compensated with *q*_*i*_. *ρ*_*i*_ is called as local center-based density. The smaller *ρ*_*i*_ is, the closer ***X***_*i*_ is to boundary. The bigger *ρ*_*i*_ is, the closer ***X***_*i*_ is to clustering region. On the other hand, Gaussian kernel parameter *δ*^2^ is set as ${\bar{d}}^{2}$. ${\bar{d}}^{2}$ is the mean of square distances for the training dataset, which makes (30) fit for different training datasets with different clustering degrees.

**Fig 3:**
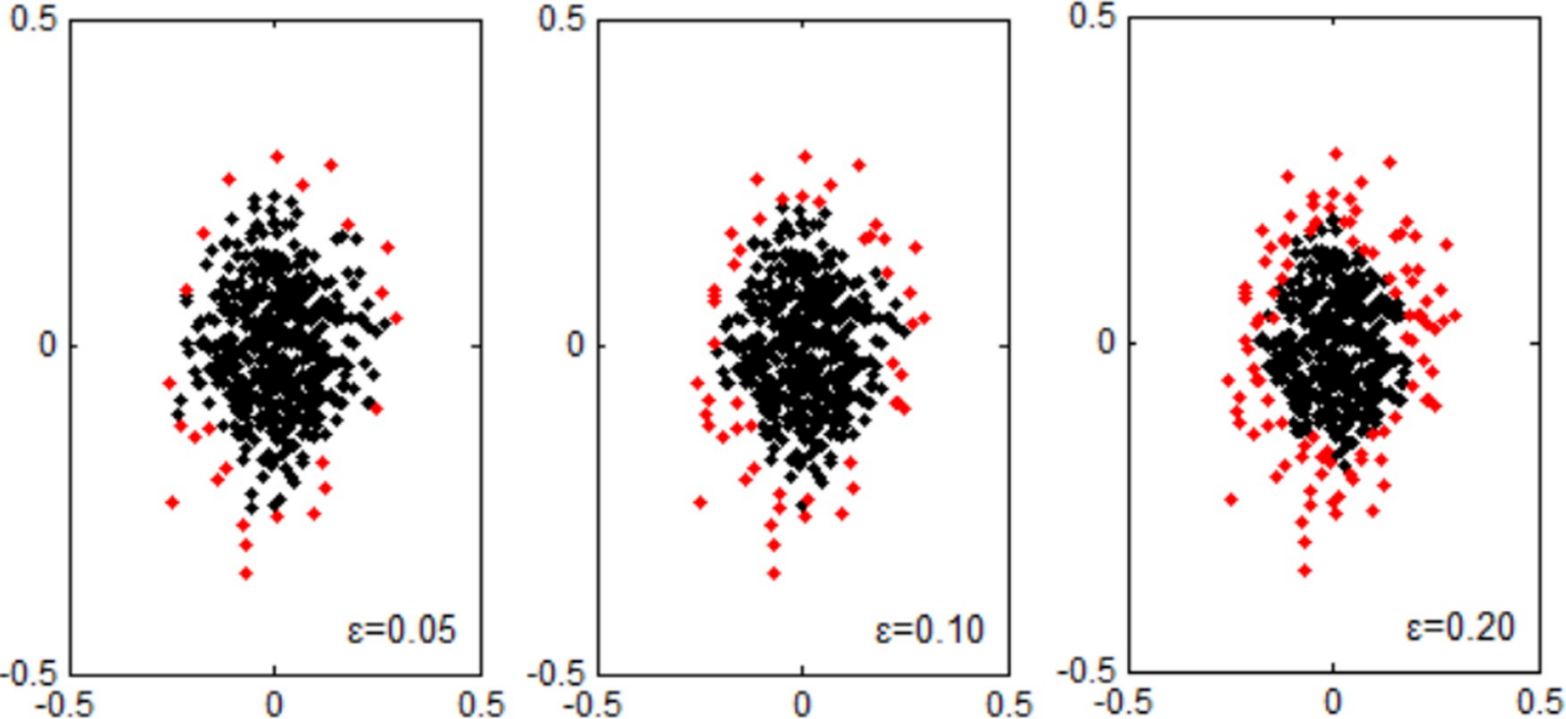
The illustration of surrounding samples and clustering samples with different *ε* in two-dimensional feature space. The surrounding samples (“**·**”in red) and the clustering samples (“**·**” in black).

For binary classification, consider datasets $X^{+}=\{X_{i}^{+}|i=1,2,\cdots ,m^{+}\}$ and $X^{-}=\{X_{j}^{-}|j=1,2,\cdots ,m^{-}\}$. Their local center-based density $\rho_{i}^{+}$ and $\rho_{j}^{-}$ are estimated with (28). Based on magnitude of $\rho_{i}^{+}$, ***X***^+^ is divided into ${\tilde{X}}^{+}$ and ${\bar{X}}^{+}$ by ratio *ε*(0<*ε*<1).

$$\begin{array}{l} {\tilde{X}}^{+}=\{{\tilde{X}}_{\tilde{i}}^{+}|\tilde{i}=1,2,\cdots ,{\tilde{m}}^{+}\},{\bar{X}}^{+}=\{{\bar{X}}_{\bar{i}}^{+}|\bar{i}=1,2,\cdots ,{\bar{m}}^{+}\},\\ X^{+}=[{\tilde{X}}^{+},{\bar{X}}^{+}],m^{+}={\tilde{m}}^{+}+{\bar{m}}^{+},\\ \varepsilon =\frac{{\tilde{m}}^{+}}{m^{+}},{\tilde{\rho }}_{\tilde{i}}^{+}<{\bar{\rho }}_{\bar{i}}^{+}, \end{array}$$ (31)

where ${\tilde{\rho }}_{\tilde{i}}^{+}$ and ${\bar{\rho }}_{\bar{i}}^{+}$ are the local center-based densities of ${\tilde{X}}_{\tilde{i}}^{+}$ and ${\bar{X}}_{\bar{i}}^{+}$ respectively. The principle of division is ${\tilde{\rho }}_{\tilde{i}}^{+}<{\bar{\rho }}_{\bar{i}}^{+}$. So, ${\tilde{X}}_{\tilde{i}}^{+}$ is a surrounding sample with small local center-based density. And ${\bar{X}}_{\bar{i}}^{+}$ is a clustering sample with big local center-based density. **Fig 3** illustrates the results of surrounding samples and clustering samples with different *ε* in two-dimensional feature space. It can be seen that the surrounding samples distribute near the boundary of two-dimensional dataset. Similarly, based on magnitude of $\rho_{j}^{-}$, ***X***^−^ is divided into ${\tilde{X}}^{-}$ and ${\bar{X}}^{-}$ according to ratio *ε*.

$$\begin{array}{l} {\tilde{X}}^{-}=\{{\tilde{X}}_{\tilde{j}}^{-}|\tilde{j}=1,2,\cdots ,{\tilde{m}}^{-}\},{\bar{X}}^{-}=\{{\bar{X}}_{\bar{j}}^{-}|\bar{j}=1,2,\cdots ,{\bar{m}}^{-}\},\\ X^{-}=[{\tilde{X}}^{-},{\bar{X}}^{-}],m^{-}={\tilde{m}}^{-}+{\bar{m}}^{-},\\ \varepsilon =\frac{{\tilde{m}}^{-}}{m^{-}},{\tilde{\rho }}_{\tilde{j}}^{-}<{\bar{\rho }}_{\bar{j}}^{-}, \end{array}$$ (32)

where ${\tilde{\rho }}_{\tilde{i}}^{+}$ and ${\bar{\rho }}_{\bar{i}}^{+}$ are the local center-based densities of ${\tilde{X}}_{\tilde{i}}^{+}$ and ${\bar{X}}_{\bar{i}}^{+}$ respectively. The principle of division is ${\tilde{\rho }}_{\tilde{j}}^{-}<{\bar{\rho }}_{\bar{j}}^{-}$.

Based on ${\tilde{X}}^{+}$, ${\bar{X}}^{+}$, ${\tilde{X}}^{-}$ and ${\bar{X}}^{-}$, the two QPPs of *ρ*-QHSVM are expressed as (21) and (22). Comparing with QHSVM, $X_{i}^{+}$ and $X_{j}^{-}$ are changed as ${\tilde{X}}_{i}^{+}$ and ${\tilde{X}}_{j}^{-}$ respectively. ${\tilde{X}}^{+}$ and ${\tilde{X}}^{-}$ are sparse datasets because the number of samples is reduced greatly. And their sparseness is controlled by *ε*. This means that the number of samples with inequality constraints is greatly reduced. So, the optimization speed of *ρ*-QHSVM is improved. Moreover, it can be seen from QPP (21) that the optimization accuracy is controlled by boundary samples. So, for QPP (21), the surrounding samples sets $\left\{{\tilde{X}}_{i}^{+}|i=1,2,\cdots ,{\tilde{m}}^{+}\right\}$ and $\left\{{\tilde{X}}_{j}^{-}|j=1,2,\cdots ,{\tilde{m}}^{-}\right\}$ ensure the optimization accuracy because they include boundary samples. On the other hand, comparing with QHSVM, $X_{i}^{+}$ is changed as ${\bar{X}}_{l}^{+}$, which shows that the center of the support hyper-sphere is closer to the samples with higher clustering degree. And ${\bar{X}}^{+}$ is a sparse dataset, because the number of samples is also reduced. So, the clustering samples improve optimization speed and accuracy with equality constraints. For QPP (22), similar attributes and conclusions can be obtained. In summary, *ρ*-QHSVM is fit for high efficiency classification.

## 4. Solution to *ρ*-QHSVM

Comparing with *ρ*-QHSVM, QHSVM has the additional condition (23). So, QHSVM can be considered as a special case of *ρ*-QHSVM. So, the solution of *ρ*-QHSVM is only given in this section. And the solution of QHSVM can be obtained from *ρ*-QHSVM.

In order to solve QPPs of *ρ*-QHSVM classifier, operators $\alpha_{i}^{+}\geq 0$, $\beta_{i}^{+}\geq 0$, ${\hat{\alpha }}_{j}^{+}\geq 0$, ${\hat{\beta }}_{j}^{+}\geq 0$ and *γ*≥0 are introduced. The Lagrangian function of QPP (21) is

$$\begin{array}{l} L={\left(R^{+}\right)}^{2}-({\hat{R}}^{+}{)}^{2}+c_{2}^{+}\sum_{i=1}^{{\tilde{m}}^{+}}\xi_{i}^{+}+c_{3}^{+}\sum_{j=1}^{{\tilde{m}}^{-}}{\hat{\xi }}_{j}^{+}+v^{+}\sum_{l=1}^{{\bar{m}}^{+}}\|\varphi ({\bar{X}}_{l}^{+})-C^{+}{\|}^{2}-\gamma \left(({\hat{R}}^{+}{)}^{2}-(R^{+}{)}^{2}\right)\\ \;-\sum_{i=1}^{{\tilde{m}}^{+}}\alpha_{i}^{+}\left((R^{+}{)}^{2}-\|\varphi ({\tilde{X}}_{i}^{+})-C^{+}{\|}^{2}+\xi_{i}^{+}\right)-\sum_{i=1}^{{\tilde{m}}^{+}}\beta_{i}^{+}\left(\|\varphi ({\tilde{X}}_{i}^{+})-C^{+}{\|}^{2}-{\left(R^{+}\right)}^{2}+\frac{1}{\tau }\xi_{i}^{+}\right)\\ \;-\sum_{j=1}^{{\tilde{m}}^{-}}{\hat{\alpha }}_{j}^{+}\left(\|\varphi ({\tilde{X}}_{j}^{-})-C^{+}{\|}^{2}-{\left({\hat{R}}^{+}\right)}^{2}+{\hat{\xi }}_{j}^{+}\right)-\sum_{j=1}^{{\tilde{m}}^{-}}{\hat{\beta }}_{j}^{+}\left({\left({\hat{R}}^{+}\right)}^{2}-\|\varphi ({\tilde{X}}_{j}^{-})-C^{+}{\|}^{2}+\frac{1}{\tau }{\hat{\xi }}_{j}^{+}\right). \end{array}$$ (33)

Then, the KKT necessary and sufficient optimality conditions for QPP (21) are given by

$$\begin{array}{l} \frac{\partial L}{\partial C^{+}}=-2v^{+}\sum_{l=1}^{{\bar{m}}^{+}}\left(\varphi ({\bar{X}}_{l}^{+})-C^{+}\right)-2\sum_{i=1}^{{\tilde{m}}^{+}}\alpha_{i}^{+}\left(\varphi ({\tilde{X}}_{i}^{+})-C^{+}\right)+2\sum_{i=1}^{{\tilde{m}}^{+}}\beta_{i}^{+}\left(\varphi ({\tilde{X}}_{i}^{+})-C^{+}\right)\\ \;+2\sum_{j=1}^{{\tilde{m}}^{-}}{\hat{\alpha }}_{j}^{+}\left(\varphi ({\tilde{X}}_{j}^{-})-C^{+}\right)-2\sum_{j=1}^{{\tilde{m}}^{-}}{\hat{\beta }}_{j}^{+}\left(\varphi ({\tilde{X}}_{j}^{-})-C^{+}\right)=0, \end{array}$$ (34)

$$\frac{\partial L}{\partial {\left(R^{+}\right)}^{2}}=1-\sum_{i=1}^{{\tilde{m}}^{+}}\alpha_{i}^{+}+\sum_{i=1}^{{\tilde{m}}^{+}}\beta_{i}^{+}+\gamma =0,$$ (35)

$$\frac{\partial L}{\partial ({\hat{R}}^{+}{)}^{2}}=-1+\sum_{j=1}^{{\tilde{m}}^{-}}{\hat{\alpha }}_{j}^{+}-\sum_{j=1}^{{\tilde{m}}^{-}}{\hat{\beta }}_{j}^{+}-\gamma =0,$$ (36)

$$\frac{\partial L}{\partial \xi_{i}^{+}}=c_{2}^{+}-\alpha_{i}^{+}-\frac{1}{\tau }\beta_{i}^{+}=0,$$ (37)

$$\frac{\partial L}{\partial {\hat{\xi }}_{j}^{+}}=c_{3}^{+}-{\hat{\alpha }}_{j}^{+}-\frac{1}{\tau }{\hat{\beta }}_{j}^{+}=0,$$ (38)

$$\alpha_{i}^{+}\left({\left(R^{+}\right)}^{2}-\|{\tilde{X}}_{i}^{+}-C^{+}{\|}^{2}+\xi_{i}^{+}\right)=0,\beta_{i}^{+}\left(\|{\tilde{X}}_{i}^{+}-C^{+}{\|}^{2}-(R^{+}{)}^{2}+\frac{1}{\tau }\xi_{i}^{+}\right)=0,$$ (39)

$${\hat{\alpha }}_{j}^{+}\left(\|{\tilde{X}}_{j}^{-}-C^{+}{\|}^{2}-({\hat{R}}^{+}{)}^{2}+{\hat{\xi }}_{j}^{+}\right)=0,{\hat{\beta }}_{j}^{+}\left(({\hat{R}}^{+}{)}^{2}-\|{\tilde{X}}_{j}^{-}-C^{+}{\|}^{2}+\frac{1}{\tau }{\hat{\xi }}_{j}^{+}\right)=0,$$ (40)

$$\gamma \left(({\hat{R}}^{+}{)}^{2}-{\left(R^{+}\right)}^{2}\right)=0,$$ (41)

$$\alpha_{i}^{+}\geq 0,\;\beta_{i}^{+}\geq 0\;{\hat{\alpha }}_{j}^{+}\geq 0,\;{\hat{\beta }}_{j}^{+}\geq 0,\;\gamma \geq 0.$$ (42)

Define

$$\lambda_{i}^{+}=\alpha_{i}^{+}-\beta_{i}^{+}\;\mathrm{and}\;{\hat{\lambda }}_{j}^{+}={\hat{\alpha }}_{j}^{+}-{\hat{\beta }}_{j}^{+}.$$ (43)

From (35) and (36), it follows that

$$\sum_{i=1}^{{\tilde{m}}^{+}}\left(\alpha_{i}^{+}-\beta_{i}^{+}\right)-\sum_{j=1}^{{\tilde{m}}^{-}}\left({\hat{\alpha }}_{j}^{+}-{\hat{\beta }}_{j}^{+}\right)=\sum_{i=1}^{{\tilde{m}}^{+}}\lambda_{i}^{+}-\sum_{j=1}^{{\tilde{m}}^{-}}{\hat{\lambda }}_{j}^{+}=0,$$ (44)

$$\overset{{\tilde{m}}^{+}}{\underset{i=1}{\sum }}\lambda_{i}^{+}\geq 1,\overset{{\tilde{m}}^{-}}{\underset{j=1}{\sum }}{\hat{\lambda }}_{j}^{+}\geq 1.$$ (45)

According to (37), (38), (42) and (43), we can get

$$-\tau c_{2}^{+}\leq \lambda_{i}^{+}\leq c_{2}^{+},-\tau c_{3}^{+}\leq {\hat{\lambda }}_{j}^{+}\leq c_{3}^{+}.$$ (46)

Let $p^{+}=1/v^{+}{\bar{m}}^{+}$. Then, (34) can be rewritten as

$$C^{+}=p^{+}\left(v^{+}\sum_{l=1}^{{\bar{m}}^{+}}\varphi ({\bar{X}}_{l}^{+})+\sum_{i=1}^{{\tilde{m}}^{+}}\lambda_{i}^{+}\varphi ({\tilde{X}}_{i}^{+})-\sum_{j=1}^{{\tilde{m}}^{-}}{\hat{\lambda }}_{j}^{+}\varphi ({\tilde{X}}_{j}^{-})\right).$$ (47)

Substituting (35), (36), (37) and (38) into (33) leads to

$$\begin{array}{l} L=v^{+}\sum_{l=1}^{{\bar{m}}^{+}}\|\varphi ({\bar{X}}_{l}^{+})-C^{+}{\|}^{2}+\sum_{i=1}^{{\tilde{m}}^{+}}\alpha_{i}^{+}\|\varphi ({\tilde{X}}_{i}^{+})-C^{+}{\|}^{2}-\sum_{i=1}^{{\tilde{m}}^{+}}\beta_{i}^{+}\|\varphi ({\tilde{X}}_{i}^{+})-C^{+}{\|}^{2}\\ \;-\sum_{j=1}^{{\tilde{m}}^{-}}{\hat{\alpha }}_{j}^{+}\|\varphi ({\tilde{X}}_{j}^{-})-C^{+}{\|}^{2}+\sum_{j=1}^{{\tilde{m}}^{-}}{\hat{\beta }}_{j}^{+}\|\varphi ({\tilde{X}}_{j}^{-})-C^{+}{\|}^{2}. \end{array}$$ (48)

By substituting (44), (45), (46) and (47) into (48), the dual QPP of (21) can be changed to

$$\begin{array}{l} \underset{\lambda_{i}^{+},{\hat{\lambda }}_{j}^{+}}{\max}L=\sum_{i=1}^{{\tilde{m}}^{+}}\lambda_{i}^{+}\Psi ({\tilde{X}}_{i}^{+},{\tilde{X}}_{i}^{+})-\sum_{j=1}^{{\tilde{m}}^{-}}{\hat{\lambda }}_{j}^{+}\Psi ({\tilde{X}}_{j}^{-},{\tilde{X}}_{j}^{-})-p^{+}\left(\sum_{i1=1}^{{\tilde{m}}^{+}}\sum_{i2=1}^{{\tilde{m}}^{+}}\lambda_{i1}^{+}\lambda_{i2}^{+}\Psi ({\tilde{X}}_{i1}^{+},{\tilde{X}}_{i2}^{+})+\sum_{j1=1}^{{\tilde{m}}^{-}}\sum_{j2=1}^{{\tilde{m}}^{-}}{\hat{\lambda }}_{j1}^{+}{\hat{\lambda }}_{j2}^{+}\Psi ({\tilde{X}}_{j1}^{-},{\tilde{X}}_{j2}^{-})\right)\\ \;-p^{+}\left(2v^{+}\sum_{i1=1}^{{\tilde{m}}^{+}}\sum_{l=1}^{{\bar{m}}^{+}}\lambda_{i1}^{+}\Psi ({\tilde{X}}_{i1}^{+},{\bar{X}}_{l}^{+})-2v^{+}\sum_{l=1}^{{\bar{m}}^{+}}\sum_{j=1}^{{\tilde{m}}^{-}}{\hat{\lambda }}_{j}^{+}\Psi ({\bar{X}}_{l}^{+},{\tilde{X}}_{j}^{-})-2\sum_{i=1}^{{\tilde{m}}^{+}}\sum_{j=1}^{{\tilde{m}}^{-}}\lambda_{i}^{+}{\hat{\lambda }}_{j}^{+}\Psi ({\tilde{X}}_{i}^{+},{\tilde{X}}_{j}^{-})\right)\\ s.t.\;\sum_{i=1}^{{\tilde{m}}^{+}}\lambda_{i}^{+}-\sum_{j=1}^{{\tilde{m}}^{-}}{\hat{\lambda }}_{j}^{+}=0,\\ \;\sum_{i=1}^{{\tilde{m}}^{+}}\lambda_{i}^{+}\geq 1,\sum_{j=1}^{{\tilde{m}}^{-}}{\hat{\lambda }}_{j}^{+}\geq 1,\\ \;-\tau c_{2}^{+}\leq \lambda_{i}^{+}\leq c_{2}^{+},-\tau c_{3}^{+}\leq {\hat{\lambda }}_{j}^{+}\leq c_{3}^{+},\\ \;i=1,2,\cdots ,{\tilde{m}}^{+},j=1,2,\cdots ,{\tilde{m}}^{-}. \end{array}$$ (49)

The matrix form for QPP (49) can be expressed as

$$\begin{array}{l} \underset{\mu^{+}}{\min}{\left(\mu^{+}\right)}^{T}Q^{+}\mu^{+}+F^{+}\mu^{+}\\ s.t.\;-\tau V^{+}\leq \mu^{+}\leq V^{+},\\ \;A^{+}\mu^{+}\leq B^{+},\\ \;E^{+}\mu^{+}=0, \end{array}$$ (50)

where

$$\mu^{+}=\left[\begin{array}{l} \lambda^{+}\\ {\hat{\lambda }}^{+} \end{array}\right],\;\lambda^{+}=[\lambda_{1}^{+},\lambda_{2}^{+},\cdots ,\lambda_{{\tilde{m}}^{+}}^{+}],\;{\hat{\lambda }}^{+}=[{\hat{\lambda }}_{1}^{+},{\hat{\lambda }}_{2}^{+},\cdots ,{\hat{\lambda }}_{{\tilde{m}}^{-}}^{+}],$$ (51)

$$Q^{+}=p^{+}\left[\begin{array}{cc} \Psi ({\tilde{X}}^{+},{\tilde{X}}^{+}) & -\Psi ({\tilde{X}}^{+},{\tilde{X}}^{-})\\ -\Psi ({\tilde{X}}^{-},{\tilde{X}}^{+}) & \Psi ({\tilde{X}}^{-},{\tilde{X}}^{-}) \end{array}\right],$$ (52)

$$F^{+}=-{\left[\begin{array}{l} diag\left(\Psi ({\tilde{X}}^{+},{\tilde{X}}^{+})\right)-{\left(2p^{+}v^{+}{\left({\bar{e}}^{+}\right)}^{T}\Psi ({\bar{X}}^{+},{\tilde{X}}^{+})\right)}^{T}\\ -diag\left(\Psi ({\tilde{X}}^{-},{\tilde{X}}^{-})\right)+{\left(2p^{+}v^{+}{\left({\bar{e}}^{+}\right)}^{T}\Psi ({\bar{X}}^{+},{\tilde{X}}^{-})\right)}^{T} \end{array}\right]}^{T},$$ (53)

$$V^{+}=\left[\begin{array}{l} c_{2}^{+}{\tilde{e}}^{+}\\ c_{3}^{+}{\tilde{e}}^{-} \end{array}\right],$$ (54)

$$E^{+}={\left[\begin{array}{c} {\tilde{e}}^{+}\\ -{\tilde{e}}^{-} \end{array}\right]}^{T},$$ (55)

$$A^{+}=\left[\begin{array}{cc} -{\left({\tilde{e}}^{+}\right)}^{T} & 0^{1\times {\tilde{m}}^{-}}\\ 0^{1\times {\tilde{m}}^{+}} & -{\left({\tilde{e}}^{-}\right)}^{T} \end{array}\right],\;B^{+}=-\left[\begin{array}{c} 1\\ 1 \end{array}\right],$$ (56)

$${\tilde{e}}^{+}=[1,1,\cdots {,1]}^{T}\in {ℜ}^{{\tilde{m}}^{+}\times 1},\;{\bar{e}}^{+}=[1,1,\cdots {,1]}^{T}\in {ℜ}^{{\bar{m}}^{+}\times 1},\;{\tilde{e}}^{-}=[1,1,\cdots {,1]}^{T}\in {ℜ}^{{\tilde{m}}^{-}\times 1}.$$ (57)

Similarly, by taking (22) into account, the dual QPP can be expressed as

$$\begin{array}{l} \underset{\lambda_{j}^{-},{\hat{\lambda }}_{i}^{-}}{\max}L=\sum_{j=1}^{{\tilde{m}}^{-}}\lambda_{j}^{-}\Psi ({\tilde{X}}_{j}^{-},{\tilde{X}}_{j}^{-})-\sum_{i=1}^{{\tilde{m}}^{+}}{\hat{\lambda }}_{i}^{-}\Psi ({\tilde{X}}_{i}^{+},{\tilde{X}}_{i}^{+})-p^{-}\left(\sum_{j1=1}^{{\tilde{m}}^{-}}\sum_{j2=1}^{{\tilde{m}}^{-}}\lambda_{j1}^{-}\lambda_{j2}^{-}\Psi ({\tilde{X}}_{j1}^{-},{\tilde{X}}_{j2}^{-})+\sum_{i1=1}^{{\tilde{m}}^{+}}\sum_{i2=1}^{{\tilde{m}}^{+}}{\hat{\lambda }}_{i1}^{-}{\hat{\lambda }}_{i2}^{-}\Psi ({\tilde{X}}_{i1}^{+},{\tilde{X}}_{i2}^{+})\right)\\ \;-p^{-}\left(2v^{-}\sum_{j1=1}^{{\tilde{m}}^{-}}\sum_{l=1}^{{\bar{m}}^{-}}\lambda_{j1}^{-}\Psi ({\tilde{X}}_{j1}^{-},{\bar{X}}_{l}^{-})-2v^{-}\sum_{l=1}^{{\bar{m}}^{-}}\sum_{i=1}^{{\tilde{m}}^{+}}{\hat{\lambda }}_{i}^{-}\Psi ({\bar{X}}_{l}^{-},{\tilde{X}}_{i}^{+})-2\sum_{j=1}^{{\tilde{m}}^{-}}\sum_{i=1}^{{\tilde{m}}^{+}}\lambda_{j}^{-}{\hat{\lambda }}_{i}^{-}\Psi ({\tilde{X}}_{j}^{-},{\tilde{X}}_{i}^{+})\right)\\ s.t.\;\sum_{j=1}^{{\tilde{m}}^{-}}\lambda_{j}^{-}-\sum_{i=1}^{{\tilde{m}}^{+}}{\hat{\lambda }}_{i}^{-}=0,\\ \;\sum_{j=1}^{{\tilde{m}}^{-}}\lambda_{j}^{-}\geq 1,\sum_{i=1}^{{\tilde{m}}^{+}}{\hat{\lambda }}_{i}^{-}\geq 1,\\ \;-\tau c_{2}^{-}\leq \lambda_{j}^{-}\leq c_{2}^{-},-\tau c_{3}^{-}\leq {\hat{\lambda }}_{i}^{-}\leq c_{3}^{-},\\ \;j=1,2,\cdots ,{\tilde{m}}^{-},i=1,2,\cdots ,{\tilde{m}}^{+}. \end{array}$$ (58)

where $p^{-}=1/v^{-}{\bar{m}}^{-}$, $\lambda_{j}^{-}=\alpha_{j}^{-}-\beta_{j}^{-}$ and ${\hat{\lambda }}_{i}^{-}={\hat{\alpha }}_{i}^{-}-{\hat{\beta }}_{i}^{-}.$ $\alpha_{j}^{-}\geq 0$, $\beta_{j}^{-}\geq 0$, ${\hat{\alpha }}_{i}^{-}\geq 0$ and ${\hat{\beta }}_{i}^{-}\geq 0$ are Lagrangian operators. And

$$C^{-}=p^{-}\left(v^{-}\sum_{l=1}^{{\bar{m}}^{-}}\varphi ({\bar{X}}_{l}^{-})+\sum_{j=1}^{{\tilde{m}}^{-}}\lambda_{j}^{-}\varphi ({\tilde{X}}_{j}^{-})-\sum_{i=1}^{{\tilde{m}}^{+}}{\hat{\lambda }}_{i}^{-}\varphi ({\tilde{X}}_{i}^{+})\right).$$ (59)

The matrix form for QPP (58) can be expressed as

$$\begin{array}{l} \underset{\mu^{-}}{\min}{\left(\mu^{-}\right)}^{T}Q^{-}\mu^{-}+F^{-}\mu^{-}\\ s.t.\;-\tau V^{-}\leq \mu^{-}\leq V^{-},\\ \;A^{-}\mu^{-}\leq B^{-},\\ \;E^{-}\mu^{-}=0, \end{array}$$ (60)

where

$$\mu^{-}=\left[\begin{array}{l} \lambda^{-}\\ {\hat{\lambda }}^{-} \end{array}\right],\;\lambda^{-}=[\lambda_{1}^{-},\lambda_{2}^{-},\cdots ,\lambda_{{\tilde{m}}^{-}}^{-}],\;{\hat{\lambda }}^{-}=[{\hat{\lambda }}_{1}^{-},{\hat{\lambda }}_{2}^{-},\cdots ,{\hat{\lambda }}_{{\tilde{m}}^{+}}^{-}],$$ (61)

$$Q^{-}=p^{-}\left[\begin{array}{cc} \Psi ({\tilde{X}}^{-},{\tilde{X}}^{-}) & -\Psi ({\tilde{X}}^{-},{\tilde{X}}^{+})\\ -\Psi ({\tilde{X}}^{+},{\tilde{X}}^{-}) & \Psi ({\tilde{X}}^{+},{\tilde{X}}^{+}) \end{array}\right],$$ (62)

$$F^{-}=-{\left[\begin{array}{l} diag\left(\Psi ({\tilde{X}}^{-},{\tilde{X}}^{-})\right)-{\left(2p^{-}v^{-}{\left({\bar{e}}^{-}\right)}^{T}\Psi ({\bar{X}}^{-},{\tilde{X}}^{-})\right)}^{T}\\ -diag\left(\Psi ({\tilde{X}}^{+},{\tilde{X}}^{+})\right)+{\left(2p^{-}v^{-}{\left({\bar{e}}^{-}\right)}^{T}\Psi ({\bar{X}}^{-},{\tilde{X}}^{+})\right)}^{T} \end{array}\right]}^{T},$$ (63)

$$V^{-}=\left[\begin{array}{l} c_{2}^{-}{\tilde{e}}^{-}\\ c_{3}^{-}{\tilde{e}}^{+} \end{array}\right],$$ (64)

$$E^{-}={\left[\begin{array}{c} {\tilde{e}}^{-}\\ -{\tilde{e}}^{+} \end{array}\right]}^{T},$$ (65)

$$A^{-}=\left[\begin{array}{cc} -{\left({\tilde{e}}^{-}\right)}^{T} & 0^{1\times {\tilde{m}}^{+}}\\ 0^{1\times {\tilde{m}}^{-}} & -{\left({\tilde{e}}^{+}\right)}^{T} \end{array}\right],B^{-}=-\left[\begin{array}{c} 1\\ 1 \end{array}\right],$$ (66)

$${\bar{e}}^{-}=[1,1,\cdots {,1]}^{T}\in {ℜ}^{{\bar{m}}^{-}\times 1}.$$ (67)

The following can be obtained from (35), (36), (42) and (43).

$$\begin{array}{l} -\tau c_{2}^{+}<\lambda_{i}^{+}<c_{2}^{+}\Leftrightarrow \alpha_{i}^{+}>0\;and\;\beta_{i}^{+}>0,\\ -\tau c_{3}^{+}<{\hat{\lambda }}_{j}^{+}<c_{3}^{+}\Leftrightarrow {\hat{\alpha }}_{j}^{+}>0\;and\;{\hat{\beta }}_{j}^{+}>0. \end{array}$$ (68)

According to (39), (40) and (68), (*R*^+^)^2^ and $({\hat{R}}^{+}{)}^{2}$ can be derived as

$$\begin{array}{l} (R^{+}{)}^{2}=\frac{1}{\left|S^{+}\right|}\sum_{i\in S^{+}}\|\varphi ({\tilde{X}}_{i}^{+})-C^{+}{\|}^{2},\\ ({\hat{R}}^{+}{)}^{2}=\frac{1}{\left|{\hat{S}}^{+}\right|}\sum_{j\in {\hat{S}}^{+}}\|\varphi ({\tilde{X}}_{j}^{-})-C^{+}{\|}^{2}, \end{array}$$ (69)

where $S^{+}=\{i|-\tau c_{2}^{+}<\lambda_{i}^{+}<c_{2}^{+},i=1,2,\cdots ,{\tilde{m}}^{+}\}$ and ${\hat{S}}^{+}=\{j|-\tau c_{3}^{+}<{\hat{\lambda }}_{j}^{+}<c_{3}^{+},j=1,2,\cdots ,{\tilde{m}}^{-}\}$. Similarly, the following can be obtained.

$$\begin{array}{l} {\left(R^{-}\right)}^{2}=\frac{1}{\left|S^{-}\right|}\sum_{j\in S^{-}}\|\varphi ({\tilde{X}}_{j}^{-})-C^{-}{\|}^{2},\\ ({\hat{R}}^{-}{)}^{2}=\frac{1}{\left|{\hat{S}}^{-}\right|}\sum_{i\in {\hat{S}}^{-}}\|\varphi ({\tilde{X}}_{i}^{+})-C^{-}{\|}^{2}, \end{array}$$ (70)

where $S^{-}=\{j|-\tau c_{2}^{-}<\lambda_{j}^{-}<c_{2}^{-},j=1,2,\cdots ,{\tilde{m}}^{-}\}$ and ${\hat{S}}^{-}=\{i|-\tau c_{3}^{-}<{\hat{\lambda }}_{i}^{-}<c_{3}^{-},i=1,2,\cdots ,{\tilde{m}}^{+}\}$.

## 5. Experiments and results analysis

In order to test the performance of the proposed classification model, QHSVM, *ρ*-QHSVM, SVM, Pin-SVM, TWSVM and THSVM are compared by using artificial and benchmark datasets with noise. Moreover, *ρ*-QHSVM is used to classify strip steel surface defects datasets obtained from a steel plant in China. It must be noted that THSVM is an extended binary classifier based on SVDD_neg. In this experiment, the nonlinear classifiers adopt kernel function *Ψ*(***X***_*i*_,***X***_*l*_) = exp(−‖***X***_*i*_−***X***_*l*_‖^2^/2*δ*^2^). And the linear classifiers adopt *Ψ*(***X***_*i*_,***X***_*l*_) =***X***_*i*_⋅***X***_*l*_. All classifiers are solved and executed with MATLAB 7.11 on Windows 7 running on a PC with Intel Core CPU (3.2 GHz) and 4 GB RAM.

Moreover, for a fair comparison, all classifiers use the same quadprog solver in MATLAB. For QHSVM, some parameters need to be determined. In order to reduce the computation complexity, assume that $c_{2}^{+}=c_{2}^{-}$, $c_{3}^{+}=c_{3}^{-}$ and *v*^+^ = *v*^−^ for QHSVM and *ρ*-QHSVM. This brevity method has also been used in [16], [22], [23], [29] and [32]. For TWSVM and THSVM, *c*_1_ = *c*_2_ and *v*_1_ = *v*_2_ are set. All parameters *c*'*s*, *v*'*s* and *δ*'*s* are chosen from the set {2^*l*^|*l* = −9,−8,⋯,10}. *K* is used to control the number of nearest neighbors. For the nearest neighbors’ algorithm, *K* is generally determined by grid search. In [18] and [32], *K* has been discussed. According to [18] and [32], *K* is set as 8. The parameter *τ* is chosen from {0.1,0.2,0.5,1}. There are some common parameter selection methods: exhaustive search, 5-fold cross validation, grid search and optimization search. In the experiments, in order to completely cut interactions between training and testing phases, the following selection methods are adapted. Firstly, we randomly split *m*_*all*_ samples into *m*_*training*_ training samples and *m*_*testing*_ testing samples, where *m*_*all*_ = *m*_*training*_+*m*_*testing*_. And the split step is repeated *n*_*training*_ times. Thus, *n*_*training*_ training/testing datasets are obtained. Then, the parameter values are determined by 5-fold cross validation and grid search for the *i*-th training dataset, where *i* = 1,2,⋯,*n*_*training*_. The final classifier is set through the determined parameter values and is used to evaluate the accuracy for the *i*-th testing dataset. It can be seen that the step is repeated *n*_*training*_ times. Finally, we can obtain *n*_*training*_ testing accuracies. And the average accuracy and the standard deviation of all accuracies are calculated. The average accuracy and the standard deviation are used to evaluate the performance of the classifiers in UCI datasets and strip steel surface defects datasets. In artificial datasets, the average accuracy is used to represent the performance of the classifiers. To make statistical analysis sound, *n*_*training*_ is set as 50 and *m*_*training*_ = 5*m*_*testing*_.

### 5.1 Artificial datasets

To illustrate the ability of QHSVM graphically, the 2-D artificial datasets with Gaussian distribution are adopted. Suppose the samples $X_{i}^{+}$ (*i* = 1,2,⋯,*m*^+^) satisfy Gaussian distribution *N*(*μ*_1_,∑_1_). And the mean *μ*_1_ is [−0.38,−0.38]^*T*^ and covariance matrix ∑_1_ is *diag*(0.1,0.1). Suppose the samples $X_{j}^{-}$ (*j* = 1,2,⋯,*m*^−^) also satisfy Gaussian distribution *N*(*μ*_2_,∑_2_) with *μ*_2_ = [0.38,0.38]^*T*^ and ∑_2_ = *diag*(0.03,0.03). Moreover, some samples in artificial datasets are introduced with noise around the decision boundary by using an adjustable parameter *θ*, which are called noise samples. *θ* is the ratio of the number of noise samples to the number of training samples. These noise samples affect the labels around the boundary. The labels of these noise samples are selected from {+1,−1} with equal probability. And the positions of these samples satisfy Gaussian distribution with the following parameters *μ*_*n*_ = [0,0]^*T*^ and Σ_*n*_ = *diag*(0.03,0.03).

**Fig 4:**
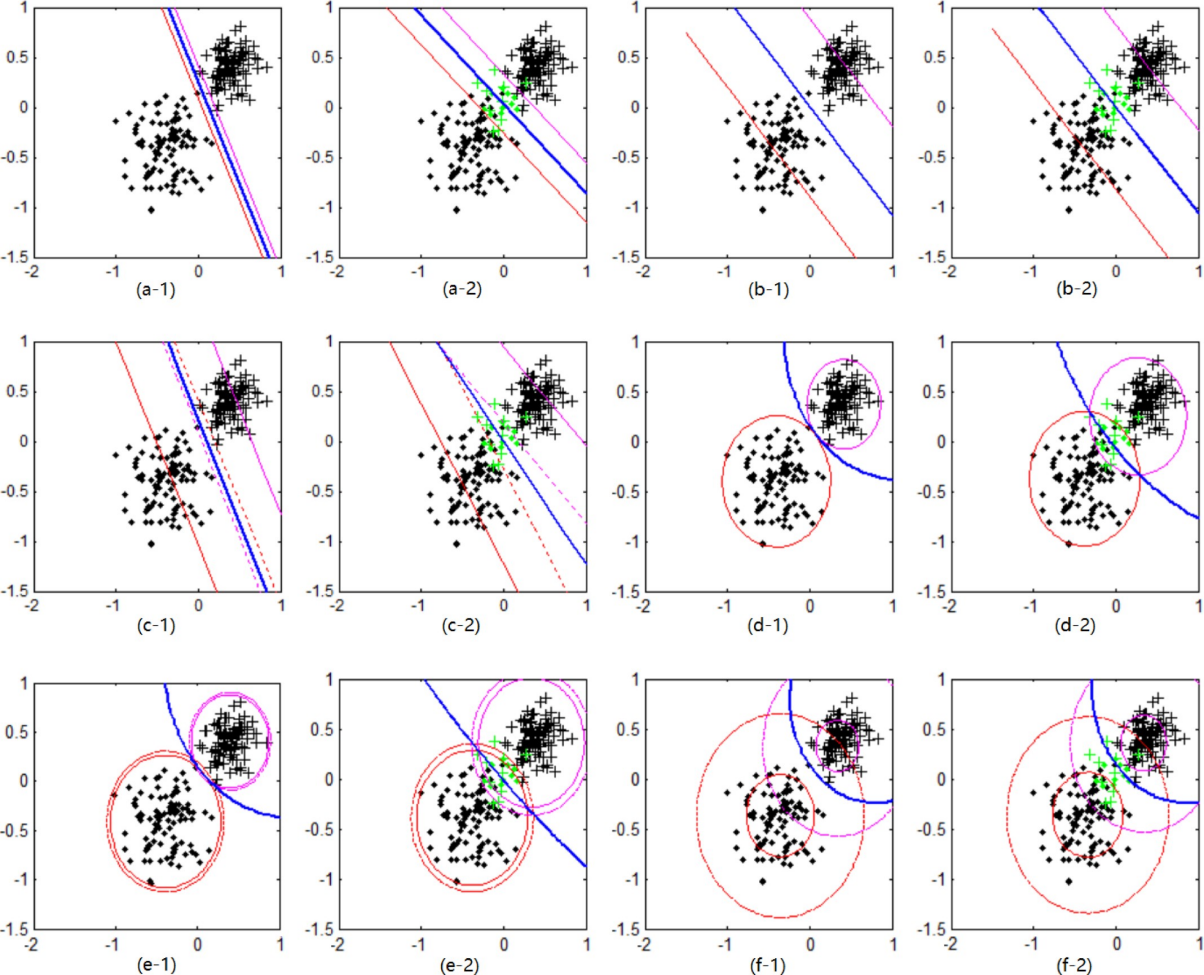
The training results for six classifiers with linear kernel. (a-*i*) SVM, (b-*i*) Pin-SVM, (c-*i*) TWSVM, (d-*i*) THSVM, (e-*i*) QHSVM (*τ* = 0) and (f-*i*) QHSVM (*τ* = 0.5), where *i* = 1, 2. (a-1) ‒ (f-1) for the dataset *D*_1_ and (a-2) ‒ (f-2) for the dataset $D_{1}^{n}$. The decision boundaries (blue thick solid curves), support hyper-planes or hyper-spheres (magenta and red thin solid curves) and the hyper-planes paralleling to support hyper-planes or the hyper-spheres having the same center of support hyper-spheres (magenta and red thin dashed curves). Two classes of samples (“**·**” and “**+**” in black) and noise samples (“**·**” and “**+**” in green).

Firstly, the dataset *D*_1_ with *m*^+^ = 100 and *m*^**−**^ = 100 is built according to the above Gaussian distribution. Then, the dataset $D_{1}^{n}$ is obtained by introducing noise samples with *θ* = 10% into *D*_1_. **Fig 4** shows the training results on the datasets *D*_1_ and $D_{1}^{n}$ for SVM, Pin-SVM, TWSVM, THSVM, QHSVM (*τ* = 0) and QHSVM (*τ* = 0.5) with linear kernel. It can be seen from **Fig 4(A-1)** that the decision boundary of SVM is obtained based on two parallel support hyper-planes. These two support hyper-planes belong to boundary hyper-planes. Compared with **Fig 4(A-1)**, the boundary hyper-planes of SVM in **Fig 4(A-2)** change in position. The result proves that SVM is adversely affected by noise samples. The support hyper-planes of Pin-SVM are quantile hyper-planes. Many samples are added between two quantile hyper-planes, which dilutes the adverse impact of noise samples. So, the decision boundary has not changed much for Pin-SVM on *D*_1_ and $D_{1}^{n}$. Different from SVM, TWSVM uses two nonparallel support hyper-planes to describe two classes of samples. This attribute makes TWSVM be in favor of the description of training dataset, especially the dataset with cross planes. However, each support hyper-plane of TWSVM needs to be supported by a parallel boundary hyper-plane. So, noise samples also affect the nonparallel support hyper-planes of TWSVM. THSVM builds two support hyper-spheres. Each hyper-sphere covers one class of samples and keeps away from the other class of samples. THSVM maximizes the margin between the two classes and the inner-class clustering of samples. So, the decision boundary of THSVM becomes more reasonable. It can be seen from **Fig 4(D-1)** that the decision boundary of THSVM curves to the clustering samples. However, its two support hyper-spheres belonging to boundary are affected by noise samples near the boundary. If *τ* = 0, the quantile hyper-spheres reduce to the boundary hyper-spheres for QHSVM. So, QHSVM (*τ* = 0) includes two boundary hyper-spheres with the same center for every class of samples. It can be seen that it has similar attributes with THSVM. So, it is clear that the decision boundary of QHSVM (*τ* = 0) will be changed by noise samples. QHSVM (*τ* = 0.5) builds two quantile hyper-spheres with the same center for every class of samples. Compared with the boundary hyper-spheres, some samples are added inside or outside of the quantile hyper-spheres. These samples reduce the adverse impact caused by noise samples around the decision boundary. So, the training results of QHSVM (*τ* = 0.5) for *D*_1_ and $D_{1}^{n}$ are not changed obviously, just like the support hyper-spheres and the decision boundary. Moreover, the decision boundary of QHSVM (*τ* = 0.5) for *D*_1_ and $D_{1}^{n}$ are both reasonable, which are curved to clustering samples. All these results prove that QHSVM has better performance because it integrates the excellent attributes of Pin-SVM, TWSVM and THSVM.

Then, the dataset *D*_2_ with *m*^+^ = 200 and *m*^−^ = 200 is built. According to the prescribed rules, it is divided into the training dataset and testing dataset. And noise samples with *θ* = 0%, 5%, 10%, 20% are introduced into the training dataset respectively. At last, the testing accuracies for different classifiers with linear kernel are shown in **Table 1**. For *θ* = 0%, compared with SVM and TWSVM, THSVM and QHSVM have better classification accuracies, which shows that the nonparallel hyper-planes (hyper-spheres) and inner-class clustering of samples strengthen the performance of classifiers. For *θ* = 0%, the testing accuracy of Pin-SVM is lower than that of SVM. One possible reason is that there are some isolated samples in *D*_2_, which can be seen from **Fig 4(B-1)**. The only error point in **Fig 4(B-1)** deviates from the dataset with “+” in black. Quantile hyper-plane is sensitive to isolated samples as well as noise samples. For *θ*≠0%, QHSVM provides the best testing accuracy compared with the other classifiers. All these results show that QHSVM performs the best in accuracy for datasets with noise samples, which is due to pinball losses, two nonparallel support hyper-spheres and inner-class clustering of samples. For *θ*≠0%, the testing accuracy of Pin-SVM is higher than that of SVM, TWSVM and THSVM, which shows that the pinball loss can improve classifier's performance for datasets with noise samples. The testing accuracy of Pin-SVM is lower than that of QHSVM. The reason is that it does not have the attributes of inner-class clustering of samples and nonparallel support hyper-planes. Testing accuracies corresponding to different classifiers with nonlinear kernel are shown in **Table 2**. For all conditions, QHSVM has the best testing accuracy. Compared with **Table 1**, testing accuracies corresponding to all classifiers in **Table 2** are improved, which shows that the classifiers with nonlinear kernel improves the classification results.

**Table 1:** The testing accuracies for linear classifiers on datasets with noise.

*θ*	SVM	Pin-SVM	TWSVM	THSVM	QHSVM
	Accuracy (%)	Accuracy (%)	Accuracy (%)	Accuracy (%)	Accuracy (%)
0%	97.41	97.31	97.41	**97.51**	**97.51**
5%	96.87	97.17	96.92	97.02	**97.26**
10%	96.18	96.57	96.18	96.13	**96.85**
20%	94.5	95.49	94.89	95.04	**95.78**

**Table 2:** The testing accuracies for nonlinear classifiers on datasets with noise.

*θ*	SVM	Pin-SVM	TWSVM	THSVM	QHSVM
	Accuracy (%)	Accuracy (%)	Accuracy (%)	Accuracy (%)	Accuracy (%)
0%	97.96	97.91	97.86	97.86	**98.05**
5%	97.46	97.71	97.46	97.56	**97.86**
10%	96.82	97.12	96.92	96.97	**97.46**
20%	95.29	96.03	95.49	95.73	**96.62**

Finally, in order to test the performance of *ρ*-QHSVM, the datasets *D*_3_ (*m*^+^ = *m*^−^ = 100), *D*_4_ (*m*^+^ = *m*^−^ = 400), *D*_5_ (*m*^+^ = *m*^−^ = 700) and *D*_6_ (*m*^+^ = *m*^−^ = 1000) are built. And noise samples with *θ* = 0% are introduced into these datasets. Nonlinear classifiers of SVM, Pin-SVM, TWSVM, THSVM and QHSVM are tested on accuracy and speed. Testing results are shown in **Table 3**. The conclusions on **Table 3** are nearly the same with that on **Table 2**, which shows that QHSVM has excellent and stable performance for different-scale datasets. THSVM and TWSVM are faster than SVM, Pin-SVM and QHSVM. The reason is that these two classifiers solve two smaller QPPs instead of one large QPP used for SVM and Pin-SVM. The efficiency of QHSVM is the lowest because it solves two large QPPs to obtain better classification accuracy. So, QHSVM is not fit for high efficiency requirement. In order to solve the above problem, *ρ*-QHSVM with adjustable execution speed is proposed. It uses parameter *ε* to adjust the execution speed. The accuracy and execution time of *ρ*-QHSVM with different *ε* for different-scale datasets are shown in **Table 3**. The classification accuracy of *ρ*-QHSVM reduces as *ε* becomes small. The sparseness of surrounding samples and clustering samples is controlled by *ε*. This is caused by the fact that reducing *ε* means reducing the number of surrounding samples. Fewer surrounding samples inevitably reduce the classification accuracy for datasets with noise samples. For small-scale datasets, if *ε* is big, *ρ*-QHSVM is close to QHSVM, and exceeds the other classifiers in accuracy. Take the dataset *D*_3_ as an example, when *ε* = 0.7, *ρ*-QHSVM is close to QHSVM in accuracy. For large-scale datasets, when *ε* is small, *ρ*-QHSVM is close to QHSVM in accuracy. And it also exceeds the other classifiers in accuracy. For the dataset *D*_6_, the classification accuracy of *ρ*-QHSVM exceeds that of Pin-SVM when *ε* = 0.3, and is close to that of QHSVM when *ε* = 0.4. It can be seen from **Table 3** that the smaller *ε* is, the higher the efficiency of *ρ*-QHSVM is. When *ε*≤0.4, *ρ*-QHSVM is the fastest classifier, which shows that the efficiency of *ρ*-QHSVM can be adjusted by *ε*. The results of **Table 3** show that the improvement of execution time brought by *ρ*-QHSVM is limited for small-scale datasets under the premise of high accuracy. However, for small-scale datasets, this difference is insignificant because the execution time of classifiers is small. For large-scale datasets, the execution time of *ρ*-QHSVM is reduced greatly under the premise of high accuracy. For example, *ρ*-QHSVM has high efficiency and testing accuracy for the dataset *D*_6_ when *ε* = 0.3. So, *ρ*-QHSVM is fit for large-scale datasets with high efficiency requirement.

**Table 3:** The testing accuracies and execution time for six classifiers on different-scale datasets with noise.

Classifiers	*ε*	Performance	*D*_3_ (*m*^+^ = *m*^−^ = 100)	*D*_4_ (*m*^+^ = *m*^−^ = 400)	*D*_5_ (*m*^+^ = *m*^−^ = 700)	*D*_6_ (*m*^+^ = *m*^−^ = 1000)
SVM	—	Accuracy (%)	96.87	96.52	97.13	97.03
		Time (s)	0.729	36.41	174.4	344.6
Pin-SVM	—	Accuracy (%)	97.17	96.92	97.6	97.36
		Time (s)	0.666	39.07	211	367.3
TWSVM	—	Accuracy (%)	96.67	96.6	97.06	97.07
		Time (s)	0.235	13.08	57.23	113.6
THSWM	—	Accuracy (%)	96.87	96.74	97.26	97.19
		Time (s)	0.292	14.1	56.08	127.7
QHSVM	—	Accuracy (%)	**97.36**	97.31	97.79	97.61
		Time (s)	1.226	62.74	334	719.8
*ρ*-QHSVM	0.7	Accuracy (%)	97.26	**97.41**	97.71	97.61
		Time (s)	0.877	42.67	111.8	345.3
	0.6	Accuracy (%)	96.97	97.31	**97.81**	**97.66**
		Time (s)	0.548	33.27	93.06	212.1
	0.5	Accuracy (%)	96.74	97.07	97.71	97.56
		Time (s)	0.25	14.47	60.57	122.9
	0.4	Accuracy (%)	94.3	96.87	97.61	97.61
		Time (s)	0.2	11.35	42.59	92.9
	0.3	Accuracy (%)	94.3	96.72	97.06	97.56
		Time (s)	0.102	5.837	17.42	52.72

### 5.2 UCI datasets with noise samples

In order to further test the performance of QHSVM, all classifiers are run on fifteen public benchmark datasets downloaded from the UCI Machine Learning Repository [33]. Ten small-scale or middle-scale datasets are used for testing accuracy, including Heart, Ionosphere, Breast, Thyroid, Australian, WPBC, Pima, German, Sonar and ILPD. And five large-scale datasets are used for testing accuracy and speed, including Wifi, Splice, Wilt, Musk and Spambase. The details of these original benchmark datasets are listed in **Table 4**. In order to highlight the anti-noise ability of QHSVM, the benchmark datasets with noise samples are tested. Each benchmark dataset is corrupted by zero-mean Gaussian noise. For each feature, the ratio of the variance of noise to that of the feature denoted as *θ* is set to be 0%, 5% and 10%. And all original and corrupted benchmark datasets are normalized before training.

**Table 4:** The details of fifteen benchmark datasets.

Datasets	Number of samples	Dimension	Datasets	Number of samples	Dimension
Heart	270	13	Wifi	2000	6
Ionosphere	351	34	Splice	3190	60
Breast	569	30	Wilt	4889	5
Thyroid	215	5	Musk	6598	166
Australian	690	14	Spambase	4601	57
WPBC	198	34			
Pima	768	8			
German	1000	20			
Sonar	208	60			
ILPD	583	10			

**Table 5** shows the testing accuracies of SVM, Pin-SVM, TWSVM, THSVM and QHSVM with nonlinear kernels on the ten benchmark datasets. It can be seen that QHSVM achieves the best testing accuracy for majority of datasets. For the original benchmarked datasets with *θ* = 0%, QHSVM and THSVM yield the best testing accuracy on 5 and 2 of 10 datasets respectively. And SVM, Pin-SVM and TWSVM yield the best testing accuracy on 1, 1 and 1 of 10 datasets respectively. This result shows that QHSVM and THSVM with nonparallel hyper-spheres and inner-class clustering of samples strengthen the performance of classifiers. It should be pointed out that QHSVM has obvious advantage for corrupted benchmark datasets. For the corrupted benchmark datasets with *θ* = 5% and *θ* = 10%, QHSVM and Pin-SVM yield the best testing accuracies on 13 and 5 of 20 datasets respectively. And SVM, TWSVM and THSVM yield the best testing accuracy on 2, 1 and 1 of 20 datasets respectively. This result shows that QHSVM and Pin-SVM are better than classifiers with hinge loss for the corrupted datasets. Moreover, for the original and corrupted benchmark datasets, QHSVM is superior to THSVM and Pin-SVM, because it has merits of pinball losses, nonparallel hyper-spheres and inner-class clustering of samples. This conclusion is the same as experimental results on the artificial datasets.

**Table 5:** The testing accuracies of five classifiers on ten benchmark datasets.

Datasets	SVM	Pin-SVM	TWSVM	THSVM	QHSVM
	Accuracy (%)	Accuracy (%)	Accuracy (%)	Accuracy (%)	Accuracy (%)
Heart (*θ* = 0%)	81.58±3.91	81.30±4.65	80.85±5.59	**82.46**±4.22	81.30±4.14
*θ* = 5%	79.85±3.90	79.92±3.88	79.72±5.24	79.91±4.00	**80.01**±3.67
*θ* = 10%	**78.10**±3.50	**78.10**±2.76	77.84±3.81	77.90±3.06	78.04±2.76
Ionosphere (*θ* = 0%)	91.30±4.10	91.44±3.26	89.94±3.97	91.68±4.02	**92.12**±3.78
*θ* = 5%	90.27±4.59	90.97±3.59	89.46±3.82	**91.59**±4.14	91.52±3.78
*θ* = 10%	89.33±4.65	90.03±3.51	88.19±4.38	89.33±3.55	**90.41**±2.89
Breast (*θ* = 0%)	**94.29**±1.22	93.67±1.53	93.82±2.02	93.46±2.12	93.55±1.38
*θ* = 5%	93.06±2.11	93.05±1.72	92.96±2.76	92.73±2.80	**93.14**±2.28
*θ* = 10%	92.61±2.20	**93.05**±2.16	92.61±2.71	92.47±2.88	93.00±2.35
Thyroid (*θ* = 0%)	93.43±3.79	93.58±2.43	93.68±2.79	94.45±2.89	**94.49**±2.56
*θ* = 5%	93.28±3.28	93.42±2.95	**93.60**±3.65	93.17±3.83	93.50±2.91
*θ* = 10%	91.76±3.50	92.18±3.20	91.72±3.79	92.04±4.14	**92.39**±2.70
Australian (*θ* = 0%)	74.28±3.48	74.42±6.18	**80.02**±2.23	79.91±4.23	79.91±2.24
*θ* = 5%	79.63±4.75	79.63±3.64	79.68±2.47	79.36±4.27	**79.75**±2.38
*θ* = 10%	**79.79**±6.15	79.56±3.06	78.08±3.77	78.25±3.36	**79.79**±3.02
WPBC (*θ* = 0%)	79.92±7.52	79.66±4.78	80.12±6.23	80.04±5.04	**80.20**±4.97
*θ* = 5%	78.86±7.21	79.52±4.43	79.37±5.36	79.74±5.36	**79.94**±4.71
*θ* = 10%	77.70±6.69	77.96±4.29	77.80±5.49	77.77±5.13	**77.97**±4.05
Pima (*θ* = 0%)	73.55±4.56	72.80±4.59	71.41±4.98	72.80±4.47	**73.99**±3.79
*θ* = 5%	71.43±5.29	73.02±4.15	71.25±5.08	71.44±5.11	**73.57**±3.92
*θ* = 10%	70.92±4.47	**72.61**±4.04	70.95±4.08	70.84±4.89	72.47±4.27
German (*θ* = 0%)	72.56±2.63	71.17±2.56	71.59±3.20	**72.76**±2.95	71.59±2.76
*θ* = 5%	71.08±3.69	71.08±2.83	71.02±3.53	71.20±2.75	**71.56**±2.76
*θ* = 10%	70.89±3.27	71.02±2.37	70.65±3.85	70.90±3.08	**71.46**±2.80
Sonar (*θ* = 0%)	84.63±4.27	85.62±4.39	85.10±4.69	85.26±4.05	**85.92**±4.78
*θ* = 5%	81.66±4.02	**83.38**±4.33	81.85±4.47	82.12±3.96	83.27±4.52
*θ* = 10%	80.24±3.78	80.69±5.15	79.95±3.67	80.73±4.09	**80.79**±3.91
ILPD (*θ* = 0%)	86.46±3.81	**87.12**±1.59	86.81±3.17	86.47±3.02	87.05±1.96
*θ* = 5%	86.20±3.30	86.91±2.75	86.47±3.67	85.94±2.83	**87.12**±2.28
*θ* = 10%	86.12±3.16	**86.37**±2.52	85.99±3.35	85.94±3.18	86.12±2.94

**Table 6** shows the classification accuracies and execution time of SVM, Pin-SVM, TWSVM, THSVM and *ρ*-QHSVM with nonlinear kernels on the five large-scale datasets. In the above section, it has been found that *ρ*-QHSVM improves the execution efficiency for the large-scale artificial datasets under the premise of high accuracy. This part of experiment also proves the same conclusion. According to the experimental results on the artificial datasets, parameter *ε* of *ρ*-QHSVM is set as 0.3. Compared with the other classifiers, the execution time of *ρ*-QHSVM is the shortest. The reason is that *ρ*-QHSVM solves smaller QPPs with inequality constraints. These smaller QPPs are produced on sparse surrounding samples. On the other hand, *ρ*-QHSVM achieves the best testing accuracy for the majority of datasets. For the original benchmark datasets with *θ* = 0%, *ρ*-QHSVM yields the best testing accuracy on 3 of 5 datasets, while for the corrupted benchmark datasets with *θ* = 5% and *θ* = 10%, *ρ*-QHSVM yields the best accuracy on 6 of 10 datasets. The reason is that the local center-based density estimation method ensures the reasonable division about surrounding samples and clustering samples. In general, *ρ*-QHSVM has higher efficiency and accuracy for large-scale datasets compared with the other classifiers.

**Table 6:** The testing accuracies and execute time of five classifiers on five large-scale datasets.

Datasets	SVM	Pin-SVM	TWSVM	THSVM	*ρ*-QHSVM
	Accuracy (%) Time (s)	Accuracy (%) Time (s)	Accuracy (%) Time (s)	Accuracy (%) Time (s)	Accuracy (%) Time (s)
Wifi (*θ* = 0%)	74.47±4.30	74.60±3.32	74.72±4.44	74.93±4.24	**75.07**±3.55
	377.5	450.1	121.3	127	97.02
*θ* = 5%	73.59±4.97	74.38±4.55	73.73±5.08	74.04±5.25	**74.84**±3.57
	372.6	465.5	130	128	94.96
*θ* = 10%	72.38±5.12	73.94±4.10	73.16±3.79	73.61±4.64	**74.55**±3.82
	364.3	474.3	127.2	117.2	92.94
Splice (*θ* = 0%)	86.92±2.09	87.34±2.09	87.29±2.48	87.29±2.28	**87.40**±1.95
	787.9	728.2	238.7	263.2	178.3
*θ* = 5%	86.21±2.99	86.44±2.51	86.48±3.26	86.08±3.65	**86.58**±2.77
	803	749.2	257	267.4	186
*θ* = 10%	84.26±2.97	**85.50**±2.57	84.79±3.14	85.09±2.94	85.42±2.77
	811.7	761.6	269	274.3	195.3
Wilt (*θ* = 0%)	83.82±1.03	84.10±1.08	84.16±0.90	**84.90**±0.93	84.66±0.84
	818.2	904	267.3	250.7	206.2
*θ* = 5%	83.19±2.18	84.28±0.99	83.44±1.09	**84.39**±0.93	84.32±0.77
	846	900.5	259.7	258.2	211.1
*θ* = 10%	83.60±1.41	84.32±0.95	83.09±1.00	83.58±1.00	**84.52**±1.03
	787.4	887.1	264.6	255.3	207
Musk (*θ* = 0%)	95.72±2.81	95.11±1.81	94.67±2.43	95.51±1.89	**95.96**±2.30
	2029	2194	751.7	755.7	520.9
*θ* = 5%	94.51±2.61	**94.91**±2.11	94.20±2.51	94.67±2.02	94.89±2.23
	2081	2030	746.1	756.7	534.5
*θ* = 10%	93.94±1.78	93.82±2.38	94.06±1.95	94.03±1.95	**94.89**±2.29
	2114	2111	700.6	761.6	548.4
Spambase (*θ* = 0%)	**88.94**±3.76	88.76±3.03	87.03±3.09	88.24±5.11	88.78±3.51
	1082	1069	345.8	367.6	261.2
*θ* = 5%	87.30±3.65	87.77±3.04	86.21±3.59	86.47±4.57	**88.40**±3.57
	1094	1080	354.3	373.6	255.5
*θ* = 10%	86.56±3.49	**86.75**±3.79	85.57±4.10	85.39±3.65	86.56±3.63
	1062	1091	354.3	364.6	264.5

### 5.3 PASCAL VOC dataset

**Fig 5:**
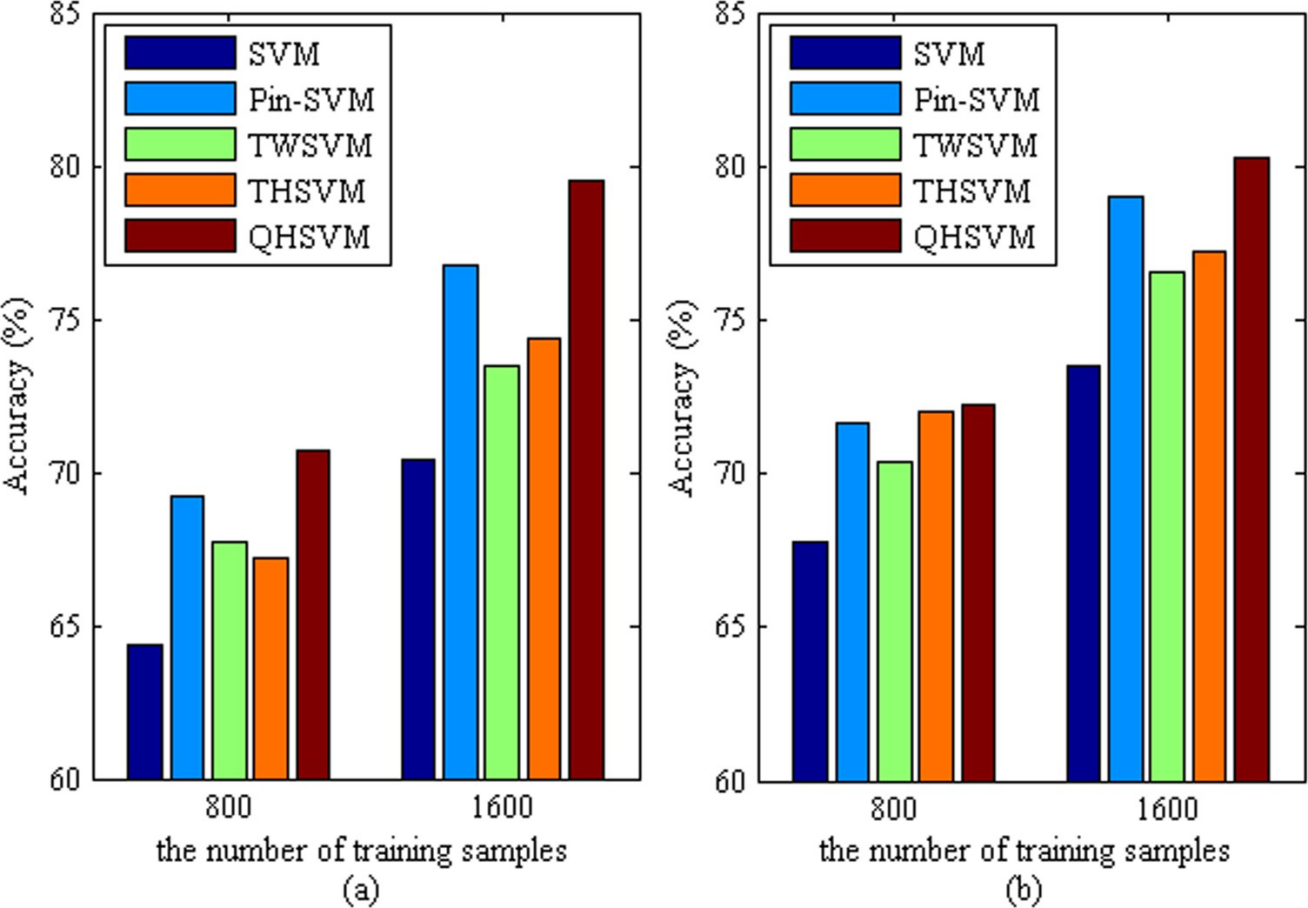
The mean accuracies of five classifiers in two datasets. (a) the PASCAL VOC dataset with noise, (b) the original PASCAL VOC dataset.

The PASCAL VOC dataset [34] is a public benchmark dataset and is often used in challenge competitions for supervised machine learning. The dataset is composed of color images of twenty visual object classes in realistic scenes. In the experiment, the ten classes of them are chosen, such as person, cat, cow, dog, horse, sheep, bicycle, bus, car and motorbike. These color images are converted to the intensity images, then are resized to s times the size of the original color images so that they have the specified 4096 pixels, where s is a positive real number. So, each image is represented as a sample vector with 4096 elements. We choose 800 and 1600 vectors as training samples respectively and the others are testing samples. In order to highlight the anti-noise ability, the PASCAL VOC dataset with noise are built. The PASCAL VOC dataset is corrupted by zero-mean Gaussian noise. For each feature, the ratio of the variance of noise to that of the feature denoted as *θ* is set to be 5%. For brevity, we build ten nonlinear binary classifiers with one-against-rest method. Then, the mean of ten accuracies is presented in **Fig 5**.

It can be seen from **Fig 5(A)** that the performance of our QHSVM is superior to that of SVM, Pin-SAVM, TWSVM and THSVM in challenging the PASCAL VOC dataset with noise. The result highlights that the new attribute of pinball losses improves the anti-noise ability of our QHSVM. Furthermore, the attribute of nonparallel hyper-spheres strengthens the generalization performance of the classifier. **Fig 5(B)** shows that the average accuracy of QHSVM is not lower than that of other classifiers. This indicates that the QHSVM also has reliable performance in challenging the original PASCAL VOC dataset. The robustness of QHSVM is strengthened by maximizing the margin between two hyper-spheres with the same center and maximizing the inner-class clustering of samples. Moreover, two nonparallel quantile hyper-spheres improve the generalization of QHSVM. In addition, the performance of all classifiers is improved with the increase of training samples. In the case of more training samples, the performance of all classifiers in corrupted dataset is close to that in original dataset. Notably, the accuracies of our classifier in the two datasets are close. This also shows that our QHSVM has better robustness than other classifiers.

### 5.4 Strip steel surface defects datasets

**Fig 6:**
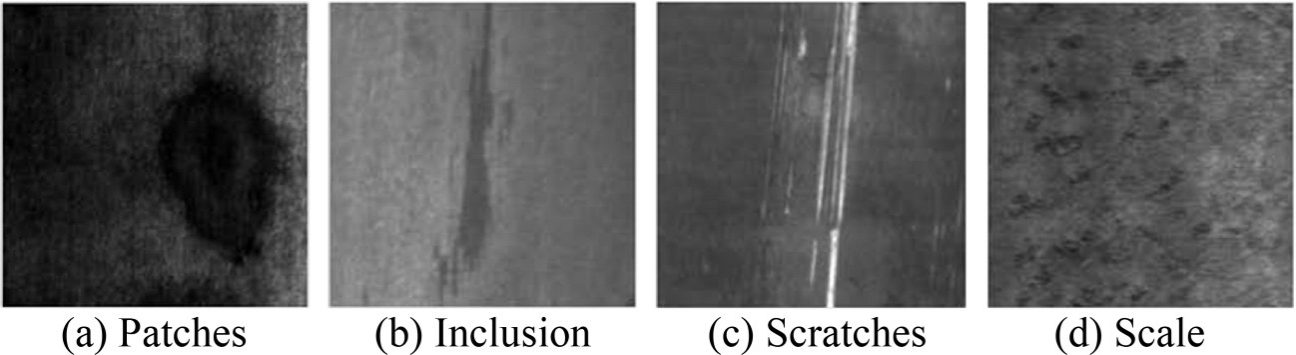
Four types of defects for strip steel surface.

Strip steel surface defects datasets are obtained from Northeastern University (NEU) surface database [35]. In the experiment, four typical defects datasets in NEU surface database are investigated: patches (S1 Dataset), inclusion (S2 Dataset), scratches (S3 Dataset) and scale (S4 Dataset). Their typical images are shown in **Fig 6**. These defect images are extracted as defect samples, and each defect sample includes sixteen attributes. This means that each defect sample is a 16-dimensional vector. Their related attributes have been described in our previous work [36]. It can be seen that the strip steel surface defects classification belongs to multi-class classification. There are many multi-class classification methods based on binary classifier, such as one-against-one, one-against-rest, decision directed acyclic graph and binary tree [37]. And the binary tree model is most widely used. Multi-class classifiers for SVM, Pin-SVM, TWSVM, THSVM and *ρ*-QHSVM can be obtained on the binary tree. According to the binary tree model, three QPPs are needed to solve for SVM and Pin-SVM, while six QPPs are needed to solve for TWSVM, THSVM and *ρ*-QHSVM.

**Fig 7:**
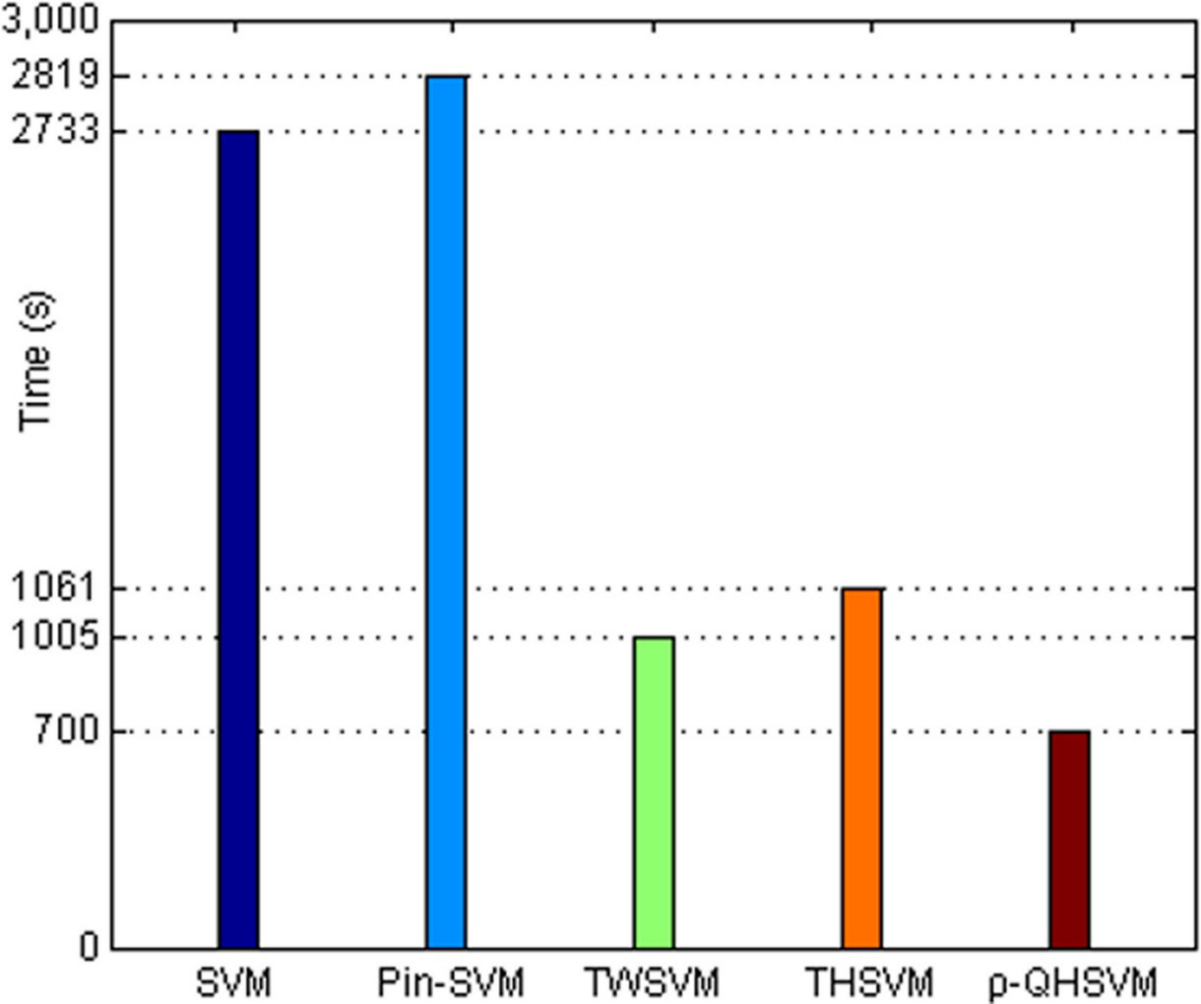
Execution time of five classifiers for strip steel surface defects datasets.

Moreover, to obtain more samples, the strip steel surface defects datasets are supplemented by rotation, distortion, translation, and scaling. In the end, the strip steel surface defects datasets include 8000 samples and each type of defects includes 2000 samples. All parameters of classifiers are obtained with the same method mentioned above. And the parameter *ε* of *ρ*-QHSVM is set to 0.3. The accuracies and execution time of all classifiers for all types of defects are shown in **Table 7** and **Fig 7** respectively. It can be seen from **Table 7** that the accuracy of *ρ*-QHSVM is always the best for all types of defects. The accuracy of Pin-SVM is better than that of SVM, TWSVM and THSVM. The reason is that the strip steel surface defects datasets are corrupted by noise usually. It is well known that there is noise on the production line of strip steel. So, the pinball losses in *ρ*-QHSVM and Pin-SVM work for the strip steel surface defects datasets with noise samples. Moreover, the other excellent attributes improve the performance of *ρ*-QHSVM further. Besides, the efficiency of *ρ*-QHSVM is high. TWSVM, THSVM and *ρ*-QHSVM is better than SVM and Pin-SVM in execution time, which is shown in **Fig 7**. Though SVM and Pin-SVM only need to solve three QPPs for four types of datasets, these QPPs are all large. TWSVM, THSVM and *ρ*-QHSVM need to solve smaller QPPs, which improves the execution time. *ρ*-QHSVM has the fastest speed, which is benefited from the local center-based density estimation method. The method improves the classification efficiency under the premise of high accuracy. In summary, *ρ*-QHSVM is very fit for the strip steel surface defects classification.

**Table 7:** The accuracies of five classifiers for all types of defects.

Defects	SVM	Pin-SVM	TWSVM	THSVM	*ρ*-QHSVM
Patches	91.88±2.93	92.87±2.33	91.94±2.26	92.30±2.05	**94.00**±2.39
Inclusion	92.17±2.01	95.21±2.07	93.15±2.40	94.78±3.82	**95.61**±2.53
Scratches	88.13±3.42	88.51±3.06	87.85±2.76	87.83±4.13	**89.70**±2.99
Scale	84.23±2.53	88.46±2.20	85.97±2.93	86.54±3.11	**88.96**±2.70

## 6. Conclusions

A novel QHSVM classifier is proposed for pattern recognition in this paper. QHSVM has remarkable attributes: pinball losses, two nonparallel quantile hyper-spheres and inner-class clustering of samples. The quantile hyper-spheres ensure that QHSVM is insensitive to noise, especially the feature noise around the decision boundary. The robustness of QHSVM algorithm is strengthened by maximizing the margin between two hyper-spheres with the same center and maximizing the inner-class clustering of samples. Moreover, compared with standard SVM model, two nonparallel quantile hyper-spheres improve the generalization of QHSVM. On the other hand, in order to satisfy the requirement of high efficiency for large-scale datasets classification, a new version of QHSVM with adjustable execution speed is proposed, which is called *ρ*-QHSVM. Under the premise of high accuracy, *ρ*-QHSVM reduces the execution time. That benefits from the local center-based density estimation which reasonably divides training samples into surrounding samples and clustering samples. The proposed QHSVM and *ρ*-QHSVM are compared with SVM, Pin-SVM, TWSVM and THSVM through numerical experiments on artificial, benchmark and strip steel surface defects datasets with noise. The results show that QHSVM performs the best in accuracy for datasets with noise samples, which is due to pinball losses, two nonparallel support hyper-spheres and inner-class clustering of samples. The execution time of ρ-QHSVM is reduced greatly under the premise of high accuracy for large-scale datasets, especially strip steel surface defects datasets. *ρ*-QHSVM has the fastest speed, which is benefited from the local center-based density estimation method. In the future, it is necessary to find the optimal parameters for QHSVM with some effective methods. And how to apply QHSVM to unbalanced datasets will be investigated.

## Supporting information

S1 Dataset Patches dataset. The first typical strip steel surface defects dataset.
(ZIP)

S2 Dataset Inclusion dataset. The second typical strip steel surface defects dataset.
(ZIP)

S3 Dataset Scratches dataset. The third typical strip steel surface defects dataset.
(ZIP)

S4 Dataset Scale dataset. The fourth typical strip steel surface defects dataset.
(ZIP)

S1 Text Fifteen benchmark datasets. UCI datasets.
(TXT)
