# Supplementary figures and images for: Support vector machine with quantile hyper-spheres for pattern classification (part 2 of 6)
Source: PLoS One. 2019 Feb 15;14(2):e0212361. doi: 10.1371/journal.pone.0212361 (PMC6377146; doi:10.1371/journal.pone.0212361)

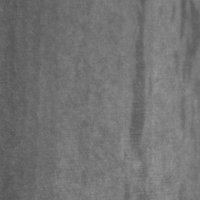

Supplement: S2 Dataset — The second typical strip steel surface defects dataset. (ZIP) [file pone.0212361.s002.zip › inclusion/In_19.bmp]

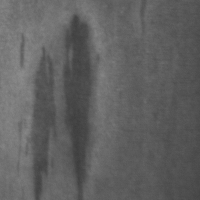

Supplement: S2 Dataset — The second typical strip steel surface defects dataset. (ZIP) [file pone.0212361.s002.zip › inclusion/In_190.bmp]

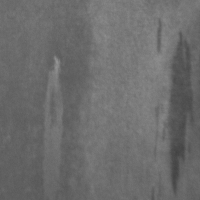

Supplement: S2 Dataset — The second typical strip steel surface defects dataset. (ZIP) [file pone.0212361.s002.zip › inclusion/In_191.bmp]

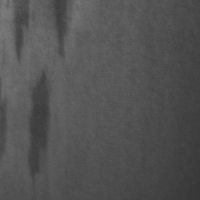

Supplement: S2 Dataset — The second typical strip steel surface defects dataset. (ZIP) [file pone.0212361.s002.zip › inclusion/In_192.bmp]

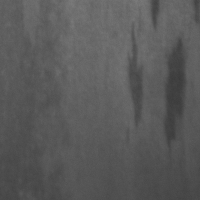

Supplement: S2 Dataset — The second typical strip steel surface defects dataset. (ZIP) [file pone.0212361.s002.zip › inclusion/In_193.bmp]

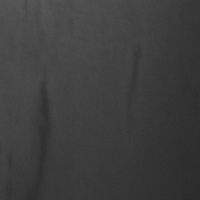

Supplement: S2 Dataset — The second typical strip steel surface defects dataset. (ZIP) [file pone.0212361.s002.zip › inclusion/In_194.bmp]

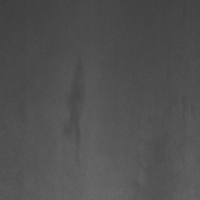

Supplement: S2 Dataset — The second typical strip steel surface defects dataset. (ZIP) [file pone.0212361.s002.zip › inclusion/In_195.bmp]

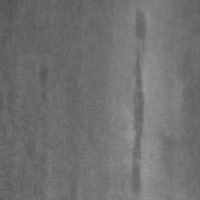

Supplement: S2 Dataset — The second typical strip steel surface defects dataset. (ZIP) [file pone.0212361.s002.zip › inclusion/In_196.bmp]

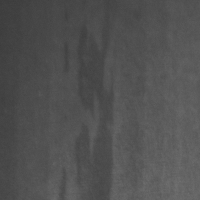

Supplement: S2 Dataset — The second typical strip steel surface defects dataset. (ZIP) [file pone.0212361.s002.zip › inclusion/In_197.bmp]

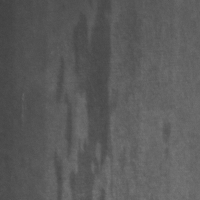

Supplement: S2 Dataset — The second typical strip steel surface defects dataset. (ZIP) [file pone.0212361.s002.zip › inclusion/In_198.bmp]

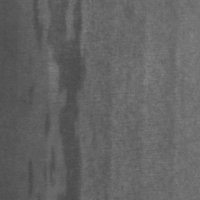

Supplement: S2 Dataset — The second typical strip steel surface defects dataset. (ZIP) [file pone.0212361.s002.zip › inclusion/In_199.bmp]

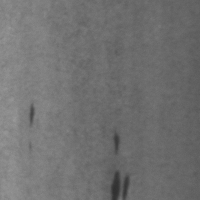

Supplement: S2 Dataset — The second typical strip steel surface defects dataset. (ZIP) [file pone.0212361.s002.zip › inclusion/In_2.bmp]

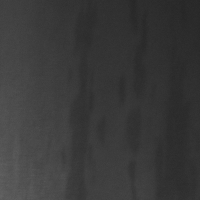

Supplement: S2 Dataset — The second typical strip steel surface defects dataset. (ZIP) [file pone.0212361.s002.zip › inclusion/In_20.bmp]

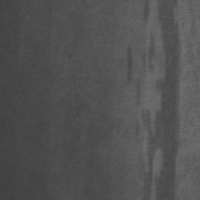

Supplement: S2 Dataset — The second typical strip steel surface defects dataset. (ZIP) [file pone.0212361.s002.zip › inclusion/In_200.bmp]

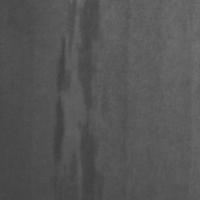

Supplement: S2 Dataset — The second typical strip steel surface defects dataset. (ZIP) [file pone.0212361.s002.zip › inclusion/In_201.bmp]

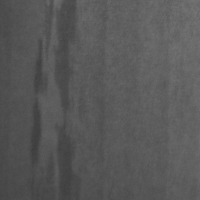

Supplement: S2 Dataset — The second typical strip steel surface defects dataset. (ZIP) [file pone.0212361.s002.zip › inclusion/In_202.bmp]

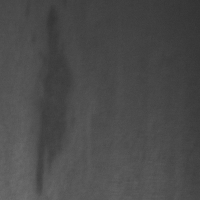

Supplement: S2 Dataset — The second typical strip steel surface defects dataset. (ZIP) [file pone.0212361.s002.zip › inclusion/In_203.bmp]

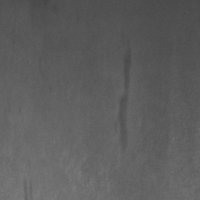

Supplement: S2 Dataset — The second typical strip steel surface defects dataset. (ZIP) [file pone.0212361.s002.zip › inclusion/In_204.bmp]

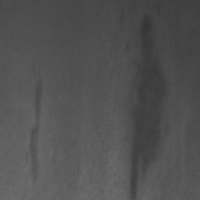

Supplement: S2 Dataset — The second typical strip steel surface defects dataset. (ZIP) [file pone.0212361.s002.zip › inclusion/In_205.bmp]

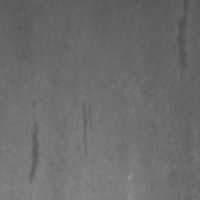

Supplement: S2 Dataset — The second typical strip steel surface defects dataset. (ZIP) [file pone.0212361.s002.zip › inclusion/In_206.bmp]

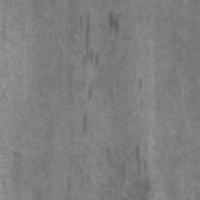

Supplement: S2 Dataset — The second typical strip steel surface defects dataset. (ZIP) [file pone.0212361.s002.zip › inclusion/In_207.bmp]

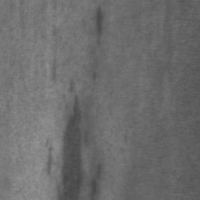

Supplement: S2 Dataset — The second typical strip steel surface defects dataset. (ZIP) [file pone.0212361.s002.zip › inclusion/In_208.bmp]

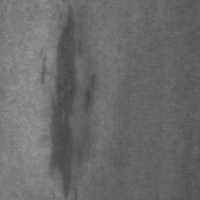

Supplement: S2 Dataset — The second typical strip steel surface defects dataset. (ZIP) [file pone.0212361.s002.zip › inclusion/In_209.bmp]

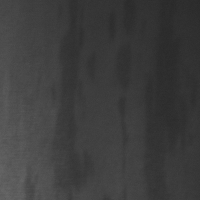

Supplement: S2 Dataset — The second typical strip steel surface defects dataset. (ZIP) [file pone.0212361.s002.zip › inclusion/In_21.bmp]

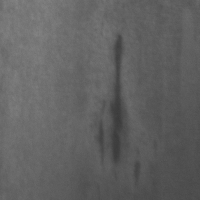

Supplement: S2 Dataset — The second typical strip steel surface defects dataset. (ZIP) [file pone.0212361.s002.zip › inclusion/In_210.bmp]

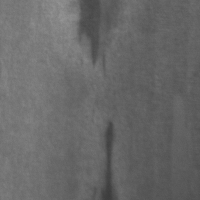

Supplement: S2 Dataset — The second typical strip steel surface defects dataset. (ZIP) [file pone.0212361.s002.zip › inclusion/In_211.bmp]

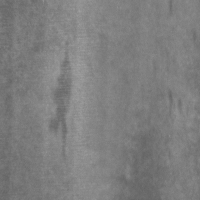

Supplement: S2 Dataset — The second typical strip steel surface defects dataset. (ZIP) [file pone.0212361.s002.zip › inclusion/In_212.bmp]

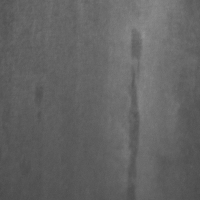

Supplement: S2 Dataset — The second typical strip steel surface defects dataset. (ZIP) [file pone.0212361.s002.zip › inclusion/In_213.bmp]

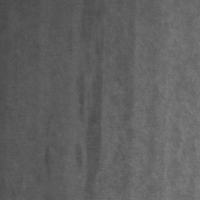

Supplement: S2 Dataset — The second typical strip steel surface defects dataset. (ZIP) [file pone.0212361.s002.zip › inclusion/In_214.bmp]

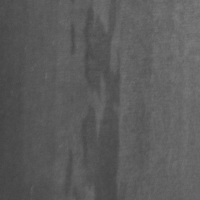

Supplement: S2 Dataset — The second typical strip steel surface defects dataset. (ZIP) [file pone.0212361.s002.zip › inclusion/In_215.bmp]

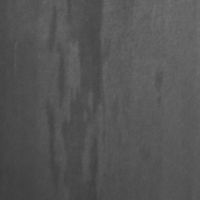

Supplement: S2 Dataset — The second typical strip steel surface defects dataset. (ZIP) [file pone.0212361.s002.zip › inclusion/In_216.bmp]

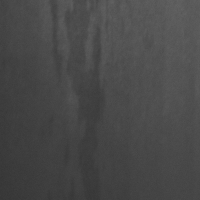

Supplement: S2 Dataset — The second typical strip steel surface defects dataset. (ZIP) [file pone.0212361.s002.zip › inclusion/In_217.bmp]

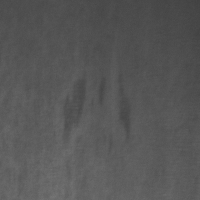

Supplement: S2 Dataset — The second typical strip steel surface defects dataset. (ZIP) [file pone.0212361.s002.zip › inclusion/In_218.bmp]

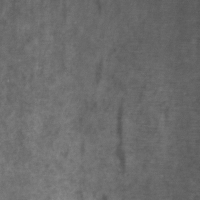

Supplement: S2 Dataset — The second typical strip steel surface defects dataset. (ZIP) [file pone.0212361.s002.zip › inclusion/In_219.bmp]

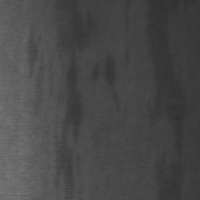

Supplement: S2 Dataset — The second typical strip steel surface defects dataset. (ZIP) [file pone.0212361.s002.zip › inclusion/In_22.bmp]

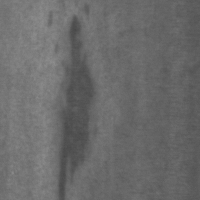

Supplement: S2 Dataset — The second typical strip steel surface defects dataset. (ZIP) [file pone.0212361.s002.zip › inclusion/In_220.bmp]

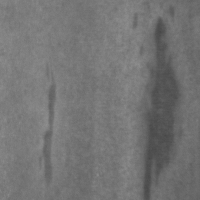

Supplement: S2 Dataset — The second typical strip steel surface defects dataset. (ZIP) [file pone.0212361.s002.zip › inclusion/In_221.bmp]

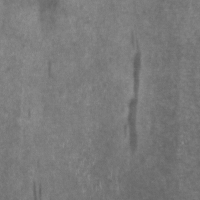

Supplement: S2 Dataset — The second typical strip steel surface defects dataset. (ZIP) [file pone.0212361.s002.zip › inclusion/In_222.bmp]

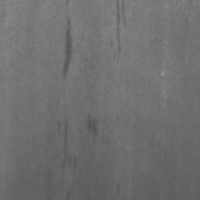

Supplement: S2 Dataset — The second typical strip steel surface defects dataset. (ZIP) [file pone.0212361.s002.zip › inclusion/In_223.bmp]

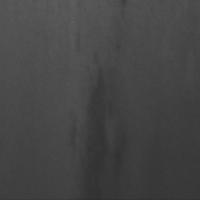

Supplement: S2 Dataset — The second typical strip steel surface defects dataset. (ZIP) [file pone.0212361.s002.zip › inclusion/In_224.bmp]

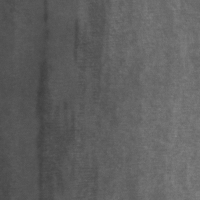

Supplement: S2 Dataset — The second typical strip steel surface defects dataset. (ZIP) [file pone.0212361.s002.zip › inclusion/In_225.bmp]

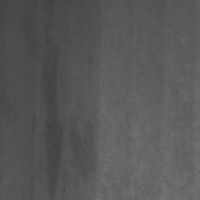

Supplement: S2 Dataset — The second typical strip steel surface defects dataset. (ZIP) [file pone.0212361.s002.zip › inclusion/In_226.bmp]

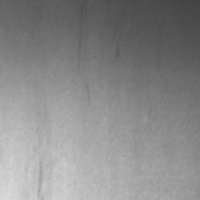

Supplement: S2 Dataset — The second typical strip steel surface defects dataset. (ZIP) [file pone.0212361.s002.zip › inclusion/In_227.bmp]

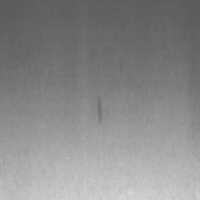

Supplement: S2 Dataset — The second typical strip steel surface defects dataset. (ZIP) [file pone.0212361.s002.zip › inclusion/In_228.bmp]

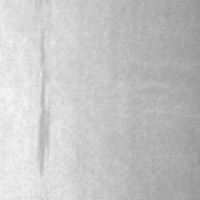

Supplement: S2 Dataset — The second typical strip steel surface defects dataset. (ZIP) [file pone.0212361.s002.zip › inclusion/In_229.bmp]

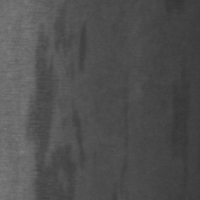

Supplement: S2 Dataset — The second typical strip steel surface defects dataset. (ZIP) [file pone.0212361.s002.zip › inclusion/In_23.bmp]

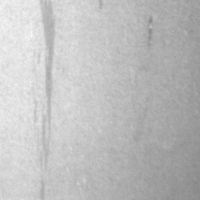

Supplement: S2 Dataset — The second typical strip steel surface defects dataset. (ZIP) [file pone.0212361.s002.zip › inclusion/In_230.bmp]

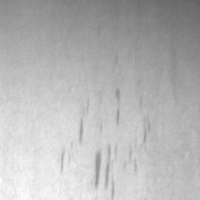

Supplement: S2 Dataset — The second typical strip steel surface defects dataset. (ZIP) [file pone.0212361.s002.zip › inclusion/In_231.bmp]

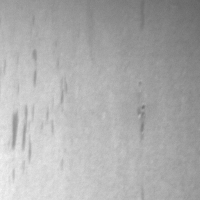

Supplement: S2 Dataset — The second typical strip steel surface defects dataset. (ZIP) [file pone.0212361.s002.zip › inclusion/In_232.bmp]

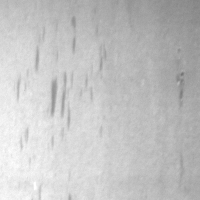

Supplement: S2 Dataset — The second typical strip steel surface defects dataset. (ZIP) [file pone.0212361.s002.zip › inclusion/In_233.bmp]

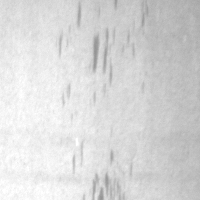

Supplement: S2 Dataset — The second typical strip steel surface defects dataset. (ZIP) [file pone.0212361.s002.zip › inclusion/In_234.bmp]

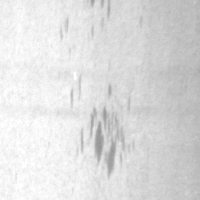

Supplement: S2 Dataset — The second typical strip steel surface defects dataset. (ZIP) [file pone.0212361.s002.zip › inclusion/In_235.bmp]

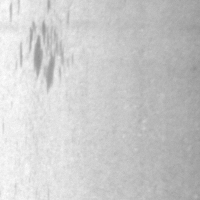

Supplement: S2 Dataset — The second typical strip steel surface defects dataset. (ZIP) [file pone.0212361.s002.zip › inclusion/In_236.bmp]

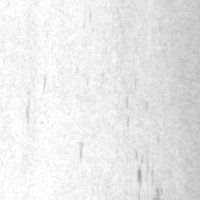

Supplement: S2 Dataset — The second typical strip steel surface defects dataset. (ZIP) [file pone.0212361.s002.zip › inclusion/In_237.bmp]

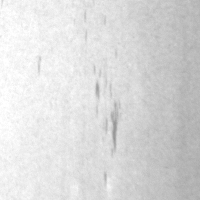

Supplement: S2 Dataset — The second typical strip steel surface defects dataset. (ZIP) [file pone.0212361.s002.zip › inclusion/In_238.bmp]

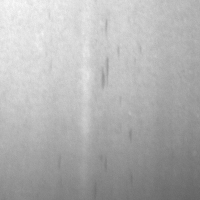

Supplement: S2 Dataset — The second typical strip steel surface defects dataset. (ZIP) [file pone.0212361.s002.zip › inclusion/In_239.bmp]

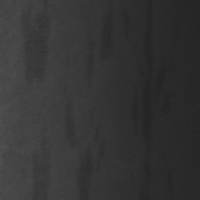

Supplement: S2 Dataset — The second typical strip steel surface defects dataset. (ZIP) [file pone.0212361.s002.zip › inclusion/In_24.bmp]

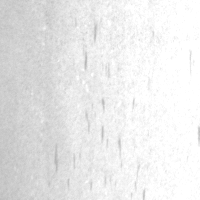

Supplement: S2 Dataset — The second typical strip steel surface defects dataset. (ZIP) [file pone.0212361.s002.zip › inclusion/In_240.bmp]

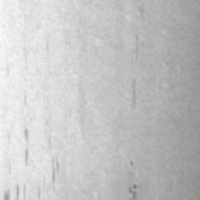

Supplement: S2 Dataset — The second typical strip steel surface defects dataset. (ZIP) [file pone.0212361.s002.zip › inclusion/In_241.bmp]

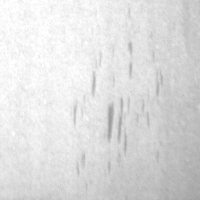

Supplement: S2 Dataset — The second typical strip steel surface defects dataset. (ZIP) [file pone.0212361.s002.zip › inclusion/In_242.bmp]

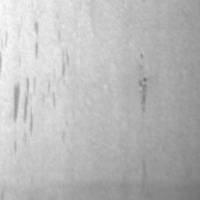

Supplement: S2 Dataset — The second typical strip steel surface defects dataset. (ZIP) [file pone.0212361.s002.zip › inclusion/In_243.bmp]

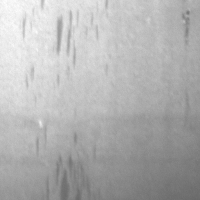

Supplement: S2 Dataset — The second typical strip steel surface defects dataset. (ZIP) [file pone.0212361.s002.zip › inclusion/In_244.bmp]

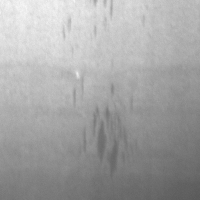

Supplement: S2 Dataset — The second typical strip steel surface defects dataset. (ZIP) [file pone.0212361.s002.zip › inclusion/In_245.bmp]

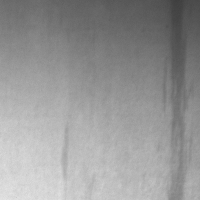

Supplement: S2 Dataset — The second typical strip steel surface defects dataset. (ZIP) [file pone.0212361.s002.zip › inclusion/In_246.bmp]

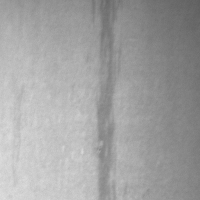

Supplement: S2 Dataset — The second typical strip steel surface defects dataset. (ZIP) [file pone.0212361.s002.zip › inclusion/In_247.bmp]

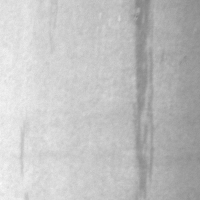

Supplement: S2 Dataset — The second typical strip steel surface defects dataset. (ZIP) [file pone.0212361.s002.zip › inclusion/In_248.bmp]

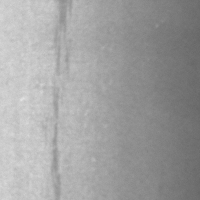

Supplement: S2 Dataset — The second typical strip steel surface defects dataset. (ZIP) [file pone.0212361.s002.zip › inclusion/In_249.bmp]

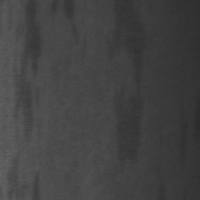

Supplement: S2 Dataset — The second typical strip steel surface defects dataset. (ZIP) [file pone.0212361.s002.zip › inclusion/In_25.bmp]

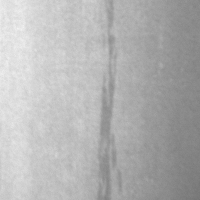

Supplement: S2 Dataset — The second typical strip steel surface defects dataset. (ZIP) [file pone.0212361.s002.zip › inclusion/In_250.bmp]

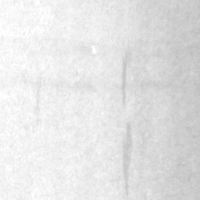

Supplement: S2 Dataset — The second typical strip steel surface defects dataset. (ZIP) [file pone.0212361.s002.zip › inclusion/In_251.bmp]

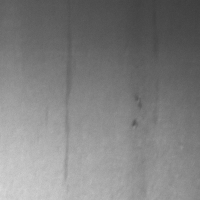

Supplement: S2 Dataset — The second typical strip steel surface defects dataset. (ZIP) [file pone.0212361.s002.zip › inclusion/In_252.bmp]

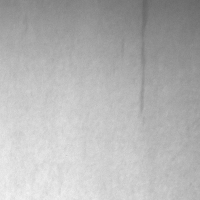

Supplement: S2 Dataset — The second typical strip steel surface defects dataset. (ZIP) [file pone.0212361.s002.zip › inclusion/In_253.bmp]

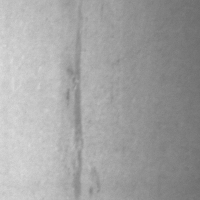

Supplement: S2 Dataset — The second typical strip steel surface defects dataset. (ZIP) [file pone.0212361.s002.zip › inclusion/In_254.bmp]

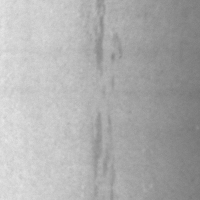

Supplement: S2 Dataset — The second typical strip steel surface defects dataset. (ZIP) [file pone.0212361.s002.zip › inclusion/In_255.bmp]

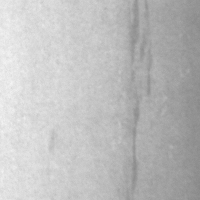

Supplement: S2 Dataset — The second typical strip steel surface defects dataset. (ZIP) [file pone.0212361.s002.zip › inclusion/In_256.bmp]

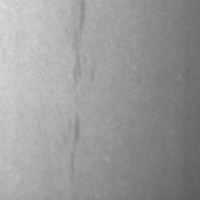

Supplement: S2 Dataset — The second typical strip steel surface defects dataset. (ZIP) [file pone.0212361.s002.zip › inclusion/In_257.bmp]

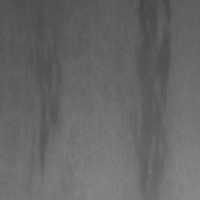

Supplement: S2 Dataset — The second typical strip steel surface defects dataset. (ZIP) [file pone.0212361.s002.zip › inclusion/In_258.bmp]

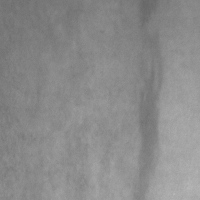

Supplement: S2 Dataset — The second typical strip steel surface defects dataset. (ZIP) [file pone.0212361.s002.zip › inclusion/In_259.bmp]

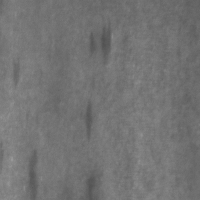

Supplement: S2 Dataset — The second typical strip steel surface defects dataset. (ZIP) [file pone.0212361.s002.zip › inclusion/In_26.bmp]

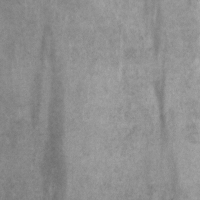

Supplement: S2 Dataset — The second typical strip steel surface defects dataset. (ZIP) [file pone.0212361.s002.zip › inclusion/In_260.bmp]

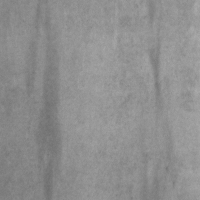

Supplement: S2 Dataset — The second typical strip steel surface defects dataset. (ZIP) [file pone.0212361.s002.zip › inclusion/In_261.bmp]

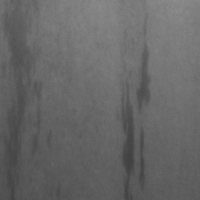

Supplement: S2 Dataset — The second typical strip steel surface defects dataset. (ZIP) [file pone.0212361.s002.zip › inclusion/In_262.bmp]

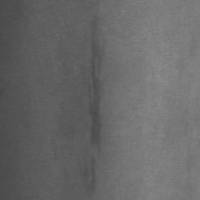

Supplement: S2 Dataset — The second typical strip steel surface defects dataset. (ZIP) [file pone.0212361.s002.zip › inclusion/In_263.bmp]

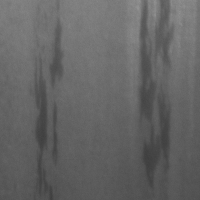

Supplement: S2 Dataset — The second typical strip steel surface defects dataset. (ZIP) [file pone.0212361.s002.zip › inclusion/In_264.bmp]

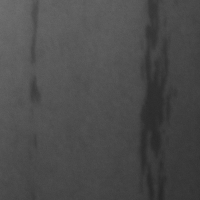

Supplement: S2 Dataset — The second typical strip steel surface defects dataset. (ZIP) [file pone.0212361.s002.zip › inclusion/In_265.bmp]

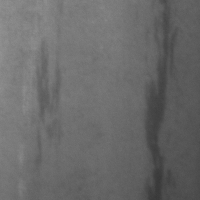

Supplement: S2 Dataset — The second typical strip steel surface defects dataset. (ZIP) [file pone.0212361.s002.zip › inclusion/In_266.bmp]

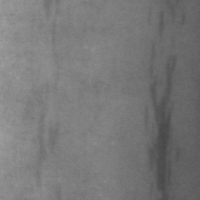

Supplement: S2 Dataset — The second typical strip steel surface defects dataset. (ZIP) [file pone.0212361.s002.zip › inclusion/In_267.bmp]

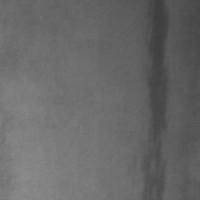

Supplement: S2 Dataset — The second typical strip steel surface defects dataset. (ZIP) [file pone.0212361.s002.zip › inclusion/In_268.bmp]

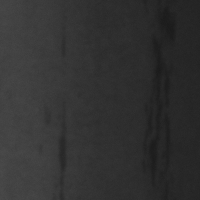

Supplement: S2 Dataset — The second typical strip steel surface defects dataset. (ZIP) [file pone.0212361.s002.zip › inclusion/In_269.bmp]

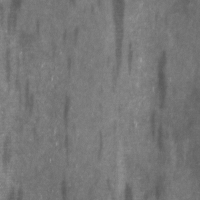

Supplement: S2 Dataset — The second typical strip steel surface defects dataset. (ZIP) [file pone.0212361.s002.zip › inclusion/In_27.bmp]

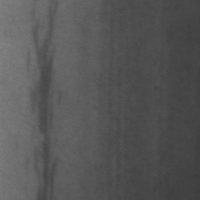

Supplement: S2 Dataset — The second typical strip steel surface defects dataset. (ZIP) [file pone.0212361.s002.zip › inclusion/In_270.bmp]

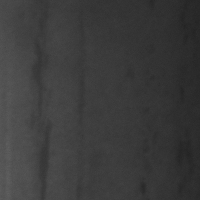

Supplement: S2 Dataset — The second typical strip steel surface defects dataset. (ZIP) [file pone.0212361.s002.zip › inclusion/In_271.bmp]

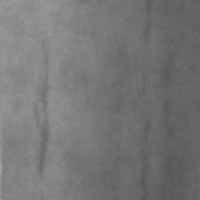

Supplement: S2 Dataset — The second typical strip steel surface defects dataset. (ZIP) [file pone.0212361.s002.zip › inclusion/In_272.bmp]

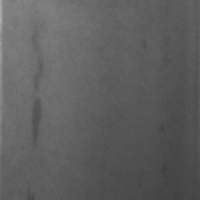

Supplement: S2 Dataset — The second typical strip steel surface defects dataset. (ZIP) [file pone.0212361.s002.zip › inclusion/In_273.bmp]

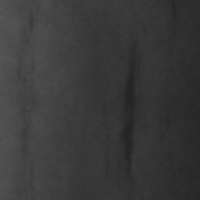

Supplement: S2 Dataset — The second typical strip steel surface defects dataset. (ZIP) [file pone.0212361.s002.zip › inclusion/In_274.bmp]

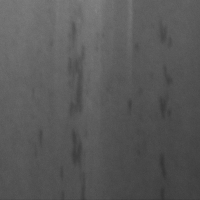

Supplement: S2 Dataset — The second typical strip steel surface defects dataset. (ZIP) [file pone.0212361.s002.zip › inclusion/In_275.bmp]

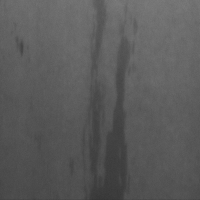

Supplement: S2 Dataset — The second typical strip steel surface defects dataset. (ZIP) [file pone.0212361.s002.zip › inclusion/In_276.bmp]

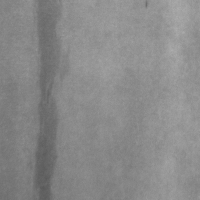

Supplement: S2 Dataset — The second typical strip steel surface defects dataset. (ZIP) [file pone.0212361.s002.zip › inclusion/In_277.bmp]

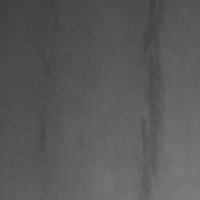

Supplement: S2 Dataset — The second typical strip steel surface defects dataset. (ZIP) [file pone.0212361.s002.zip › inclusion/In_278.bmp]

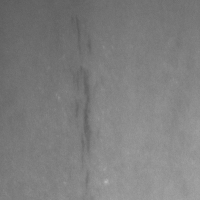

Supplement: S2 Dataset — The second typical strip steel surface defects dataset. (ZIP) [file pone.0212361.s002.zip › inclusion/In_279.bmp]
