# Supplementary figures and images for: Support vector machine with quantile hyper-spheres for pattern classification (part 1 of 6)
Source: PLoS One. 2019 Feb 15;14(2):e0212361. doi: 10.1371/journal.pone.0212361 (PMC6377146; doi:10.1371/journal.pone.0212361)

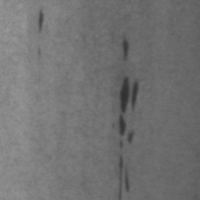

Supplement: S2 Dataset — The second typical strip steel surface defects dataset. (ZIP) [file pone.0212361.s002.zip › inclusion/In_1.bmp]

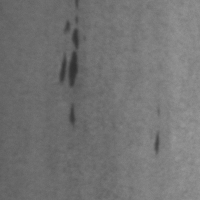

Supplement: S2 Dataset — The second typical strip steel surface defects dataset. (ZIP) [file pone.0212361.s002.zip › inclusion/In_10.bmp]

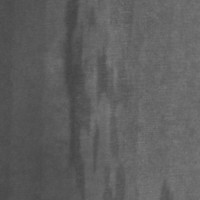

Supplement: S2 Dataset — The second typical strip steel surface defects dataset. (ZIP) [file pone.0212361.s002.zip › inclusion/In_100.bmp]

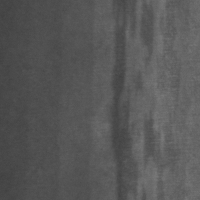

Supplement: S2 Dataset — The second typical strip steel surface defects dataset. (ZIP) [file pone.0212361.s002.zip › inclusion/In_101.bmp]

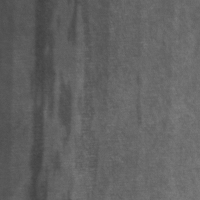

Supplement: S2 Dataset — The second typical strip steel surface defects dataset. (ZIP) [file pone.0212361.s002.zip › inclusion/In_102.bmp]

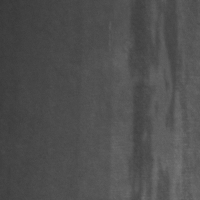

Supplement: S2 Dataset — The second typical strip steel surface defects dataset. (ZIP) [file pone.0212361.s002.zip › inclusion/In_103.bmp]

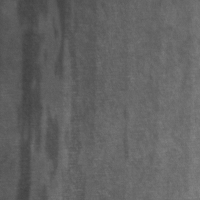

Supplement: S2 Dataset — The second typical strip steel surface defects dataset. (ZIP) [file pone.0212361.s002.zip › inclusion/In_104.bmp]

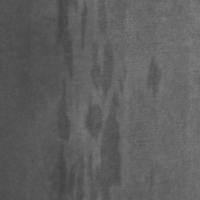

Supplement: S2 Dataset — The second typical strip steel surface defects dataset. (ZIP) [file pone.0212361.s002.zip › inclusion/In_105.bmp]

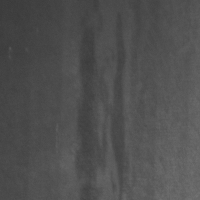

Supplement: S2 Dataset — The second typical strip steel surface defects dataset. (ZIP) [file pone.0212361.s002.zip › inclusion/In_106.bmp]

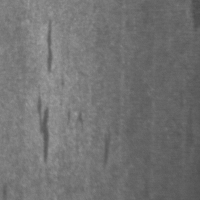

Supplement: S2 Dataset — The second typical strip steel surface defects dataset. (ZIP) [file pone.0212361.s002.zip › inclusion/In_107.bmp]

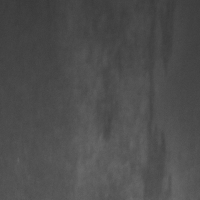

Supplement: S2 Dataset — The second typical strip steel surface defects dataset. (ZIP) [file pone.0212361.s002.zip › inclusion/In_108.bmp]

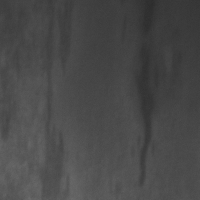

Supplement: S2 Dataset — The second typical strip steel surface defects dataset. (ZIP) [file pone.0212361.s002.zip › inclusion/In_109.bmp]

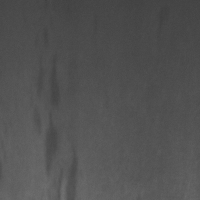

Supplement: S2 Dataset — The second typical strip steel surface defects dataset. (ZIP) [file pone.0212361.s002.zip › inclusion/In_11.bmp]

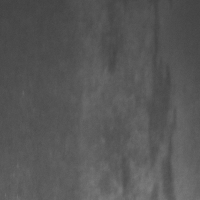

Supplement: S2 Dataset — The second typical strip steel surface defects dataset. (ZIP) [file pone.0212361.s002.zip › inclusion/In_110.bmp]

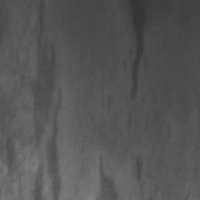

Supplement: S2 Dataset — The second typical strip steel surface defects dataset. (ZIP) [file pone.0212361.s002.zip › inclusion/In_111.bmp]

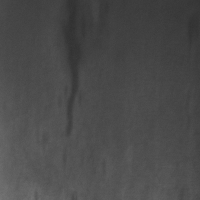

Supplement: S2 Dataset — The second typical strip steel surface defects dataset. (ZIP) [file pone.0212361.s002.zip › inclusion/In_112.bmp]

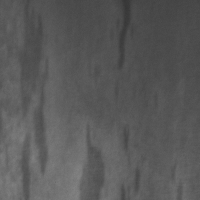

Supplement: S2 Dataset — The second typical strip steel surface defects dataset. (ZIP) [file pone.0212361.s002.zip › inclusion/In_113.bmp]

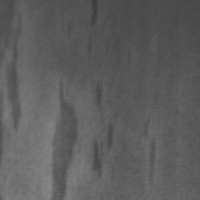

Supplement: S2 Dataset — The second typical strip steel surface defects dataset. (ZIP) [file pone.0212361.s002.zip › inclusion/In_114.bmp]

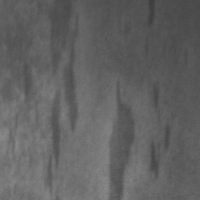

Supplement: S2 Dataset — The second typical strip steel surface defects dataset. (ZIP) [file pone.0212361.s002.zip › inclusion/In_115.bmp]

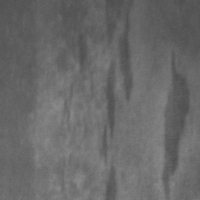

Supplement: S2 Dataset — The second typical strip steel surface defects dataset. (ZIP) [file pone.0212361.s002.zip › inclusion/In_116.bmp]

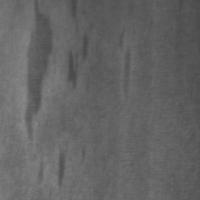

Supplement: S2 Dataset — The second typical strip steel surface defects dataset. (ZIP) [file pone.0212361.s002.zip › inclusion/In_117.bmp]

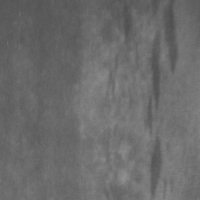

Supplement: S2 Dataset — The second typical strip steel surface defects dataset. (ZIP) [file pone.0212361.s002.zip › inclusion/In_118.bmp]

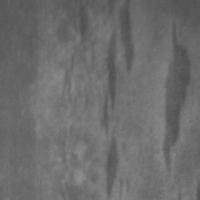

Supplement: S2 Dataset — The second typical strip steel surface defects dataset. (ZIP) [file pone.0212361.s002.zip › inclusion/In_119.bmp]

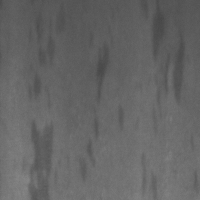

Supplement: S2 Dataset — The second typical strip steel surface defects dataset. (ZIP) [file pone.0212361.s002.zip › inclusion/In_12.bmp]

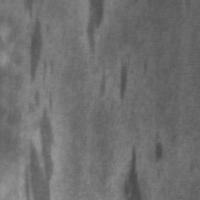

Supplement: S2 Dataset — The second typical strip steel surface defects dataset. (ZIP) [file pone.0212361.s002.zip › inclusion/In_120.bmp]

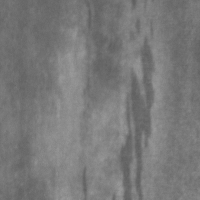

Supplement: S2 Dataset — The second typical strip steel surface defects dataset. (ZIP) [file pone.0212361.s002.zip › inclusion/In_121.bmp]

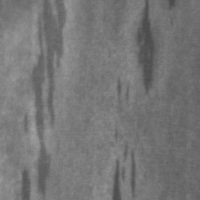

Supplement: S2 Dataset — The second typical strip steel surface defects dataset. (ZIP) [file pone.0212361.s002.zip › inclusion/In_122.bmp]

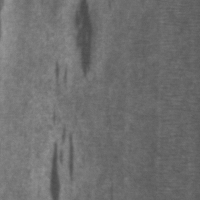

Supplement: S2 Dataset — The second typical strip steel surface defects dataset. (ZIP) [file pone.0212361.s002.zip › inclusion/In_123.bmp]

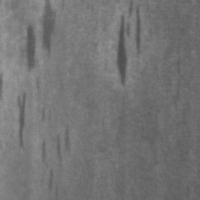

Supplement: S2 Dataset — The second typical strip steel surface defects dataset. (ZIP) [file pone.0212361.s002.zip › inclusion/In_124.bmp]

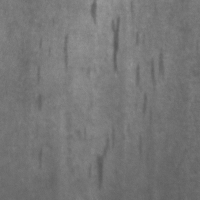

Supplement: S2 Dataset — The second typical strip steel surface defects dataset. (ZIP) [file pone.0212361.s002.zip › inclusion/In_125.bmp]

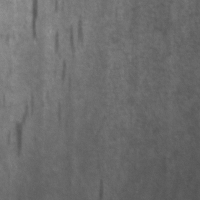

Supplement: S2 Dataset — The second typical strip steel surface defects dataset. (ZIP) [file pone.0212361.s002.zip › inclusion/In_126.bmp]

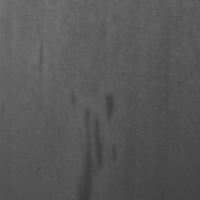

Supplement: S2 Dataset — The second typical strip steel surface defects dataset. (ZIP) [file pone.0212361.s002.zip › inclusion/In_127.bmp]

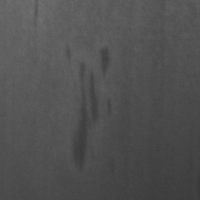

Supplement: S2 Dataset — The second typical strip steel surface defects dataset. (ZIP) [file pone.0212361.s002.zip › inclusion/In_128.bmp]

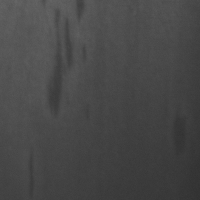

Supplement: S2 Dataset — The second typical strip steel surface defects dataset. (ZIP) [file pone.0212361.s002.zip › inclusion/In_129.bmp]

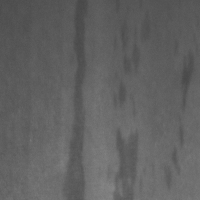

Supplement: S2 Dataset — The second typical strip steel surface defects dataset. (ZIP) [file pone.0212361.s002.zip › inclusion/In_13.bmp]

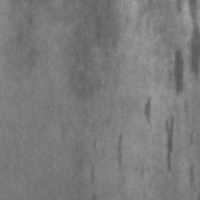

Supplement: S2 Dataset — The second typical strip steel surface defects dataset. (ZIP) [file pone.0212361.s002.zip › inclusion/In_130.bmp]

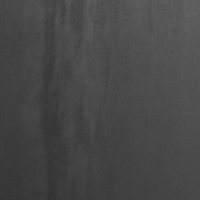

Supplement: S2 Dataset — The second typical strip steel surface defects dataset. (ZIP) [file pone.0212361.s002.zip › inclusion/In_131.bmp]

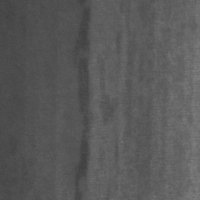

Supplement: S2 Dataset — The second typical strip steel surface defects dataset. (ZIP) [file pone.0212361.s002.zip › inclusion/In_132.bmp]

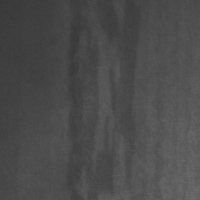

Supplement: S2 Dataset — The second typical strip steel surface defects dataset. (ZIP) [file pone.0212361.s002.zip › inclusion/In_133.bmp]

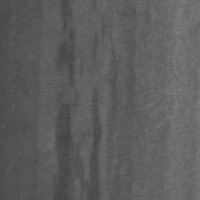

Supplement: S2 Dataset — The second typical strip steel surface defects dataset. (ZIP) [file pone.0212361.s002.zip › inclusion/In_134.bmp]

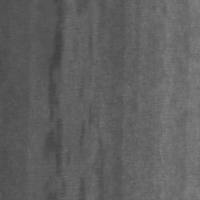

Supplement: S2 Dataset — The second typical strip steel surface defects dataset. (ZIP) [file pone.0212361.s002.zip › inclusion/In_135.bmp]

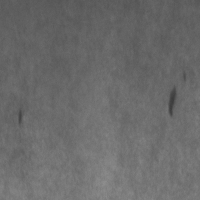

Supplement: S2 Dataset — The second typical strip steel surface defects dataset. (ZIP) [file pone.0212361.s002.zip › inclusion/In_136.bmp]

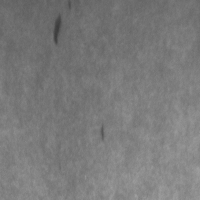

Supplement: S2 Dataset — The second typical strip steel surface defects dataset. (ZIP) [file pone.0212361.s002.zip › inclusion/In_137.bmp]

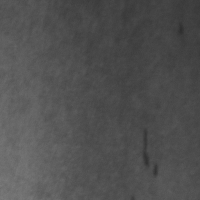

Supplement: S2 Dataset — The second typical strip steel surface defects dataset. (ZIP) [file pone.0212361.s002.zip › inclusion/In_138.bmp]

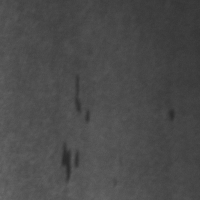

Supplement: S2 Dataset — The second typical strip steel surface defects dataset. (ZIP) [file pone.0212361.s002.zip › inclusion/In_139.bmp]

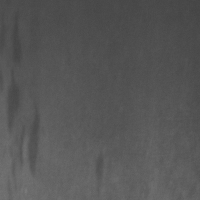

Supplement: S2 Dataset — The second typical strip steel surface defects dataset. (ZIP) [file pone.0212361.s002.zip › inclusion/In_14.bmp]

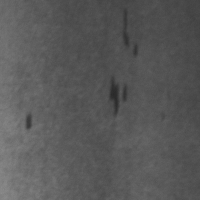

Supplement: S2 Dataset — The second typical strip steel surface defects dataset. (ZIP) [file pone.0212361.s002.zip › inclusion/In_140.bmp]

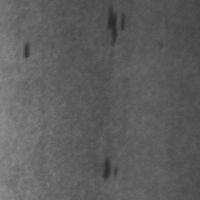

Supplement: S2 Dataset — The second typical strip steel surface defects dataset. (ZIP) [file pone.0212361.s002.zip › inclusion/In_141.bmp]

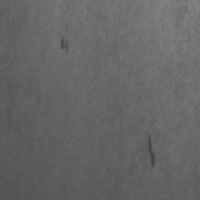

Supplement: S2 Dataset — The second typical strip steel surface defects dataset. (ZIP) [file pone.0212361.s002.zip › inclusion/In_142.bmp]

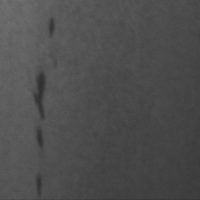

Supplement: S2 Dataset — The second typical strip steel surface defects dataset. (ZIP) [file pone.0212361.s002.zip › inclusion/In_143.bmp]

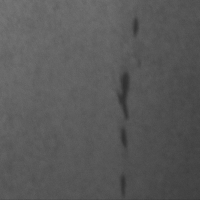

Supplement: S2 Dataset — The second typical strip steel surface defects dataset. (ZIP) [file pone.0212361.s002.zip › inclusion/In_144.bmp]

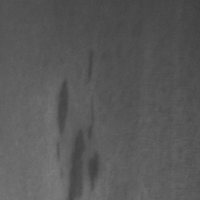

Supplement: S2 Dataset — The second typical strip steel surface defects dataset. (ZIP) [file pone.0212361.s002.zip › inclusion/In_145.bmp]

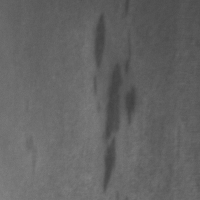

Supplement: S2 Dataset — The second typical strip steel surface defects dataset. (ZIP) [file pone.0212361.s002.zip › inclusion/In_146.bmp]

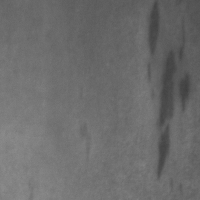

Supplement: S2 Dataset — The second typical strip steel surface defects dataset. (ZIP) [file pone.0212361.s002.zip › inclusion/In_147.bmp]

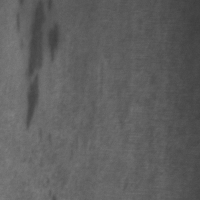

Supplement: S2 Dataset — The second typical strip steel surface defects dataset. (ZIP) [file pone.0212361.s002.zip › inclusion/In_148.bmp]

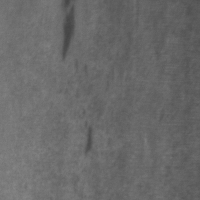

Supplement: S2 Dataset — The second typical strip steel surface defects dataset. (ZIP) [file pone.0212361.s002.zip › inclusion/In_149.bmp]

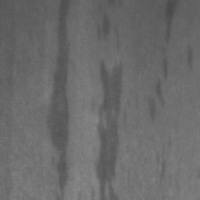

Supplement: S2 Dataset — The second typical strip steel surface defects dataset. (ZIP) [file pone.0212361.s002.zip › inclusion/In_15.bmp]

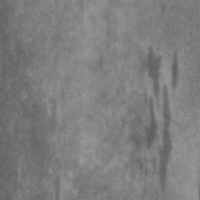

Supplement: S2 Dataset — The second typical strip steel surface defects dataset. (ZIP) [file pone.0212361.s002.zip › inclusion/In_150.bmp]

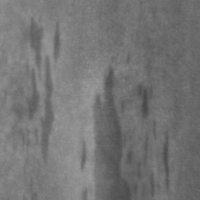

Supplement: S2 Dataset — The second typical strip steel surface defects dataset. (ZIP) [file pone.0212361.s002.zip › inclusion/In_151.bmp]

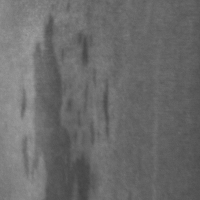

Supplement: S2 Dataset — The second typical strip steel surface defects dataset. (ZIP) [file pone.0212361.s002.zip › inclusion/In_152.bmp]

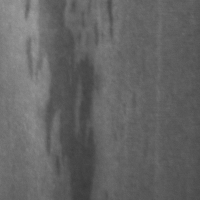

Supplement: S2 Dataset — The second typical strip steel surface defects dataset. (ZIP) [file pone.0212361.s002.zip › inclusion/In_153.bmp]

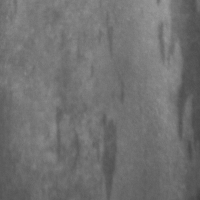

Supplement: S2 Dataset — The second typical strip steel surface defects dataset. (ZIP) [file pone.0212361.s002.zip › inclusion/In_154.bmp]

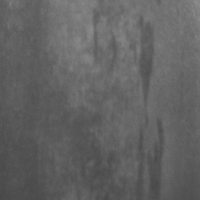

Supplement: S2 Dataset — The second typical strip steel surface defects dataset. (ZIP) [file pone.0212361.s002.zip › inclusion/In_155.bmp]

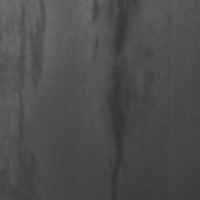

Supplement: S2 Dataset — The second typical strip steel surface defects dataset. (ZIP) [file pone.0212361.s002.zip › inclusion/In_156.bmp]

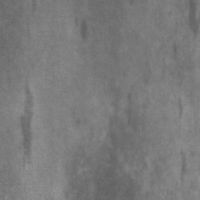

Supplement: S2 Dataset — The second typical strip steel surface defects dataset. (ZIP) [file pone.0212361.s002.zip › inclusion/In_157.bmp]

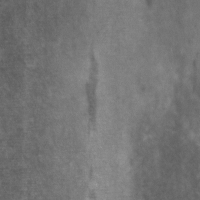

Supplement: S2 Dataset — The second typical strip steel surface defects dataset. (ZIP) [file pone.0212361.s002.zip › inclusion/In_158.bmp]

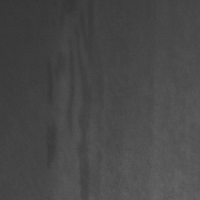

Supplement: S2 Dataset — The second typical strip steel surface defects dataset. (ZIP) [file pone.0212361.s002.zip › inclusion/In_159.bmp]

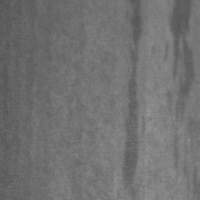

Supplement: S2 Dataset — The second typical strip steel surface defects dataset. (ZIP) [file pone.0212361.s002.zip › inclusion/In_16.bmp]

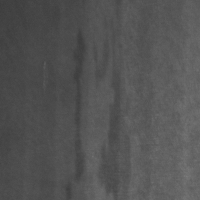

Supplement: S2 Dataset — The second typical strip steel surface defects dataset. (ZIP) [file pone.0212361.s002.zip › inclusion/In_160.bmp]

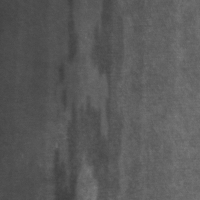

Supplement: S2 Dataset — The second typical strip steel surface defects dataset. (ZIP) [file pone.0212361.s002.zip › inclusion/In_161.bmp]

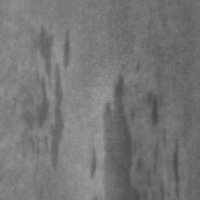

Supplement: S2 Dataset — The second typical strip steel surface defects dataset. (ZIP) [file pone.0212361.s002.zip › inclusion/In_162.bmp]

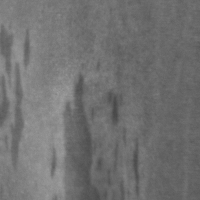

Supplement: S2 Dataset — The second typical strip steel surface defects dataset. (ZIP) [file pone.0212361.s002.zip › inclusion/In_163.bmp]

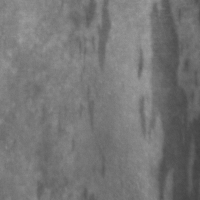

Supplement: S2 Dataset — The second typical strip steel surface defects dataset. (ZIP) [file pone.0212361.s002.zip › inclusion/In_164.bmp]

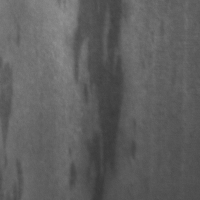

Supplement: S2 Dataset — The second typical strip steel surface defects dataset. (ZIP) [file pone.0212361.s002.zip › inclusion/In_165.bmp]

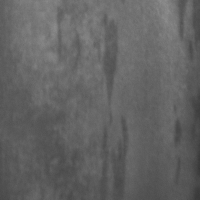

Supplement: S2 Dataset — The second typical strip steel surface defects dataset. (ZIP) [file pone.0212361.s002.zip › inclusion/In_166.bmp]

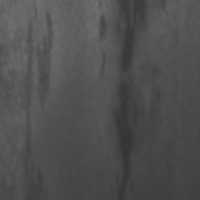

Supplement: S2 Dataset — The second typical strip steel surface defects dataset. (ZIP) [file pone.0212361.s002.zip › inclusion/In_167.bmp]

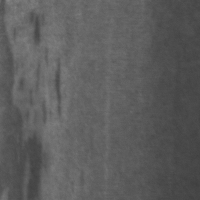

Supplement: S2 Dataset — The second typical strip steel surface defects dataset. (ZIP) [file pone.0212361.s002.zip › inclusion/In_168.bmp]

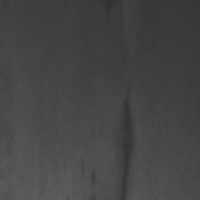

Supplement: S2 Dataset — The second typical strip steel surface defects dataset. (ZIP) [file pone.0212361.s002.zip › inclusion/In_169.bmp]

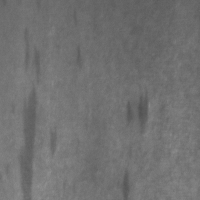

Supplement: S2 Dataset — The second typical strip steel surface defects dataset. (ZIP) [file pone.0212361.s002.zip › inclusion/In_17.bmp]

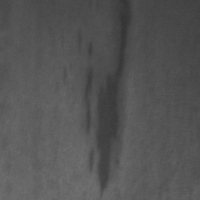

Supplement: S2 Dataset — The second typical strip steel surface defects dataset. (ZIP) [file pone.0212361.s002.zip › inclusion/In_170.bmp]

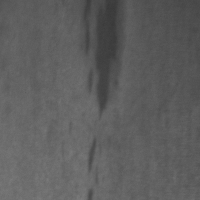

Supplement: S2 Dataset — The second typical strip steel surface defects dataset. (ZIP) [file pone.0212361.s002.zip › inclusion/In_171.bmp]

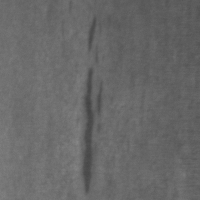

Supplement: S2 Dataset — The second typical strip steel surface defects dataset. (ZIP) [file pone.0212361.s002.zip › inclusion/In_172.bmp]

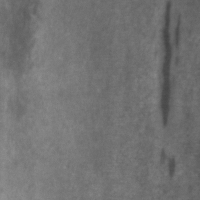

Supplement: S2 Dataset — The second typical strip steel surface defects dataset. (ZIP) [file pone.0212361.s002.zip › inclusion/In_173.bmp]

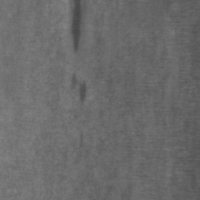

Supplement: S2 Dataset — The second typical strip steel surface defects dataset. (ZIP) [file pone.0212361.s002.zip › inclusion/In_174.bmp]

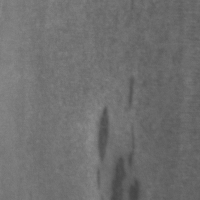

Supplement: S2 Dataset — The second typical strip steel surface defects dataset. (ZIP) [file pone.0212361.s002.zip › inclusion/In_175.bmp]

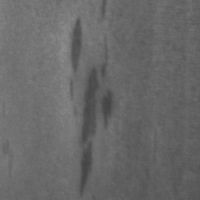

Supplement: S2 Dataset — The second typical strip steel surface defects dataset. (ZIP) [file pone.0212361.s002.zip › inclusion/In_176.bmp]

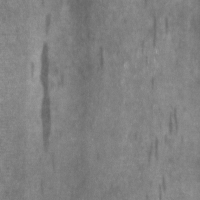

Supplement: S2 Dataset — The second typical strip steel surface defects dataset. (ZIP) [file pone.0212361.s002.zip › inclusion/In_177.bmp]

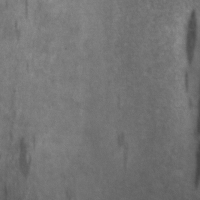

Supplement: S2 Dataset — The second typical strip steel surface defects dataset. (ZIP) [file pone.0212361.s002.zip › inclusion/In_178.bmp]

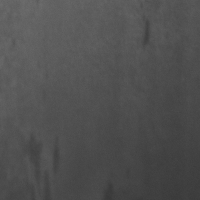

Supplement: S2 Dataset — The second typical strip steel surface defects dataset. (ZIP) [file pone.0212361.s002.zip › inclusion/In_179.bmp]

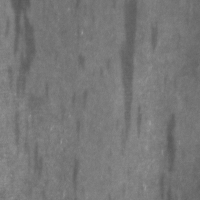

Supplement: S2 Dataset — The second typical strip steel surface defects dataset. (ZIP) [file pone.0212361.s002.zip › inclusion/In_18.bmp]

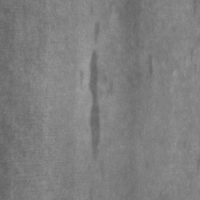

Supplement: S2 Dataset — The second typical strip steel surface defects dataset. (ZIP) [file pone.0212361.s002.zip › inclusion/In_180.bmp]

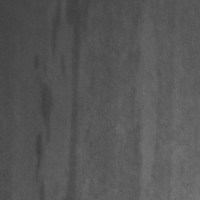

Supplement: S2 Dataset — The second typical strip steel surface defects dataset. (ZIP) [file pone.0212361.s002.zip › inclusion/In_181.bmp]

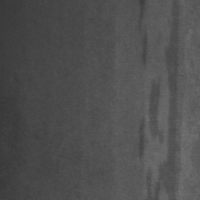

Supplement: S2 Dataset — The second typical strip steel surface defects dataset. (ZIP) [file pone.0212361.s002.zip › inclusion/In_182.bmp]

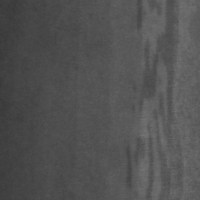

Supplement: S2 Dataset — The second typical strip steel surface defects dataset. (ZIP) [file pone.0212361.s002.zip › inclusion/In_183.bmp]

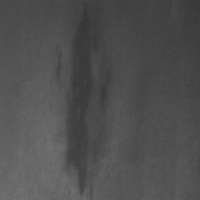

Supplement: S2 Dataset — The second typical strip steel surface defects dataset. (ZIP) [file pone.0212361.s002.zip › inclusion/In_184.bmp]

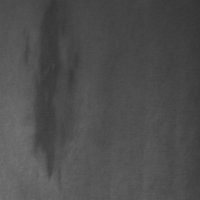

Supplement: S2 Dataset — The second typical strip steel surface defects dataset. (ZIP) [file pone.0212361.s002.zip › inclusion/In_185.bmp]

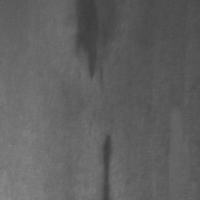

Supplement: S2 Dataset — The second typical strip steel surface defects dataset. (ZIP) [file pone.0212361.s002.zip › inclusion/In_186.bmp]

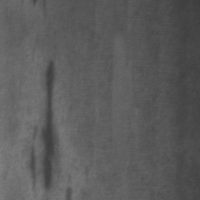

Supplement: S2 Dataset — The second typical strip steel surface defects dataset. (ZIP) [file pone.0212361.s002.zip › inclusion/In_187.bmp]

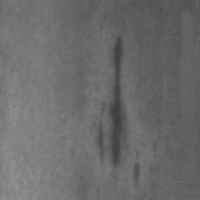

Supplement: S2 Dataset — The second typical strip steel surface defects dataset. (ZIP) [file pone.0212361.s002.zip › inclusion/In_188.bmp]

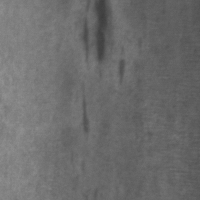

Supplement: S2 Dataset — The second typical strip steel surface defects dataset. (ZIP) [file pone.0212361.s002.zip › inclusion/In_189.bmp]
