# Supplementary figures and images for: Support vector machine with quantile hyper-spheres for pattern classification (part 4 of 6)
Source: PLoS One. 2019 Feb 15;14(2):e0212361. doi: 10.1371/journal.pone.0212361 (PMC6377146; doi:10.1371/journal.pone.0212361)

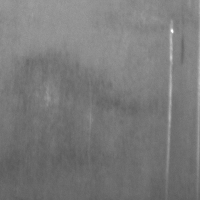

Supplement: S3 Dataset — The third typical strip steel surface defects dataset. (ZIP) [file pone.0212361.s003.zip › scratches/Sc_19.bmp]

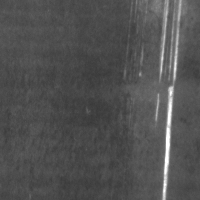

Supplement: S3 Dataset — The third typical strip steel surface defects dataset. (ZIP) [file pone.0212361.s003.zip › scratches/Sc_190.bmp]

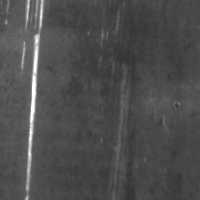

Supplement: S3 Dataset — The third typical strip steel surface defects dataset. (ZIP) [file pone.0212361.s003.zip › scratches/Sc_191.bmp]

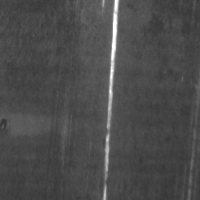

Supplement: S3 Dataset — The third typical strip steel surface defects dataset. (ZIP) [file pone.0212361.s003.zip › scratches/Sc_192.bmp]

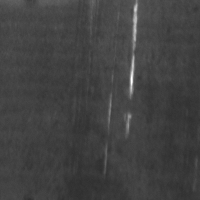

Supplement: S3 Dataset — The third typical strip steel surface defects dataset. (ZIP) [file pone.0212361.s003.zip › scratches/Sc_193.bmp]

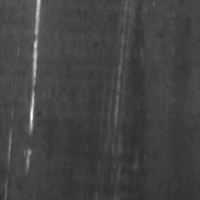

Supplement: S3 Dataset — The third typical strip steel surface defects dataset. (ZIP) [file pone.0212361.s003.zip › scratches/Sc_194.bmp]

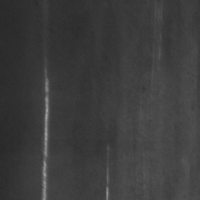

Supplement: S3 Dataset — The third typical strip steel surface defects dataset. (ZIP) [file pone.0212361.s003.zip › scratches/Sc_195.bmp]

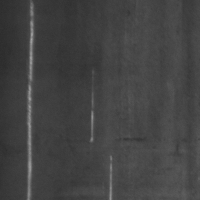

Supplement: S3 Dataset — The third typical strip steel surface defects dataset. (ZIP) [file pone.0212361.s003.zip › scratches/Sc_196.bmp]

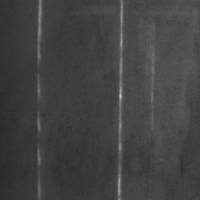

Supplement: S3 Dataset — The third typical strip steel surface defects dataset. (ZIP) [file pone.0212361.s003.zip › scratches/Sc_197.bmp]

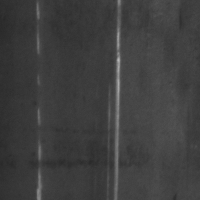

Supplement: S3 Dataset — The third typical strip steel surface defects dataset. (ZIP) [file pone.0212361.s003.zip › scratches/Sc_198.bmp]

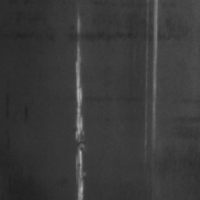

Supplement: S3 Dataset — The third typical strip steel surface defects dataset. (ZIP) [file pone.0212361.s003.zip › scratches/Sc_199.bmp]

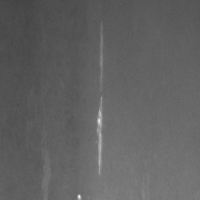

Supplement: S3 Dataset — The third typical strip steel surface defects dataset. (ZIP) [file pone.0212361.s003.zip › scratches/Sc_2.bmp]

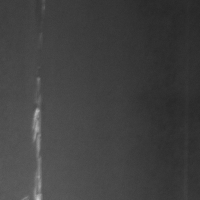

Supplement: S3 Dataset — The third typical strip steel surface defects dataset. (ZIP) [file pone.0212361.s003.zip › scratches/Sc_20.bmp]

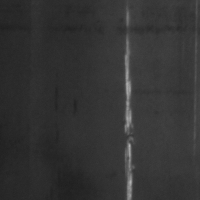

Supplement: S3 Dataset — The third typical strip steel surface defects dataset. (ZIP) [file pone.0212361.s003.zip › scratches/Sc_200.bmp]

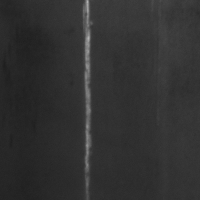

Supplement: S3 Dataset — The third typical strip steel surface defects dataset. (ZIP) [file pone.0212361.s003.zip › scratches/Sc_201.bmp]

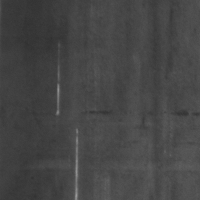

Supplement: S3 Dataset — The third typical strip steel surface defects dataset. (ZIP) [file pone.0212361.s003.zip › scratches/Sc_202.bmp]

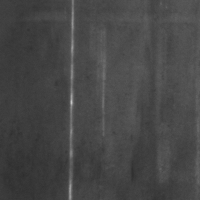

Supplement: S3 Dataset — The third typical strip steel surface defects dataset. (ZIP) [file pone.0212361.s003.zip › scratches/Sc_203.bmp]

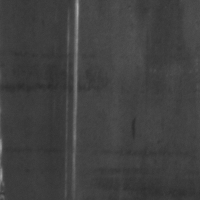

Supplement: S3 Dataset — The third typical strip steel surface defects dataset. (ZIP) [file pone.0212361.s003.zip › scratches/Sc_204.bmp]

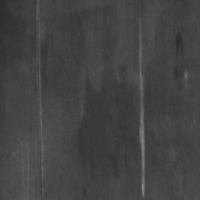

Supplement: S3 Dataset — The third typical strip steel surface defects dataset. (ZIP) [file pone.0212361.s003.zip › scratches/Sc_205.bmp]

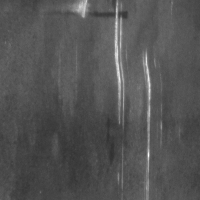

Supplement: S3 Dataset — The third typical strip steel surface defects dataset. (ZIP) [file pone.0212361.s003.zip › scratches/Sc_206.bmp]

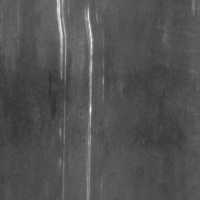

Supplement: S3 Dataset — The third typical strip steel surface defects dataset. (ZIP) [file pone.0212361.s003.zip › scratches/Sc_207.bmp]

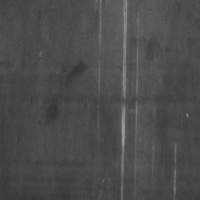

Supplement: S3 Dataset — The third typical strip steel surface defects dataset. (ZIP) [file pone.0212361.s003.zip › scratches/Sc_208.bmp]

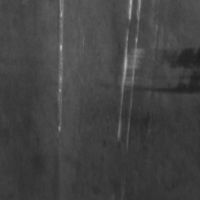

Supplement: S3 Dataset — The third typical strip steel surface defects dataset. (ZIP) [file pone.0212361.s003.zip › scratches/Sc_209.bmp]

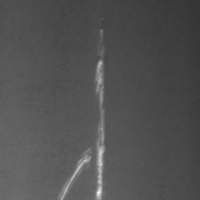

Supplement: S3 Dataset — The third typical strip steel surface defects dataset. (ZIP) [file pone.0212361.s003.zip › scratches/Sc_21.bmp]

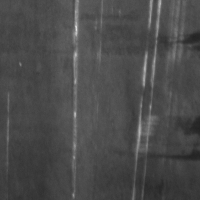

Supplement: S3 Dataset — The third typical strip steel surface defects dataset. (ZIP) [file pone.0212361.s003.zip › scratches/Sc_210.bmp]

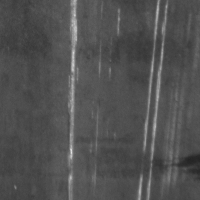

Supplement: S3 Dataset — The third typical strip steel surface defects dataset. (ZIP) [file pone.0212361.s003.zip › scratches/Sc_211.bmp]

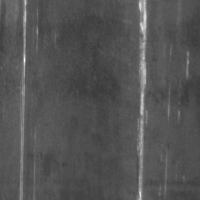

Supplement: S3 Dataset — The third typical strip steel surface defects dataset. (ZIP) [file pone.0212361.s003.zip › scratches/Sc_212.bmp]

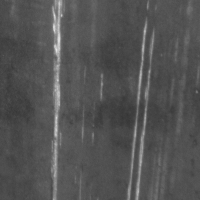

Supplement: S3 Dataset — The third typical strip steel surface defects dataset. (ZIP) [file pone.0212361.s003.zip › scratches/Sc_213.bmp]

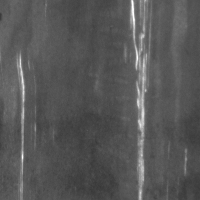

Supplement: S3 Dataset — The third typical strip steel surface defects dataset. (ZIP) [file pone.0212361.s003.zip › scratches/Sc_214.bmp]

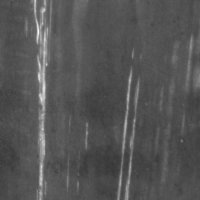

Supplement: S3 Dataset — The third typical strip steel surface defects dataset. (ZIP) [file pone.0212361.s003.zip › scratches/Sc_215.bmp]

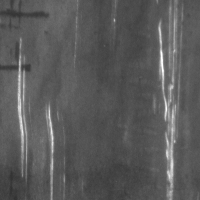

Supplement: S3 Dataset — The third typical strip steel surface defects dataset. (ZIP) [file pone.0212361.s003.zip › scratches/Sc_216.bmp]

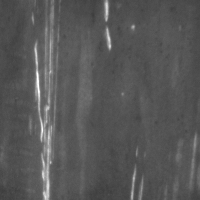

Supplement: S3 Dataset — The third typical strip steel surface defects dataset. (ZIP) [file pone.0212361.s003.zip › scratches/Sc_217.bmp]

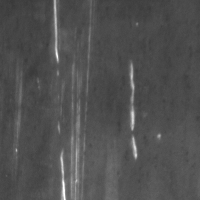

Supplement: S3 Dataset — The third typical strip steel surface defects dataset. (ZIP) [file pone.0212361.s003.zip › scratches/Sc_218.bmp]

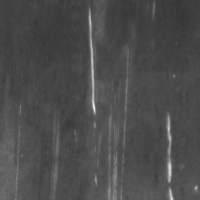

Supplement: S3 Dataset — The third typical strip steel surface defects dataset. (ZIP) [file pone.0212361.s003.zip › scratches/Sc_219.bmp]

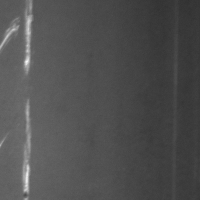

Supplement: S3 Dataset — The third typical strip steel surface defects dataset. (ZIP) [file pone.0212361.s003.zip › scratches/Sc_22.bmp]

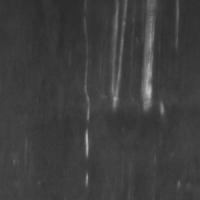

Supplement: S3 Dataset — The third typical strip steel surface defects dataset. (ZIP) [file pone.0212361.s003.zip › scratches/Sc_220.bmp]

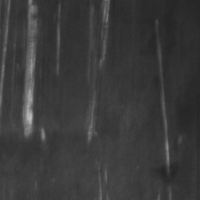

Supplement: S3 Dataset — The third typical strip steel surface defects dataset. (ZIP) [file pone.0212361.s003.zip › scratches/Sc_221.bmp]

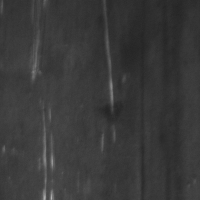

Supplement: S3 Dataset — The third typical strip steel surface defects dataset. (ZIP) [file pone.0212361.s003.zip › scratches/Sc_222.bmp]

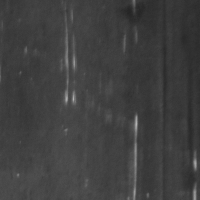

Supplement: S3 Dataset — The third typical strip steel surface defects dataset. (ZIP) [file pone.0212361.s003.zip › scratches/Sc_223.bmp]

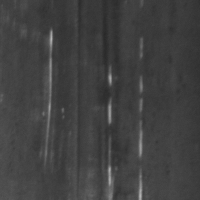

Supplement: S3 Dataset — The third typical strip steel surface defects dataset. (ZIP) [file pone.0212361.s003.zip › scratches/Sc_224.bmp]

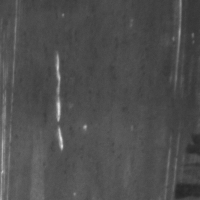

Supplement: S3 Dataset — The third typical strip steel surface defects dataset. (ZIP) [file pone.0212361.s003.zip › scratches/Sc_225.bmp]

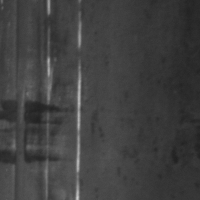

Supplement: S3 Dataset — The third typical strip steel surface defects dataset. (ZIP) [file pone.0212361.s003.zip › scratches/Sc_226.bmp]

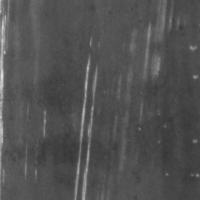

Supplement: S3 Dataset — The third typical strip steel surface defects dataset. (ZIP) [file pone.0212361.s003.zip › scratches/Sc_227.bmp]

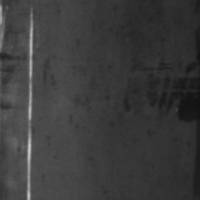

Supplement: S3 Dataset — The third typical strip steel surface defects dataset. (ZIP) [file pone.0212361.s003.zip › scratches/Sc_228.bmp]

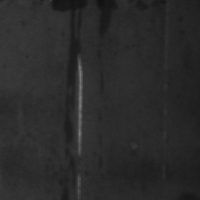

Supplement: S3 Dataset — The third typical strip steel surface defects dataset. (ZIP) [file pone.0212361.s003.zip › scratches/Sc_229.bmp]

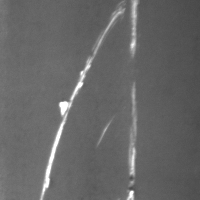

Supplement: S3 Dataset — The third typical strip steel surface defects dataset. (ZIP) [file pone.0212361.s003.zip › scratches/Sc_23.bmp]

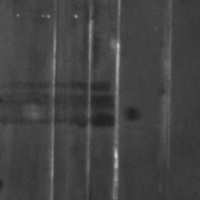

Supplement: S3 Dataset — The third typical strip steel surface defects dataset. (ZIP) [file pone.0212361.s003.zip › scratches/Sc_230.bmp]

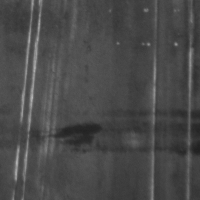

Supplement: S3 Dataset — The third typical strip steel surface defects dataset. (ZIP) [file pone.0212361.s003.zip › scratches/Sc_231.bmp]

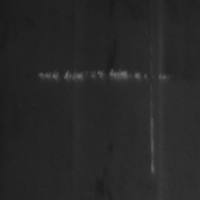

Supplement: S3 Dataset — The third typical strip steel surface defects dataset. (ZIP) [file pone.0212361.s003.zip › scratches/Sc_232.bmp]

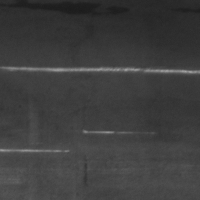

Supplement: S3 Dataset — The third typical strip steel surface defects dataset. (ZIP) [file pone.0212361.s003.zip › scratches/Sc_233.bmp]

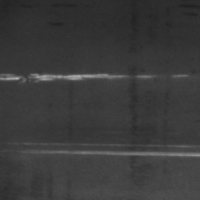

Supplement: S3 Dataset — The third typical strip steel surface defects dataset. (ZIP) [file pone.0212361.s003.zip › scratches/Sc_234.bmp]

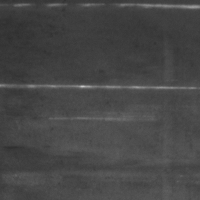

Supplement: S3 Dataset — The third typical strip steel surface defects dataset. (ZIP) [file pone.0212361.s003.zip › scratches/Sc_235.bmp]

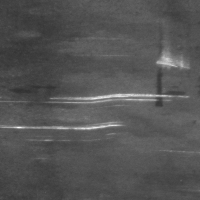

Supplement: S3 Dataset — The third typical strip steel surface defects dataset. (ZIP) [file pone.0212361.s003.zip › scratches/Sc_236.bmp]

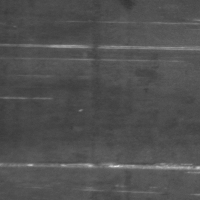

Supplement: S3 Dataset — The third typical strip steel surface defects dataset. (ZIP) [file pone.0212361.s003.zip › scratches/Sc_237.bmp]

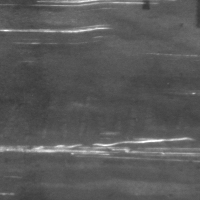

Supplement: S3 Dataset — The third typical strip steel surface defects dataset. (ZIP) [file pone.0212361.s003.zip › scratches/Sc_238.bmp]

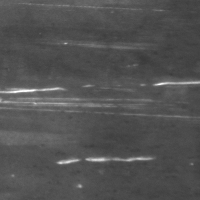

Supplement: S3 Dataset — The third typical strip steel surface defects dataset. (ZIP) [file pone.0212361.s003.zip › scratches/Sc_239.bmp]

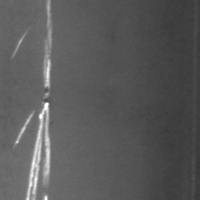

Supplement: S3 Dataset — The third typical strip steel surface defects dataset. (ZIP) [file pone.0212361.s003.zip › scratches/Sc_24.bmp]

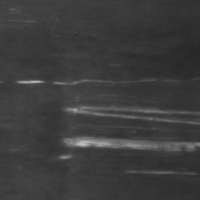

Supplement: S3 Dataset — The third typical strip steel surface defects dataset. (ZIP) [file pone.0212361.s003.zip › scratches/Sc_240.bmp]

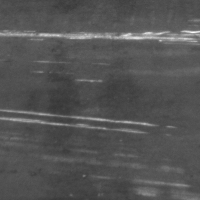

Supplement: S3 Dataset — The third typical strip steel surface defects dataset. (ZIP) [file pone.0212361.s003.zip › scratches/Sc_241.bmp]

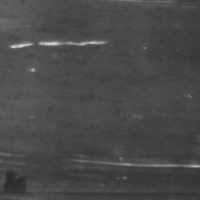

Supplement: S3 Dataset — The third typical strip steel surface defects dataset. (ZIP) [file pone.0212361.s003.zip › scratches/Sc_242.bmp]

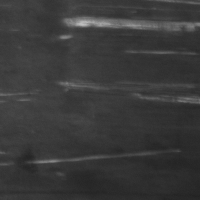

Supplement: S3 Dataset — The third typical strip steel surface defects dataset. (ZIP) [file pone.0212361.s003.zip › scratches/Sc_243.bmp]

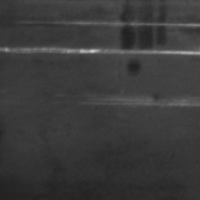

Supplement: S3 Dataset — The third typical strip steel surface defects dataset. (ZIP) [file pone.0212361.s003.zip › scratches/Sc_244.bmp]

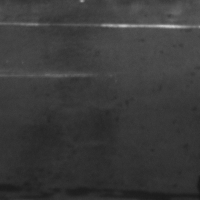

Supplement: S3 Dataset — The third typical strip steel surface defects dataset. (ZIP) [file pone.0212361.s003.zip › scratches/Sc_245.bmp]

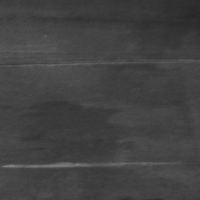

Supplement: S3 Dataset — The third typical strip steel surface defects dataset. (ZIP) [file pone.0212361.s003.zip › scratches/Sc_246.bmp]

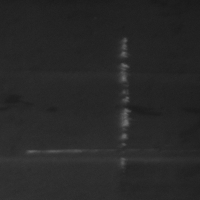

Supplement: S3 Dataset — The third typical strip steel surface defects dataset. (ZIP) [file pone.0212361.s003.zip › scratches/Sc_247.bmp]

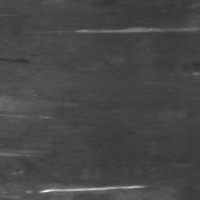

Supplement: S3 Dataset — The third typical strip steel surface defects dataset. (ZIP) [file pone.0212361.s003.zip › scratches/Sc_248.bmp]

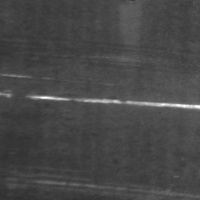

Supplement: S3 Dataset — The third typical strip steel surface defects dataset. (ZIP) [file pone.0212361.s003.zip › scratches/Sc_249.bmp]

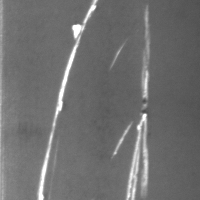

Supplement: S3 Dataset — The third typical strip steel surface defects dataset. (ZIP) [file pone.0212361.s003.zip › scratches/Sc_25.bmp]

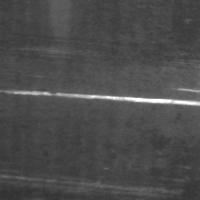

Supplement: S3 Dataset — The third typical strip steel surface defects dataset. (ZIP) [file pone.0212361.s003.zip › scratches/Sc_250.bmp]

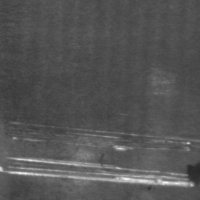

Supplement: S3 Dataset — The third typical strip steel surface defects dataset. (ZIP) [file pone.0212361.s003.zip › scratches/Sc_251.bmp]

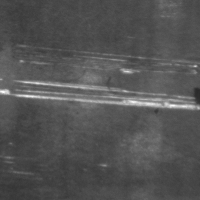

Supplement: S3 Dataset — The third typical strip steel surface defects dataset. (ZIP) [file pone.0212361.s003.zip › scratches/Sc_252.bmp]

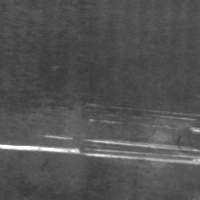

Supplement: S3 Dataset — The third typical strip steel surface defects dataset. (ZIP) [file pone.0212361.s003.zip › scratches/Sc_253.bmp]

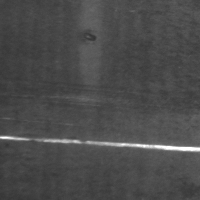

Supplement: S3 Dataset — The third typical strip steel surface defects dataset. (ZIP) [file pone.0212361.s003.zip › scratches/Sc_254.bmp]

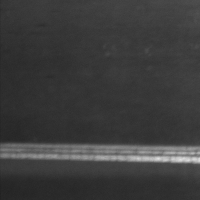

Supplement: S3 Dataset — The third typical strip steel surface defects dataset. (ZIP) [file pone.0212361.s003.zip › scratches/Sc_255.bmp]

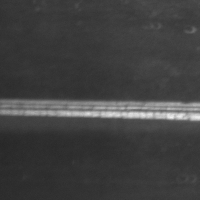

Supplement: S3 Dataset — The third typical strip steel surface defects dataset. (ZIP) [file pone.0212361.s003.zip › scratches/Sc_256.bmp]

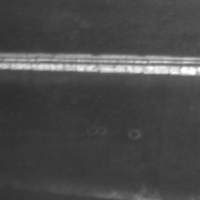

Supplement: S3 Dataset — The third typical strip steel surface defects dataset. (ZIP) [file pone.0212361.s003.zip › scratches/Sc_257.bmp]

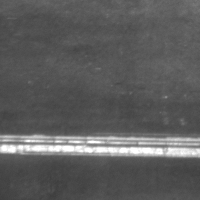

Supplement: S3 Dataset — The third typical strip steel surface defects dataset. (ZIP) [file pone.0212361.s003.zip › scratches/Sc_258.bmp]

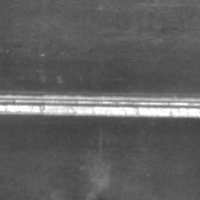

Supplement: S3 Dataset — The third typical strip steel surface defects dataset. (ZIP) [file pone.0212361.s003.zip › scratches/Sc_259.bmp]

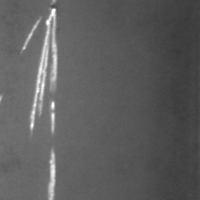

Supplement: S3 Dataset — The third typical strip steel surface defects dataset. (ZIP) [file pone.0212361.s003.zip › scratches/Sc_26.bmp]

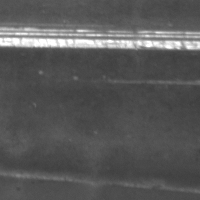

Supplement: S3 Dataset — The third typical strip steel surface defects dataset. (ZIP) [file pone.0212361.s003.zip › scratches/Sc_260.bmp]

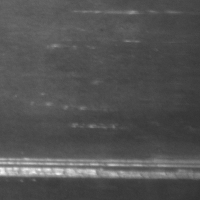

Supplement: S3 Dataset — The third typical strip steel surface defects dataset. (ZIP) [file pone.0212361.s003.zip › scratches/Sc_261.bmp]

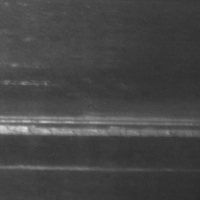

Supplement: S3 Dataset — The third typical strip steel surface defects dataset. (ZIP) [file pone.0212361.s003.zip › scratches/Sc_262.bmp]

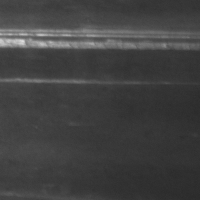

Supplement: S3 Dataset — The third typical strip steel surface defects dataset. (ZIP) [file pone.0212361.s003.zip › scratches/Sc_263.bmp]

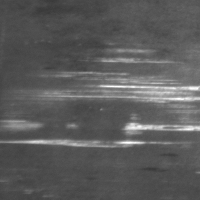

Supplement: S3 Dataset — The third typical strip steel surface defects dataset. (ZIP) [file pone.0212361.s003.zip › scratches/Sc_264.bmp]

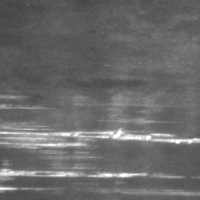

Supplement: S3 Dataset — The third typical strip steel surface defects dataset. (ZIP) [file pone.0212361.s003.zip › scratches/Sc_265.bmp]

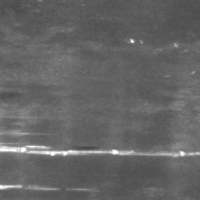

Supplement: S3 Dataset — The third typical strip steel surface defects dataset. (ZIP) [file pone.0212361.s003.zip › scratches/Sc_266.bmp]

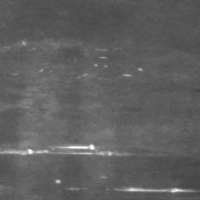

Supplement: S3 Dataset — The third typical strip steel surface defects dataset. (ZIP) [file pone.0212361.s003.zip › scratches/Sc_267.bmp]

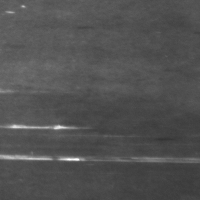

Supplement: S3 Dataset — The third typical strip steel surface defects dataset. (ZIP) [file pone.0212361.s003.zip › scratches/Sc_268.bmp]

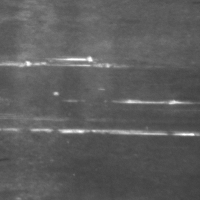

Supplement: S3 Dataset — The third typical strip steel surface defects dataset. (ZIP) [file pone.0212361.s003.zip › scratches/Sc_269.bmp]

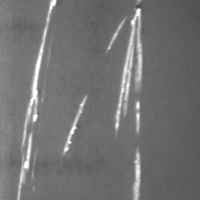

Supplement: S3 Dataset — The third typical strip steel surface defects dataset. (ZIP) [file pone.0212361.s003.zip › scratches/Sc_27.bmp]

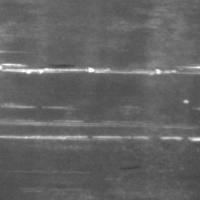

Supplement: S3 Dataset — The third typical strip steel surface defects dataset. (ZIP) [file pone.0212361.s003.zip › scratches/Sc_270.bmp]

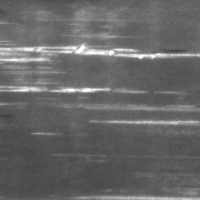

Supplement: S3 Dataset — The third typical strip steel surface defects dataset. (ZIP) [file pone.0212361.s003.zip › scratches/Sc_271.bmp]

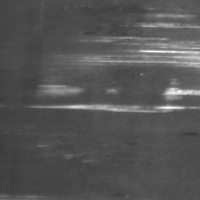

Supplement: S3 Dataset — The third typical strip steel surface defects dataset. (ZIP) [file pone.0212361.s003.zip › scratches/Sc_272.bmp]

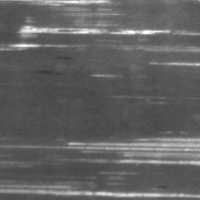

Supplement: S3 Dataset — The third typical strip steel surface defects dataset. (ZIP) [file pone.0212361.s003.zip › scratches/Sc_273.bmp]

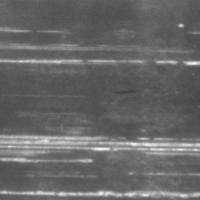

Supplement: S3 Dataset — The third typical strip steel surface defects dataset. (ZIP) [file pone.0212361.s003.zip › scratches/Sc_274.bmp]

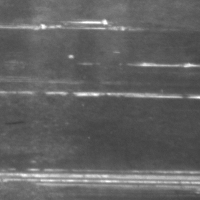

Supplement: S3 Dataset — The third typical strip steel surface defects dataset. (ZIP) [file pone.0212361.s003.zip › scratches/Sc_275.bmp]

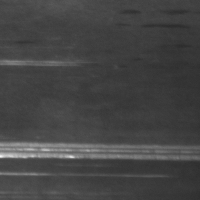

Supplement: S3 Dataset — The third typical strip steel surface defects dataset. (ZIP) [file pone.0212361.s003.zip › scratches/Sc_276.bmp]

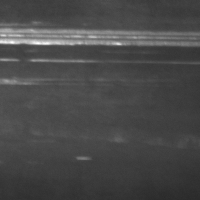

Supplement: S3 Dataset — The third typical strip steel surface defects dataset. (ZIP) [file pone.0212361.s003.zip › scratches/Sc_277.bmp]

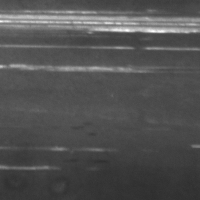

Supplement: S3 Dataset — The third typical strip steel surface defects dataset. (ZIP) [file pone.0212361.s003.zip › scratches/Sc_278.bmp]

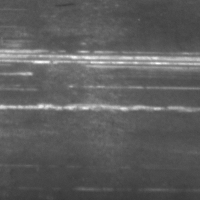

Supplement: S3 Dataset — The third typical strip steel surface defects dataset. (ZIP) [file pone.0212361.s003.zip › scratches/Sc_279.bmp]
