# Supplementary figures and images for: Support vector machine with quantile hyper-spheres for pattern classification (part 3 of 6)
Source: PLoS One. 2019 Feb 15;14(2):e0212361. doi: 10.1371/journal.pone.0212361 (PMC6377146; doi:10.1371/journal.pone.0212361)

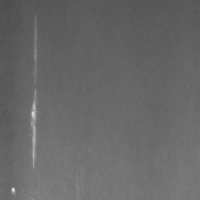

Supplement: S3 Dataset — The third typical strip steel surface defects dataset. (ZIP) [file pone.0212361.s003.zip › scratches/Sc_1.bmp]

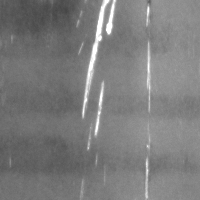

Supplement: S3 Dataset — The third typical strip steel surface defects dataset. (ZIP) [file pone.0212361.s003.zip › scratches/Sc_10.bmp]

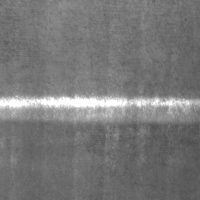

Supplement: S3 Dataset — The third typical strip steel surface defects dataset. (ZIP) [file pone.0212361.s003.zip › scratches/Sc_100.bmp]

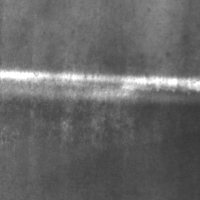

Supplement: S3 Dataset — The third typical strip steel surface defects dataset. (ZIP) [file pone.0212361.s003.zip › scratches/Sc_101.bmp]

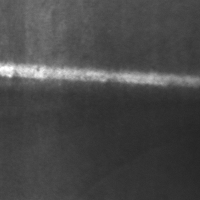

Supplement: S3 Dataset — The third typical strip steel surface defects dataset. (ZIP) [file pone.0212361.s003.zip › scratches/Sc_102.bmp]

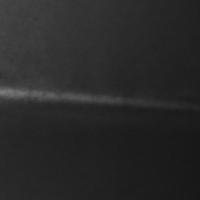

Supplement: S3 Dataset — The third typical strip steel surface defects dataset. (ZIP) [file pone.0212361.s003.zip › scratches/Sc_103.bmp]

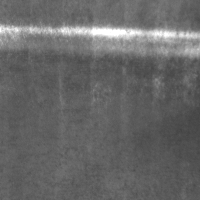

Supplement: S3 Dataset — The third typical strip steel surface defects dataset. (ZIP) [file pone.0212361.s003.zip › scratches/Sc_104.bmp]

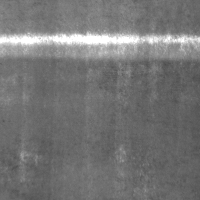

Supplement: S3 Dataset — The third typical strip steel surface defects dataset. (ZIP) [file pone.0212361.s003.zip › scratches/Sc_105.bmp]

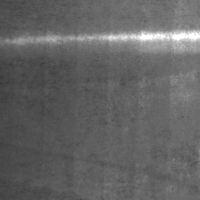

Supplement: S3 Dataset — The third typical strip steel surface defects dataset. (ZIP) [file pone.0212361.s003.zip › scratches/Sc_106.bmp]

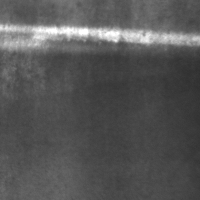

Supplement: S3 Dataset — The third typical strip steel surface defects dataset. (ZIP) [file pone.0212361.s003.zip › scratches/Sc_107.bmp]

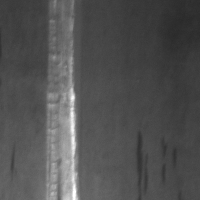

Supplement: S3 Dataset — The third typical strip steel surface defects dataset. (ZIP) [file pone.0212361.s003.zip › scratches/Sc_108.bmp]

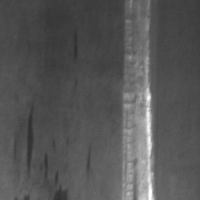

Supplement: S3 Dataset — The third typical strip steel surface defects dataset. (ZIP) [file pone.0212361.s003.zip › scratches/Sc_109.bmp]

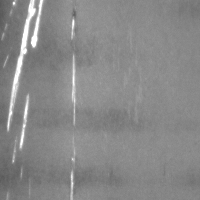

Supplement: S3 Dataset — The third typical strip steel surface defects dataset. (ZIP) [file pone.0212361.s003.zip › scratches/Sc_11.bmp]

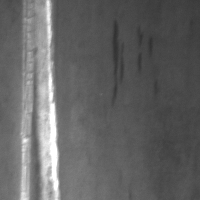

Supplement: S3 Dataset — The third typical strip steel surface defects dataset. (ZIP) [file pone.0212361.s003.zip › scratches/Sc_110.bmp]

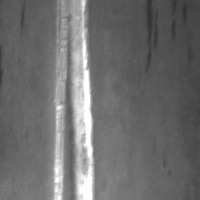

Supplement: S3 Dataset — The third typical strip steel surface defects dataset. (ZIP) [file pone.0212361.s003.zip › scratches/Sc_111.bmp]

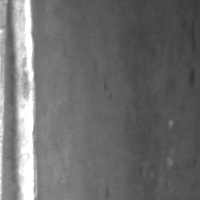

Supplement: S3 Dataset — The third typical strip steel surface defects dataset. (ZIP) [file pone.0212361.s003.zip › scratches/Sc_112.bmp]

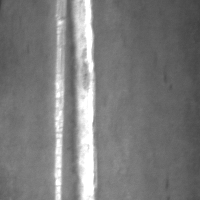

Supplement: S3 Dataset — The third typical strip steel surface defects dataset. (ZIP) [file pone.0212361.s003.zip › scratches/Sc_113.bmp]

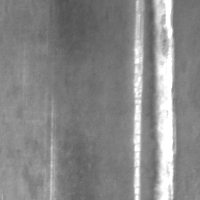

Supplement: S3 Dataset — The third typical strip steel surface defects dataset. (ZIP) [file pone.0212361.s003.zip › scratches/Sc_114.bmp]

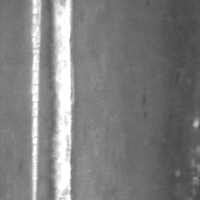

Supplement: S3 Dataset — The third typical strip steel surface defects dataset. (ZIP) [file pone.0212361.s003.zip › scratches/Sc_115.bmp]

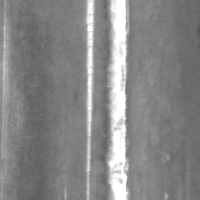

Supplement: S3 Dataset — The third typical strip steel surface defects dataset. (ZIP) [file pone.0212361.s003.zip › scratches/Sc_116.bmp]

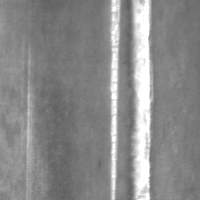

Supplement: S3 Dataset — The third typical strip steel surface defects dataset. (ZIP) [file pone.0212361.s003.zip › scratches/Sc_117.bmp]

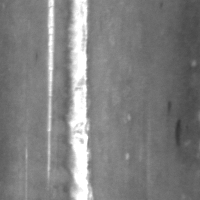

Supplement: S3 Dataset — The third typical strip steel surface defects dataset. (ZIP) [file pone.0212361.s003.zip › scratches/Sc_118.bmp]

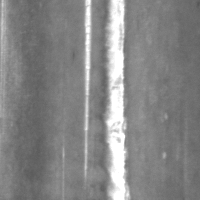

Supplement: S3 Dataset — The third typical strip steel surface defects dataset. (ZIP) [file pone.0212361.s003.zip › scratches/Sc_119.bmp]

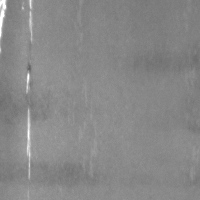

Supplement: S3 Dataset — The third typical strip steel surface defects dataset. (ZIP) [file pone.0212361.s003.zip › scratches/Sc_12.bmp]

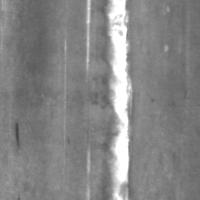

Supplement: S3 Dataset — The third typical strip steel surface defects dataset. (ZIP) [file pone.0212361.s003.zip › scratches/Sc_120.bmp]

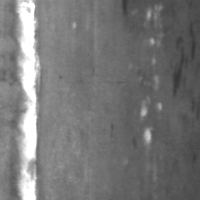

Supplement: S3 Dataset — The third typical strip steel surface defects dataset. (ZIP) [file pone.0212361.s003.zip › scratches/Sc_121.bmp]

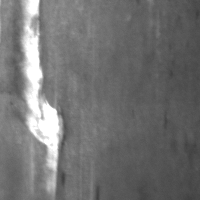

Supplement: S3 Dataset — The third typical strip steel surface defects dataset. (ZIP) [file pone.0212361.s003.zip › scratches/Sc_122.bmp]

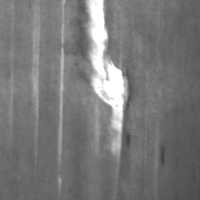

Supplement: S3 Dataset — The third typical strip steel surface defects dataset. (ZIP) [file pone.0212361.s003.zip › scratches/Sc_123.bmp]

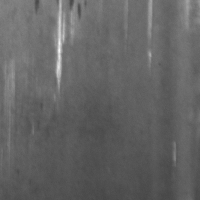

Supplement: S3 Dataset — The third typical strip steel surface defects dataset. (ZIP) [file pone.0212361.s003.zip › scratches/Sc_124.bmp]

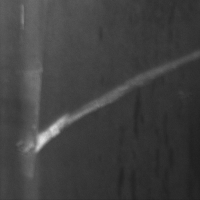

Supplement: S3 Dataset — The third typical strip steel surface defects dataset. (ZIP) [file pone.0212361.s003.zip › scratches/Sc_125.bmp]

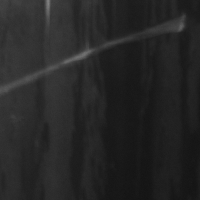

Supplement: S3 Dataset — The third typical strip steel surface defects dataset. (ZIP) [file pone.0212361.s003.zip › scratches/Sc_126.bmp]

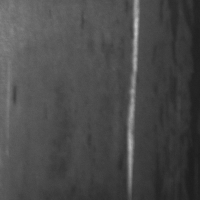

Supplement: S3 Dataset — The third typical strip steel surface defects dataset. (ZIP) [file pone.0212361.s003.zip › scratches/Sc_127.bmp]

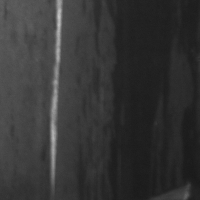

Supplement: S3 Dataset — The third typical strip steel surface defects dataset. (ZIP) [file pone.0212361.s003.zip › scratches/Sc_128.bmp]

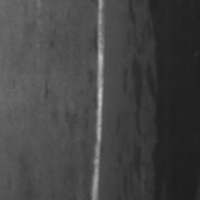

Supplement: S3 Dataset — The third typical strip steel surface defects dataset. (ZIP) [file pone.0212361.s003.zip › scratches/Sc_129.bmp]

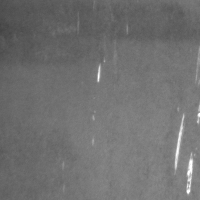

Supplement: S3 Dataset — The third typical strip steel surface defects dataset. (ZIP) [file pone.0212361.s003.zip › scratches/Sc_13.bmp]

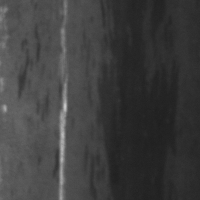

Supplement: S3 Dataset — The third typical strip steel surface defects dataset. (ZIP) [file pone.0212361.s003.zip › scratches/Sc_130.bmp]

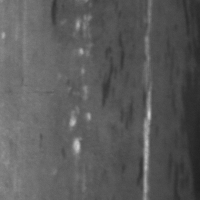

Supplement: S3 Dataset — The third typical strip steel surface defects dataset. (ZIP) [file pone.0212361.s003.zip › scratches/Sc_131.bmp]

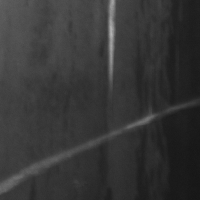

Supplement: S3 Dataset — The third typical strip steel surface defects dataset. (ZIP) [file pone.0212361.s003.zip › scratches/Sc_132.bmp]

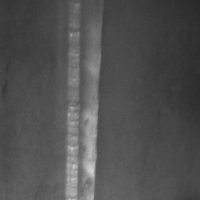

Supplement: S3 Dataset — The third typical strip steel surface defects dataset. (ZIP) [file pone.0212361.s003.zip › scratches/Sc_133.bmp]

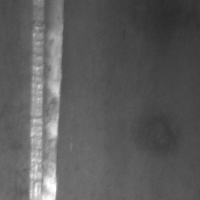

Supplement: S3 Dataset — The third typical strip steel surface defects dataset. (ZIP) [file pone.0212361.s003.zip › scratches/Sc_134.bmp]

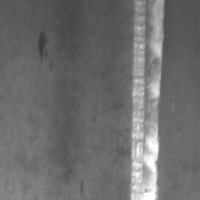

Supplement: S3 Dataset — The third typical strip steel surface defects dataset. (ZIP) [file pone.0212361.s003.zip › scratches/Sc_135.bmp]

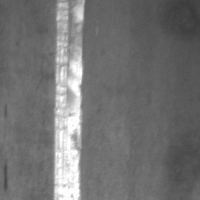

Supplement: S3 Dataset — The third typical strip steel surface defects dataset. (ZIP) [file pone.0212361.s003.zip › scratches/Sc_136.bmp]

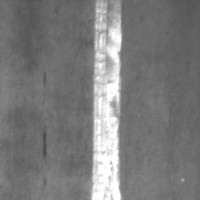

Supplement: S3 Dataset — The third typical strip steel surface defects dataset. (ZIP) [file pone.0212361.s003.zip › scratches/Sc_137.bmp]

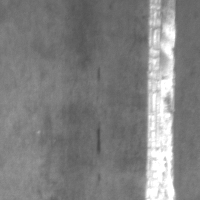

Supplement: S3 Dataset — The third typical strip steel surface defects dataset. (ZIP) [file pone.0212361.s003.zip › scratches/Sc_138.bmp]

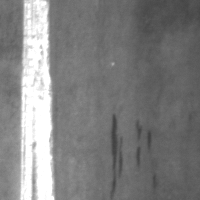

Supplement: S3 Dataset — The third typical strip steel surface defects dataset. (ZIP) [file pone.0212361.s003.zip › scratches/Sc_139.bmp]

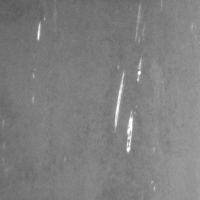

Supplement: S3 Dataset — The third typical strip steel surface defects dataset. (ZIP) [file pone.0212361.s003.zip › scratches/Sc_14.bmp]

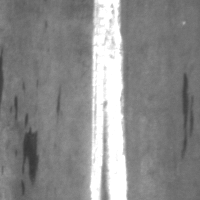

Supplement: S3 Dataset — The third typical strip steel surface defects dataset. (ZIP) [file pone.0212361.s003.zip › scratches/Sc_140.bmp]

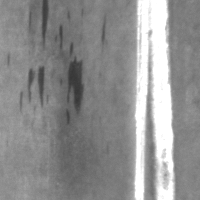

Supplement: S3 Dataset — The third typical strip steel surface defects dataset. (ZIP) [file pone.0212361.s003.zip › scratches/Sc_141.bmp]

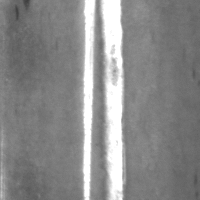

Supplement: S3 Dataset — The third typical strip steel surface defects dataset. (ZIP) [file pone.0212361.s003.zip › scratches/Sc_142.bmp]

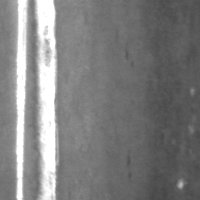

Supplement: S3 Dataset — The third typical strip steel surface defects dataset. (ZIP) [file pone.0212361.s003.zip › scratches/Sc_143.bmp]

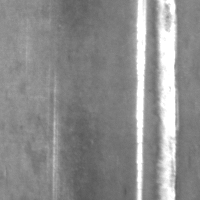

Supplement: S3 Dataset — The third typical strip steel surface defects dataset. (ZIP) [file pone.0212361.s003.zip › scratches/Sc_144.bmp]

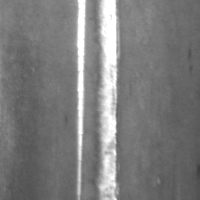

Supplement: S3 Dataset — The third typical strip steel surface defects dataset. (ZIP) [file pone.0212361.s003.zip › scratches/Sc_145.bmp]

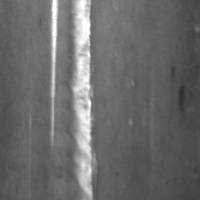

Supplement: S3 Dataset — The third typical strip steel surface defects dataset. (ZIP) [file pone.0212361.s003.zip › scratches/Sc_146.bmp]

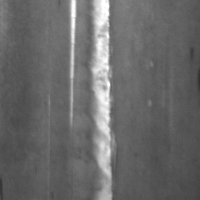

Supplement: S3 Dataset — The third typical strip steel surface defects dataset. (ZIP) [file pone.0212361.s003.zip › scratches/Sc_147.bmp]

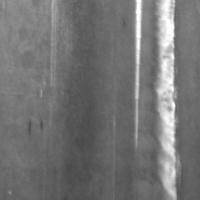

Supplement: S3 Dataset — The third typical strip steel surface defects dataset. (ZIP) [file pone.0212361.s003.zip › scratches/Sc_148.bmp]

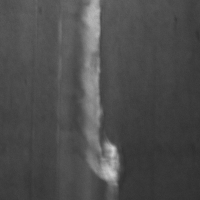

Supplement: S3 Dataset — The third typical strip steel surface defects dataset. (ZIP) [file pone.0212361.s003.zip › scratches/Sc_149.bmp]

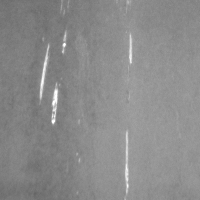

Supplement: S3 Dataset — The third typical strip steel surface defects dataset. (ZIP) [file pone.0212361.s003.zip › scratches/Sc_15.bmp]

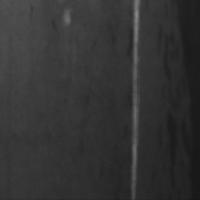

Supplement: S3 Dataset — The third typical strip steel surface defects dataset. (ZIP) [file pone.0212361.s003.zip › scratches/Sc_150.bmp]

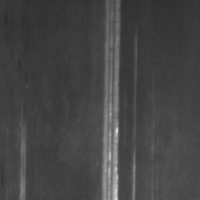

Supplement: S3 Dataset — The third typical strip steel surface defects dataset. (ZIP) [file pone.0212361.s003.zip › scratches/Sc_151.bmp]

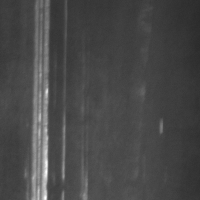

Supplement: S3 Dataset — The third typical strip steel surface defects dataset. (ZIP) [file pone.0212361.s003.zip › scratches/Sc_152.bmp]

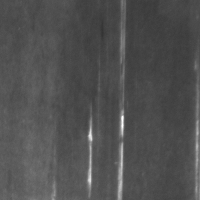

Supplement: S3 Dataset — The third typical strip steel surface defects dataset. (ZIP) [file pone.0212361.s003.zip › scratches/Sc_153.bmp]

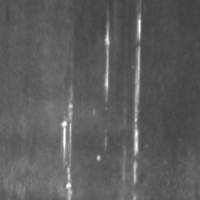

Supplement: S3 Dataset — The third typical strip steel surface defects dataset. (ZIP) [file pone.0212361.s003.zip › scratches/Sc_154.bmp]

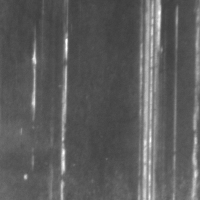

Supplement: S3 Dataset — The third typical strip steel surface defects dataset. (ZIP) [file pone.0212361.s003.zip › scratches/Sc_155.bmp]

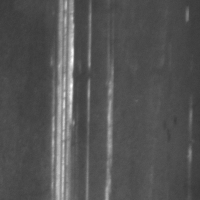

Supplement: S3 Dataset — The third typical strip steel surface defects dataset. (ZIP) [file pone.0212361.s003.zip › scratches/Sc_156.bmp]

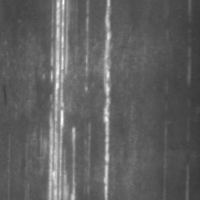

Supplement: S3 Dataset — The third typical strip steel surface defects dataset. (ZIP) [file pone.0212361.s003.zip › scratches/Sc_157.bmp]

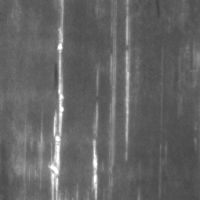

Supplement: S3 Dataset — The third typical strip steel surface defects dataset. (ZIP) [file pone.0212361.s003.zip › scratches/Sc_158.bmp]

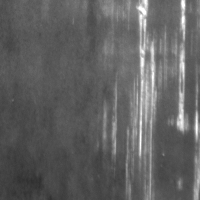

Supplement: S3 Dataset — The third typical strip steel surface defects dataset. (ZIP) [file pone.0212361.s003.zip › scratches/Sc_159.bmp]

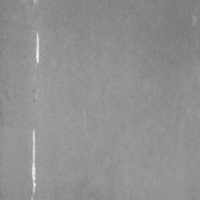

Supplement: S3 Dataset — The third typical strip steel surface defects dataset. (ZIP) [file pone.0212361.s003.zip › scratches/Sc_16.bmp]

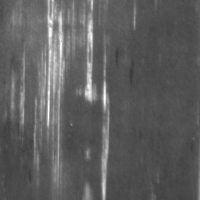

Supplement: S3 Dataset — The third typical strip steel surface defects dataset. (ZIP) [file pone.0212361.s003.zip › scratches/Sc_160.bmp]

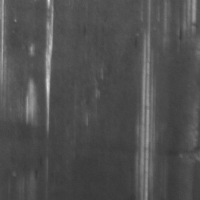

Supplement: S3 Dataset — The third typical strip steel surface defects dataset. (ZIP) [file pone.0212361.s003.zip › scratches/Sc_161.bmp]

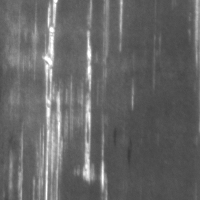

Supplement: S3 Dataset — The third typical strip steel surface defects dataset. (ZIP) [file pone.0212361.s003.zip › scratches/Sc_162.bmp]

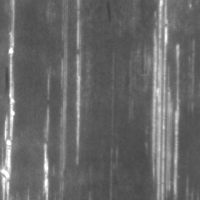

Supplement: S3 Dataset — The third typical strip steel surface defects dataset. (ZIP) [file pone.0212361.s003.zip › scratches/Sc_163.bmp]

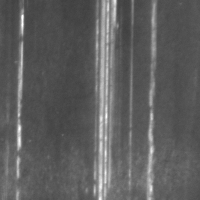

Supplement: S3 Dataset — The third typical strip steel surface defects dataset. (ZIP) [file pone.0212361.s003.zip › scratches/Sc_164.bmp]

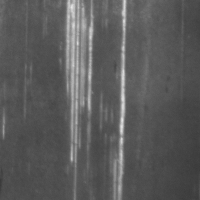

Supplement: S3 Dataset — The third typical strip steel surface defects dataset. (ZIP) [file pone.0212361.s003.zip › scratches/Sc_165.bmp]

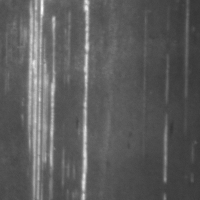

Supplement: S3 Dataset — The third typical strip steel surface defects dataset. (ZIP) [file pone.0212361.s003.zip › scratches/Sc_166.bmp]

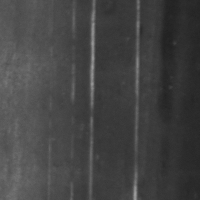

Supplement: S3 Dataset — The third typical strip steel surface defects dataset. (ZIP) [file pone.0212361.s003.zip › scratches/Sc_167.bmp]

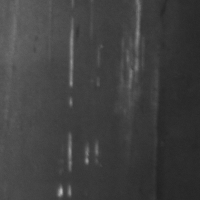

Supplement: S3 Dataset — The third typical strip steel surface defects dataset. (ZIP) [file pone.0212361.s003.zip › scratches/Sc_168.bmp]

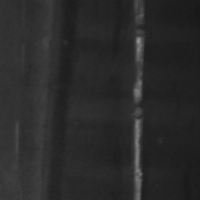

Supplement: S3 Dataset — The third typical strip steel surface defects dataset. (ZIP) [file pone.0212361.s003.zip › scratches/Sc_169.bmp]

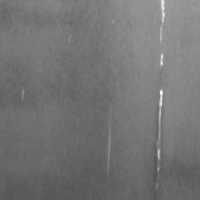

Supplement: S3 Dataset — The third typical strip steel surface defects dataset. (ZIP) [file pone.0212361.s003.zip › scratches/Sc_17.bmp]

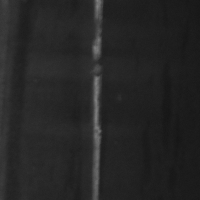

Supplement: S3 Dataset — The third typical strip steel surface defects dataset. (ZIP) [file pone.0212361.s003.zip › scratches/Sc_170.bmp]

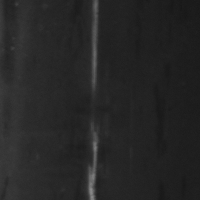

Supplement: S3 Dataset — The third typical strip steel surface defects dataset. (ZIP) [file pone.0212361.s003.zip › scratches/Sc_171.bmp]

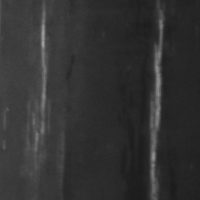

Supplement: S3 Dataset — The third typical strip steel surface defects dataset. (ZIP) [file pone.0212361.s003.zip › scratches/Sc_172.bmp]

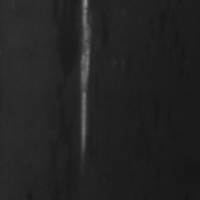

Supplement: S3 Dataset — The third typical strip steel surface defects dataset. (ZIP) [file pone.0212361.s003.zip › scratches/Sc_173.bmp]

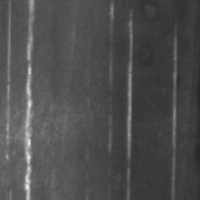

Supplement: S3 Dataset — The third typical strip steel surface defects dataset. (ZIP) [file pone.0212361.s003.zip › scratches/Sc_174.bmp]

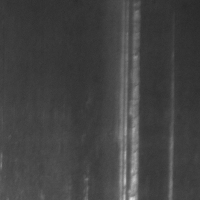

Supplement: S3 Dataset — The third typical strip steel surface defects dataset. (ZIP) [file pone.0212361.s003.zip › scratches/Sc_175.bmp]

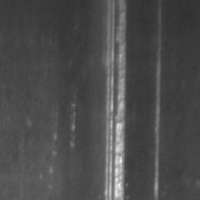

Supplement: S3 Dataset — The third typical strip steel surface defects dataset. (ZIP) [file pone.0212361.s003.zip › scratches/Sc_176.bmp]

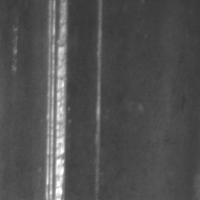

Supplement: S3 Dataset — The third typical strip steel surface defects dataset. (ZIP) [file pone.0212361.s003.zip › scratches/Sc_177.bmp]

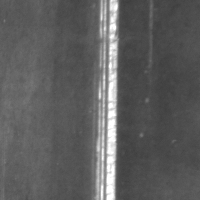

Supplement: S3 Dataset — The third typical strip steel surface defects dataset. (ZIP) [file pone.0212361.s003.zip › scratches/Sc_178.bmp]

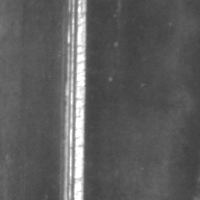

Supplement: S3 Dataset — The third typical strip steel surface defects dataset. (ZIP) [file pone.0212361.s003.zip › scratches/Sc_179.bmp]

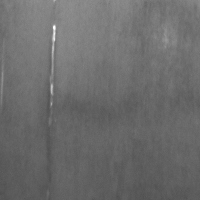

Supplement: S3 Dataset — The third typical strip steel surface defects dataset. (ZIP) [file pone.0212361.s003.zip › scratches/Sc_18.bmp]

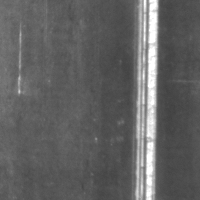

Supplement: S3 Dataset — The third typical strip steel surface defects dataset. (ZIP) [file pone.0212361.s003.zip › scratches/Sc_180.bmp]

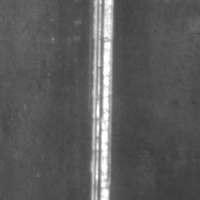

Supplement: S3 Dataset — The third typical strip steel surface defects dataset. (ZIP) [file pone.0212361.s003.zip › scratches/Sc_181.bmp]

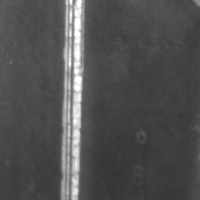

Supplement: S3 Dataset — The third typical strip steel surface defects dataset. (ZIP) [file pone.0212361.s003.zip › scratches/Sc_182.bmp]

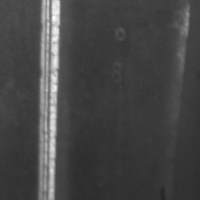

Supplement: S3 Dataset — The third typical strip steel surface defects dataset. (ZIP) [file pone.0212361.s003.zip › scratches/Sc_183.bmp]

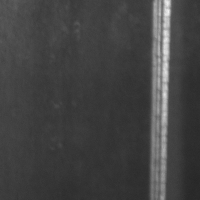

Supplement: S3 Dataset — The third typical strip steel surface defects dataset. (ZIP) [file pone.0212361.s003.zip › scratches/Sc_184.bmp]

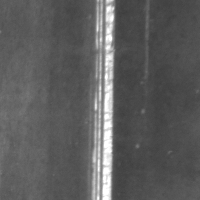

Supplement: S3 Dataset — The third typical strip steel surface defects dataset. (ZIP) [file pone.0212361.s003.zip › scratches/Sc_185.bmp]

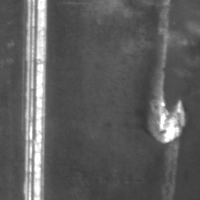

Supplement: S3 Dataset — The third typical strip steel surface defects dataset. (ZIP) [file pone.0212361.s003.zip › scratches/Sc_186.bmp]

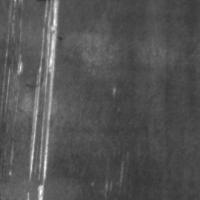

Supplement: S3 Dataset — The third typical strip steel surface defects dataset. (ZIP) [file pone.0212361.s003.zip › scratches/Sc_187.bmp]

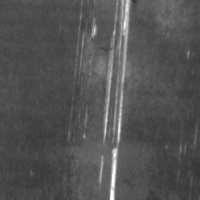

Supplement: S3 Dataset — The third typical strip steel surface defects dataset. (ZIP) [file pone.0212361.s003.zip › scratches/Sc_188.bmp]

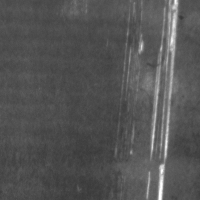

Supplement: S3 Dataset — The third typical strip steel surface defects dataset. (ZIP) [file pone.0212361.s003.zip › scratches/Sc_189.bmp]
