# Supplementary figures and images for: Support vector machine with quantile hyper-spheres for pattern classification (part 6 of 6)
Source: PLoS One. 2019 Feb 15;14(2):e0212361. doi: 10.1371/journal.pone.0212361 (PMC6377146; doi:10.1371/journal.pone.0212361)

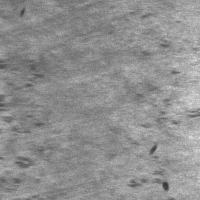

Supplement: S4 Dataset — The fourth typical strip steel surface defects dataset. (ZIP) [file pone.0212361.s004.zip › scale/RS_19.bmp]

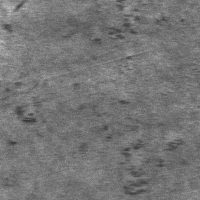

Supplement: S4 Dataset — The fourth typical strip steel surface defects dataset. (ZIP) [file pone.0212361.s004.zip › scale/RS_190.bmp]

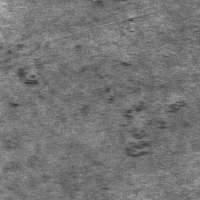

Supplement: S4 Dataset — The fourth typical strip steel surface defects dataset. (ZIP) [file pone.0212361.s004.zip › scale/RS_191.bmp]

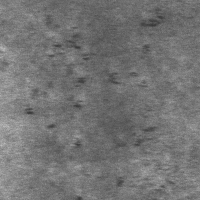

Supplement: S4 Dataset — The fourth typical strip steel surface defects dataset. (ZIP) [file pone.0212361.s004.zip › scale/RS_192.bmp]

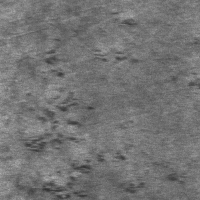

Supplement: S4 Dataset — The fourth typical strip steel surface defects dataset. (ZIP) [file pone.0212361.s004.zip › scale/RS_193.bmp]

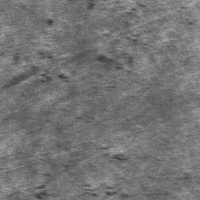

Supplement: S4 Dataset — The fourth typical strip steel surface defects dataset. (ZIP) [file pone.0212361.s004.zip › scale/RS_194.bmp]

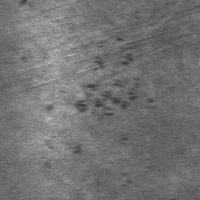

Supplement: S4 Dataset — The fourth typical strip steel surface defects dataset. (ZIP) [file pone.0212361.s004.zip › scale/RS_195.bmp]

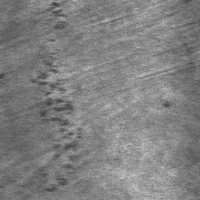

Supplement: S4 Dataset — The fourth typical strip steel surface defects dataset. (ZIP) [file pone.0212361.s004.zip › scale/RS_196.bmp]

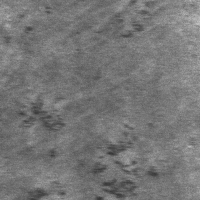

Supplement: S4 Dataset — The fourth typical strip steel surface defects dataset. (ZIP) [file pone.0212361.s004.zip › scale/RS_197.bmp]

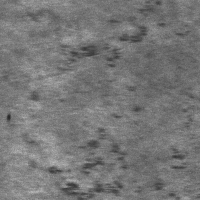

Supplement: S4 Dataset — The fourth typical strip steel surface defects dataset. (ZIP) [file pone.0212361.s004.zip › scale/RS_198.bmp]

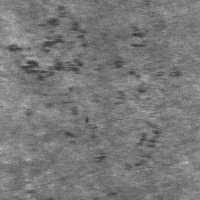

Supplement: S4 Dataset — The fourth typical strip steel surface defects dataset. (ZIP) [file pone.0212361.s004.zip › scale/RS_199.bmp]

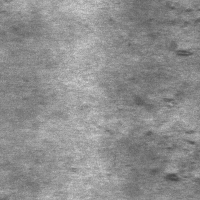

Supplement: S4 Dataset — The fourth typical strip steel surface defects dataset. (ZIP) [file pone.0212361.s004.zip › scale/RS_2.bmp]

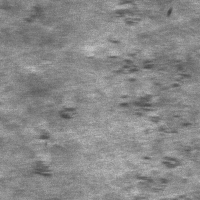

Supplement: S4 Dataset — The fourth typical strip steel surface defects dataset. (ZIP) [file pone.0212361.s004.zip › scale/RS_20.bmp]

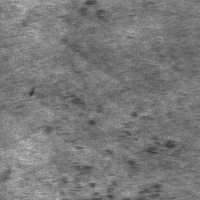

Supplement: S4 Dataset — The fourth typical strip steel surface defects dataset. (ZIP) [file pone.0212361.s004.zip › scale/RS_200.bmp]

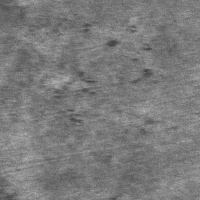

Supplement: S4 Dataset — The fourth typical strip steel surface defects dataset. (ZIP) [file pone.0212361.s004.zip › scale/RS_201.bmp]

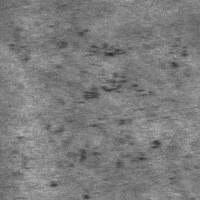

Supplement: S4 Dataset — The fourth typical strip steel surface defects dataset. (ZIP) [file pone.0212361.s004.zip › scale/RS_202.bmp]

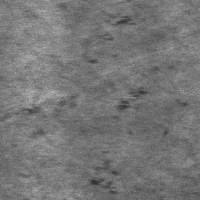

Supplement: S4 Dataset — The fourth typical strip steel surface defects dataset. (ZIP) [file pone.0212361.s004.zip › scale/RS_203.bmp]

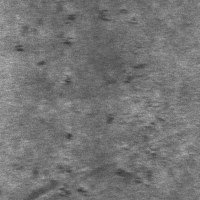

Supplement: S4 Dataset — The fourth typical strip steel surface defects dataset. (ZIP) [file pone.0212361.s004.zip › scale/RS_204.bmp]

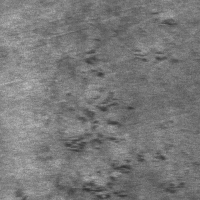

Supplement: S4 Dataset — The fourth typical strip steel surface defects dataset. (ZIP) [file pone.0212361.s004.zip › scale/RS_205.bmp]

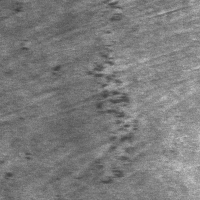

Supplement: S4 Dataset — The fourth typical strip steel surface defects dataset. (ZIP) [file pone.0212361.s004.zip › scale/RS_206.bmp]

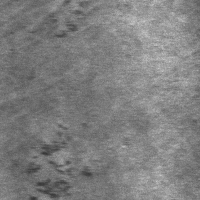

Supplement: S4 Dataset — The fourth typical strip steel surface defects dataset. (ZIP) [file pone.0212361.s004.zip › scale/RS_207.bmp]

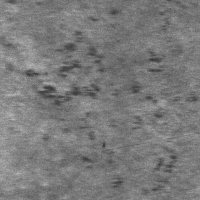

Supplement: S4 Dataset — The fourth typical strip steel surface defects dataset. (ZIP) [file pone.0212361.s004.zip › scale/RS_208.bmp]

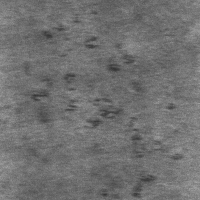

Supplement: S4 Dataset — The fourth typical strip steel surface defects dataset. (ZIP) [file pone.0212361.s004.zip › scale/RS_209.bmp]

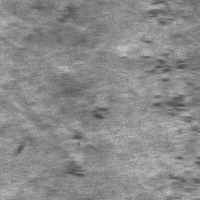

Supplement: S4 Dataset — The fourth typical strip steel surface defects dataset. (ZIP) [file pone.0212361.s004.zip › scale/RS_21.bmp]

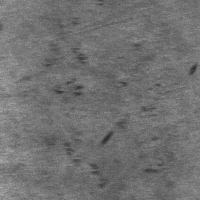

Supplement: S4 Dataset — The fourth typical strip steel surface defects dataset. (ZIP) [file pone.0212361.s004.zip › scale/RS_210.bmp]

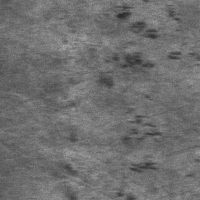

Supplement: S4 Dataset — The fourth typical strip steel surface defects dataset. (ZIP) [file pone.0212361.s004.zip › scale/RS_211.bmp]

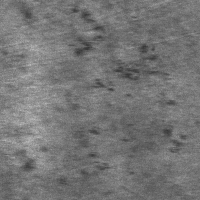

Supplement: S4 Dataset — The fourth typical strip steel surface defects dataset. (ZIP) [file pone.0212361.s004.zip › scale/RS_212.bmp]

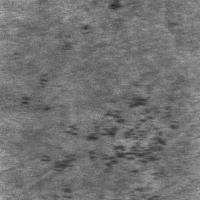

Supplement: S4 Dataset — The fourth typical strip steel surface defects dataset. (ZIP) [file pone.0212361.s004.zip › scale/RS_213.bmp]

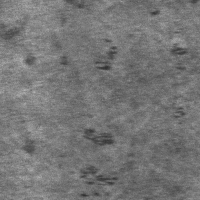

Supplement: S4 Dataset — The fourth typical strip steel surface defects dataset. (ZIP) [file pone.0212361.s004.zip › scale/RS_214.bmp]

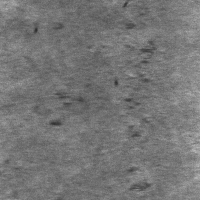

Supplement: S4 Dataset — The fourth typical strip steel surface defects dataset. (ZIP) [file pone.0212361.s004.zip › scale/RS_215.bmp]

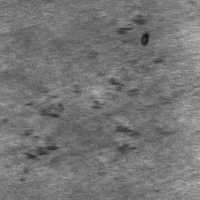

Supplement: S4 Dataset — The fourth typical strip steel surface defects dataset. (ZIP) [file pone.0212361.s004.zip › scale/RS_216.bmp]

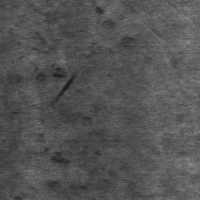

Supplement: S4 Dataset — The fourth typical strip steel surface defects dataset. (ZIP) [file pone.0212361.s004.zip › scale/RS_217.bmp]

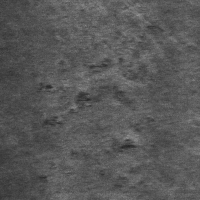

Supplement: S4 Dataset — The fourth typical strip steel surface defects dataset. (ZIP) [file pone.0212361.s004.zip › scale/RS_218.bmp]

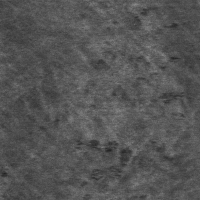

Supplement: S4 Dataset — The fourth typical strip steel surface defects dataset. (ZIP) [file pone.0212361.s004.zip › scale/RS_219.bmp]

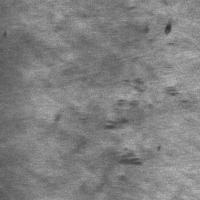

Supplement: S4 Dataset — The fourth typical strip steel surface defects dataset. (ZIP) [file pone.0212361.s004.zip › scale/RS_22.bmp]

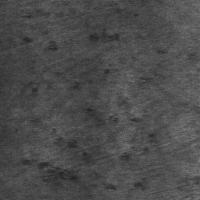

Supplement: S4 Dataset — The fourth typical strip steel surface defects dataset. (ZIP) [file pone.0212361.s004.zip › scale/RS_220.bmp]

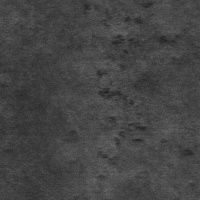

Supplement: S4 Dataset — The fourth typical strip steel surface defects dataset. (ZIP) [file pone.0212361.s004.zip › scale/RS_221.bmp]

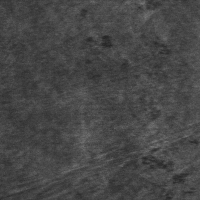

Supplement: S4 Dataset — The fourth typical strip steel surface defects dataset. (ZIP) [file pone.0212361.s004.zip › scale/RS_222.bmp]

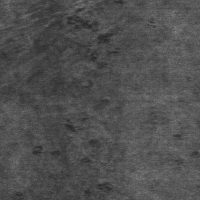

Supplement: S4 Dataset — The fourth typical strip steel surface defects dataset. (ZIP) [file pone.0212361.s004.zip › scale/RS_223.bmp]

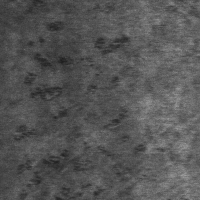

Supplement: S4 Dataset — The fourth typical strip steel surface defects dataset. (ZIP) [file pone.0212361.s004.zip › scale/RS_224.bmp]

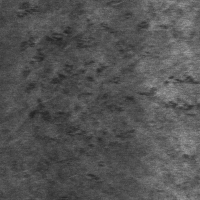

Supplement: S4 Dataset — The fourth typical strip steel surface defects dataset. (ZIP) [file pone.0212361.s004.zip › scale/RS_225.bmp]

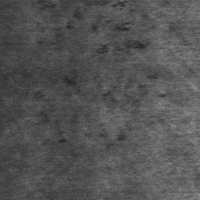

Supplement: S4 Dataset — The fourth typical strip steel surface defects dataset. (ZIP) [file pone.0212361.s004.zip › scale/RS_226.bmp]

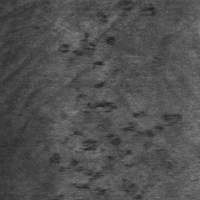

Supplement: S4 Dataset — The fourth typical strip steel surface defects dataset. (ZIP) [file pone.0212361.s004.zip › scale/RS_227.bmp]

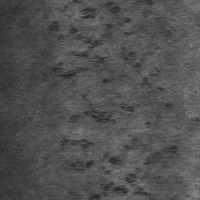

Supplement: S4 Dataset — The fourth typical strip steel surface defects dataset. (ZIP) [file pone.0212361.s004.zip › scale/RS_228.bmp]

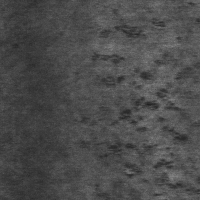

Supplement: S4 Dataset — The fourth typical strip steel surface defects dataset. (ZIP) [file pone.0212361.s004.zip › scale/RS_229.bmp]

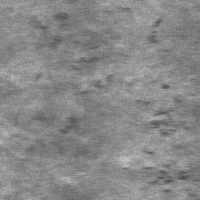

Supplement: S4 Dataset — The fourth typical strip steel surface defects dataset. (ZIP) [file pone.0212361.s004.zip › scale/RS_23.bmp]

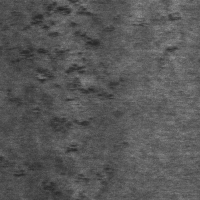

Supplement: S4 Dataset — The fourth typical strip steel surface defects dataset. (ZIP) [file pone.0212361.s004.zip › scale/RS_230.bmp]

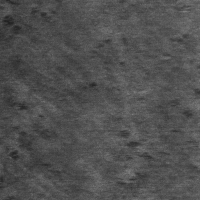

Supplement: S4 Dataset — The fourth typical strip steel surface defects dataset. (ZIP) [file pone.0212361.s004.zip › scale/RS_231.bmp]

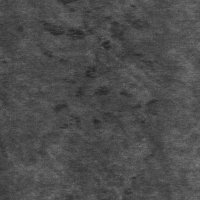

Supplement: S4 Dataset — The fourth typical strip steel surface defects dataset. (ZIP) [file pone.0212361.s004.zip › scale/RS_232.bmp]

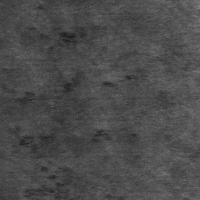

Supplement: S4 Dataset — The fourth typical strip steel surface defects dataset. (ZIP) [file pone.0212361.s004.zip › scale/RS_233.bmp]

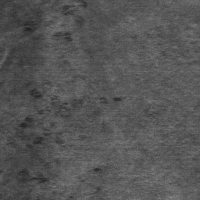

Supplement: S4 Dataset — The fourth typical strip steel surface defects dataset. (ZIP) [file pone.0212361.s004.zip › scale/RS_234.bmp]

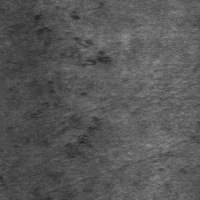

Supplement: S4 Dataset — The fourth typical strip steel surface defects dataset. (ZIP) [file pone.0212361.s004.zip › scale/RS_235.bmp]

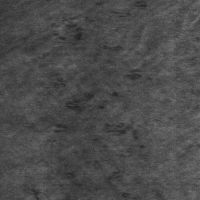

Supplement: S4 Dataset — The fourth typical strip steel surface defects dataset. (ZIP) [file pone.0212361.s004.zip › scale/RS_236.bmp]

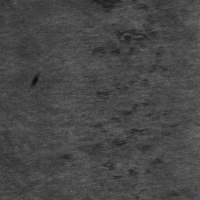

Supplement: S4 Dataset — The fourth typical strip steel surface defects dataset. (ZIP) [file pone.0212361.s004.zip › scale/RS_237.bmp]

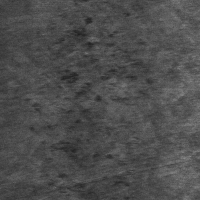

Supplement: S4 Dataset — The fourth typical strip steel surface defects dataset. (ZIP) [file pone.0212361.s004.zip › scale/RS_238.bmp]

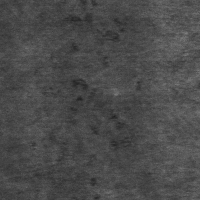

Supplement: S4 Dataset — The fourth typical strip steel surface defects dataset. (ZIP) [file pone.0212361.s004.zip › scale/RS_239.bmp]

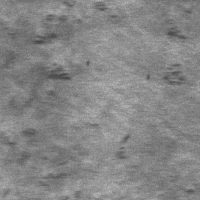

Supplement: S4 Dataset — The fourth typical strip steel surface defects dataset. (ZIP) [file pone.0212361.s004.zip › scale/RS_24.bmp]

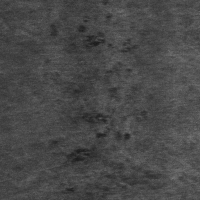

Supplement: S4 Dataset — The fourth typical strip steel surface defects dataset. (ZIP) [file pone.0212361.s004.zip › scale/RS_240.bmp]

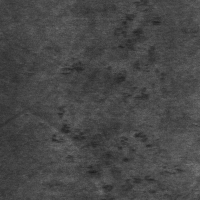

Supplement: S4 Dataset — The fourth typical strip steel surface defects dataset. (ZIP) [file pone.0212361.s004.zip › scale/RS_241.bmp]

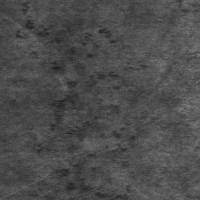

Supplement: S4 Dataset — The fourth typical strip steel surface defects dataset. (ZIP) [file pone.0212361.s004.zip › scale/RS_242.bmp]

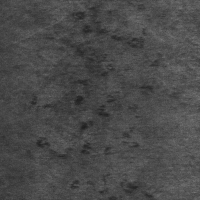

Supplement: S4 Dataset — The fourth typical strip steel surface defects dataset. (ZIP) [file pone.0212361.s004.zip › scale/RS_243.bmp]

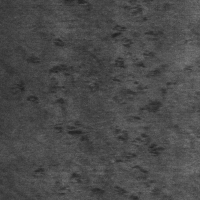

Supplement: S4 Dataset — The fourth typical strip steel surface defects dataset. (ZIP) [file pone.0212361.s004.zip › scale/RS_244.bmp]

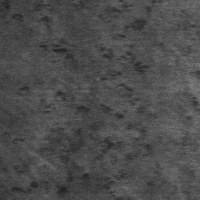

Supplement: S4 Dataset — The fourth typical strip steel surface defects dataset. (ZIP) [file pone.0212361.s004.zip › scale/RS_245.bmp]

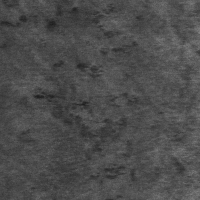

Supplement: S4 Dataset — The fourth typical strip steel surface defects dataset. (ZIP) [file pone.0212361.s004.zip › scale/RS_246.bmp]

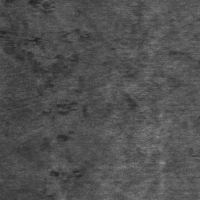

Supplement: S4 Dataset — The fourth typical strip steel surface defects dataset. (ZIP) [file pone.0212361.s004.zip › scale/RS_247.bmp]

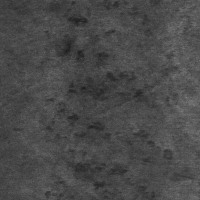

Supplement: S4 Dataset — The fourth typical strip steel surface defects dataset. (ZIP) [file pone.0212361.s004.zip › scale/RS_248.bmp]

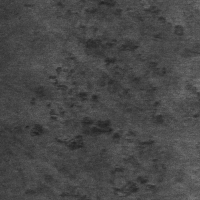

Supplement: S4 Dataset — The fourth typical strip steel surface defects dataset. (ZIP) [file pone.0212361.s004.zip › scale/RS_249.bmp]

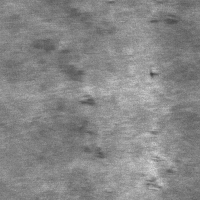

Supplement: S4 Dataset — The fourth typical strip steel surface defects dataset. (ZIP) [file pone.0212361.s004.zip › scale/RS_25.bmp]

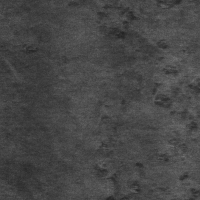

Supplement: S4 Dataset — The fourth typical strip steel surface defects dataset. (ZIP) [file pone.0212361.s004.zip › scale/RS_250.bmp]

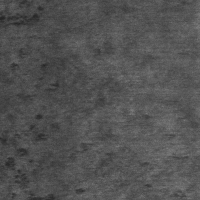

Supplement: S4 Dataset — The fourth typical strip steel surface defects dataset. (ZIP) [file pone.0212361.s004.zip › scale/RS_251.bmp]

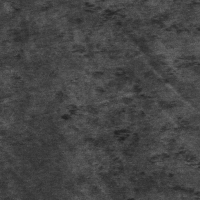

Supplement: S4 Dataset — The fourth typical strip steel surface defects dataset. (ZIP) [file pone.0212361.s004.zip › scale/RS_252.bmp]

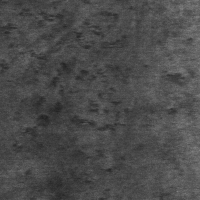

Supplement: S4 Dataset — The fourth typical strip steel surface defects dataset. (ZIP) [file pone.0212361.s004.zip › scale/RS_253.bmp]

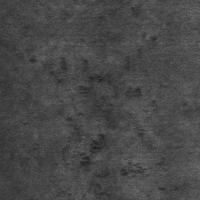

Supplement: S4 Dataset — The fourth typical strip steel surface defects dataset. (ZIP) [file pone.0212361.s004.zip › scale/RS_254.bmp]

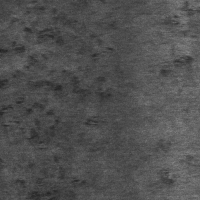

Supplement: S4 Dataset — The fourth typical strip steel surface defects dataset. (ZIP) [file pone.0212361.s004.zip › scale/RS_255.bmp]

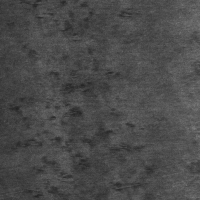

Supplement: S4 Dataset — The fourth typical strip steel surface defects dataset. (ZIP) [file pone.0212361.s004.zip › scale/RS_256.bmp]

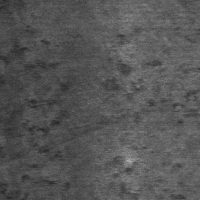

Supplement: S4 Dataset — The fourth typical strip steel surface defects dataset. (ZIP) [file pone.0212361.s004.zip › scale/RS_257.bmp]

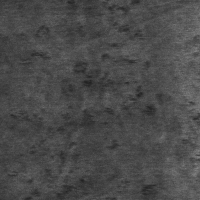

Supplement: S4 Dataset — The fourth typical strip steel surface defects dataset. (ZIP) [file pone.0212361.s004.zip › scale/RS_258.bmp]

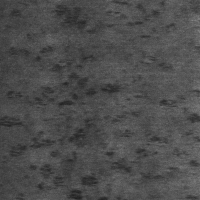

Supplement: S4 Dataset — The fourth typical strip steel surface defects dataset. (ZIP) [file pone.0212361.s004.zip › scale/RS_259.bmp]

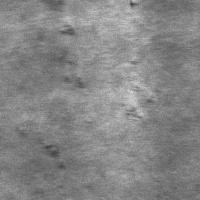

Supplement: S4 Dataset — The fourth typical strip steel surface defects dataset. (ZIP) [file pone.0212361.s004.zip › scale/RS_26.bmp]

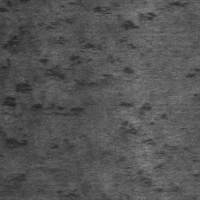

Supplement: S4 Dataset — The fourth typical strip steel surface defects dataset. (ZIP) [file pone.0212361.s004.zip › scale/RS_260.bmp]

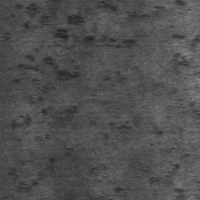

Supplement: S4 Dataset — The fourth typical strip steel surface defects dataset. (ZIP) [file pone.0212361.s004.zip › scale/RS_261.bmp]

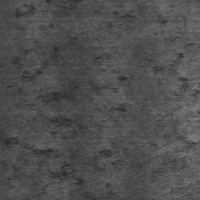

Supplement: S4 Dataset — The fourth typical strip steel surface defects dataset. (ZIP) [file pone.0212361.s004.zip › scale/RS_262.bmp]

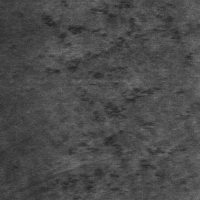

Supplement: S4 Dataset — The fourth typical strip steel surface defects dataset. (ZIP) [file pone.0212361.s004.zip › scale/RS_263.bmp]

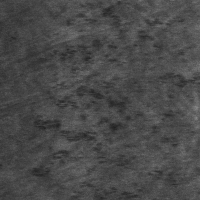

Supplement: S4 Dataset — The fourth typical strip steel surface defects dataset. (ZIP) [file pone.0212361.s004.zip › scale/RS_264.bmp]

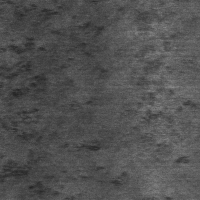

Supplement: S4 Dataset — The fourth typical strip steel surface defects dataset. (ZIP) [file pone.0212361.s004.zip › scale/RS_265.bmp]

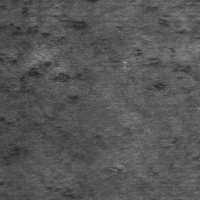

Supplement: S4 Dataset — The fourth typical strip steel surface defects dataset. (ZIP) [file pone.0212361.s004.zip › scale/RS_266.bmp]

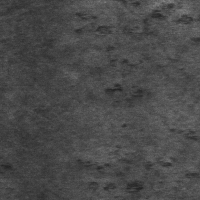

Supplement: S4 Dataset — The fourth typical strip steel surface defects dataset. (ZIP) [file pone.0212361.s004.zip › scale/RS_267.bmp]

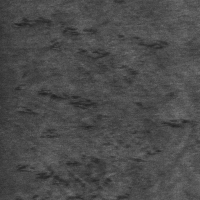

Supplement: S4 Dataset — The fourth typical strip steel surface defects dataset. (ZIP) [file pone.0212361.s004.zip › scale/RS_268.bmp]

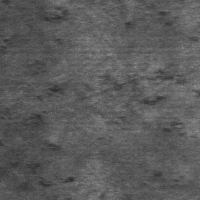

Supplement: S4 Dataset — The fourth typical strip steel surface defects dataset. (ZIP) [file pone.0212361.s004.zip › scale/RS_269.bmp]

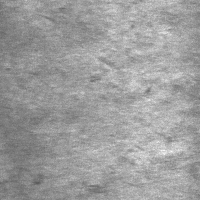

Supplement: S4 Dataset — The fourth typical strip steel surface defects dataset. (ZIP) [file pone.0212361.s004.zip › scale/RS_27.bmp]

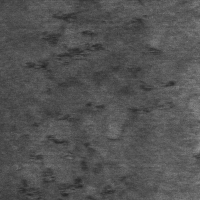

Supplement: S4 Dataset — The fourth typical strip steel surface defects dataset. (ZIP) [file pone.0212361.s004.zip › scale/RS_270.bmp]

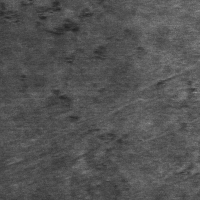

Supplement: S4 Dataset — The fourth typical strip steel surface defects dataset. (ZIP) [file pone.0212361.s004.zip › scale/RS_271.bmp]

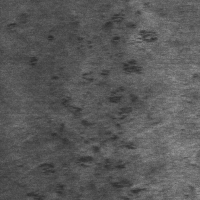

Supplement: S4 Dataset — The fourth typical strip steel surface defects dataset. (ZIP) [file pone.0212361.s004.zip › scale/RS_272.bmp]

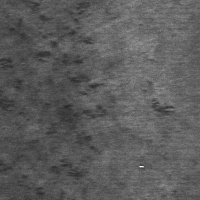

Supplement: S4 Dataset — The fourth typical strip steel surface defects dataset. (ZIP) [file pone.0212361.s004.zip › scale/RS_273.bmp]

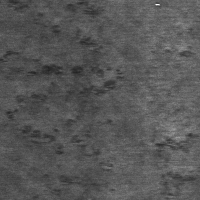

Supplement: S4 Dataset — The fourth typical strip steel surface defects dataset. (ZIP) [file pone.0212361.s004.zip › scale/RS_274.bmp]

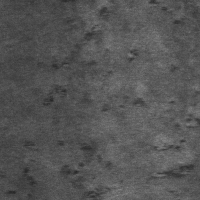

Supplement: S4 Dataset — The fourth typical strip steel surface defects dataset. (ZIP) [file pone.0212361.s004.zip › scale/RS_275.bmp]

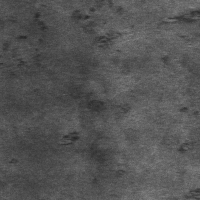

Supplement: S4 Dataset — The fourth typical strip steel surface defects dataset. (ZIP) [file pone.0212361.s004.zip › scale/RS_276.bmp]

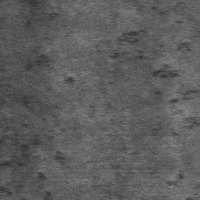

Supplement: S4 Dataset — The fourth typical strip steel surface defects dataset. (ZIP) [file pone.0212361.s004.zip › scale/RS_277.bmp]

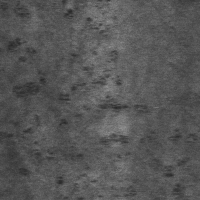

Supplement: S4 Dataset — The fourth typical strip steel surface defects dataset. (ZIP) [file pone.0212361.s004.zip › scale/RS_278.bmp]

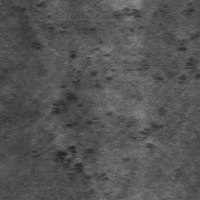

Supplement: S4 Dataset — The fourth typical strip steel surface defects dataset. (ZIP) [file pone.0212361.s004.zip › scale/RS_279.bmp]
