# Supplementary figures and images for: Support vector machine with quantile hyper-spheres for pattern classification (part 5 of 6)
Source: PLoS One. 2019 Feb 15;14(2):e0212361. doi: 10.1371/journal.pone.0212361 (PMC6377146; doi:10.1371/journal.pone.0212361)

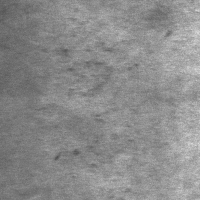

Supplement: S4 Dataset — The fourth typical strip steel surface defects dataset. (ZIP) [file pone.0212361.s004.zip › scale/RS_1.bmp]

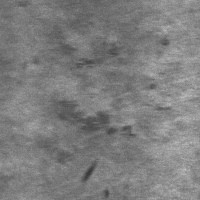

Supplement: S4 Dataset — The fourth typical strip steel surface defects dataset. (ZIP) [file pone.0212361.s004.zip › scale/RS_10.bmp]

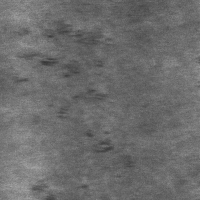

Supplement: S4 Dataset — The fourth typical strip steel surface defects dataset. (ZIP) [file pone.0212361.s004.zip › scale/RS_100.bmp]

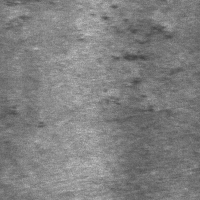

Supplement: S4 Dataset — The fourth typical strip steel surface defects dataset. (ZIP) [file pone.0212361.s004.zip › scale/RS_101.bmp]

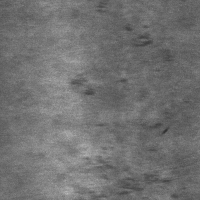

Supplement: S4 Dataset — The fourth typical strip steel surface defects dataset. (ZIP) [file pone.0212361.s004.zip › scale/RS_102.bmp]

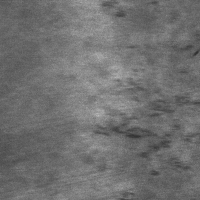

Supplement: S4 Dataset — The fourth typical strip steel surface defects dataset. (ZIP) [file pone.0212361.s004.zip › scale/RS_103.bmp]

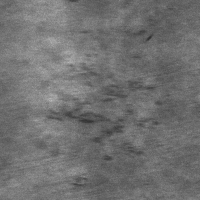

Supplement: S4 Dataset — The fourth typical strip steel surface defects dataset. (ZIP) [file pone.0212361.s004.zip › scale/RS_104.bmp]

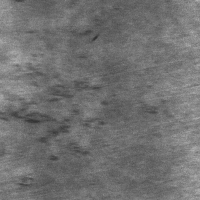

Supplement: S4 Dataset — The fourth typical strip steel surface defects dataset. (ZIP) [file pone.0212361.s004.zip › scale/RS_105.bmp]

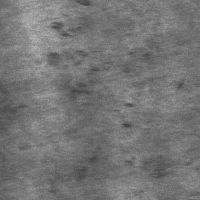

Supplement: S4 Dataset — The fourth typical strip steel surface defects dataset. (ZIP) [file pone.0212361.s004.zip › scale/RS_106.bmp]

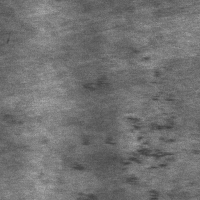

Supplement: S4 Dataset — The fourth typical strip steel surface defects dataset. (ZIP) [file pone.0212361.s004.zip › scale/RS_107.bmp]

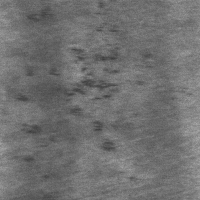

Supplement: S4 Dataset — The fourth typical strip steel surface defects dataset. (ZIP) [file pone.0212361.s004.zip › scale/RS_108.bmp]

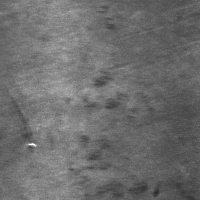

Supplement: S4 Dataset — The fourth typical strip steel surface defects dataset. (ZIP) [file pone.0212361.s004.zip › scale/RS_109.bmp]

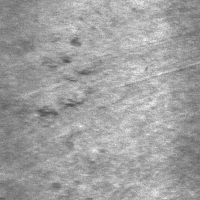

Supplement: S4 Dataset — The fourth typical strip steel surface defects dataset. (ZIP) [file pone.0212361.s004.zip › scale/RS_11.bmp]

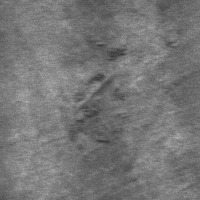

Supplement: S4 Dataset — The fourth typical strip steel surface defects dataset. (ZIP) [file pone.0212361.s004.zip › scale/RS_110.bmp]

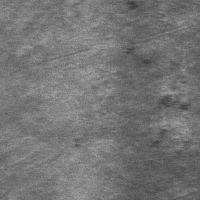

Supplement: S4 Dataset — The fourth typical strip steel surface defects dataset. (ZIP) [file pone.0212361.s004.zip › scale/RS_111.bmp]

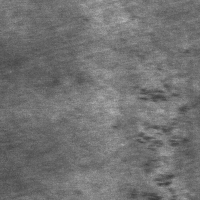

Supplement: S4 Dataset — The fourth typical strip steel surface defects dataset. (ZIP) [file pone.0212361.s004.zip › scale/RS_112.bmp]

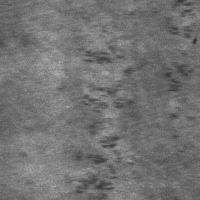

Supplement: S4 Dataset — The fourth typical strip steel surface defects dataset. (ZIP) [file pone.0212361.s004.zip › scale/RS_113.bmp]

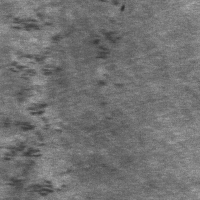

Supplement: S4 Dataset — The fourth typical strip steel surface defects dataset. (ZIP) [file pone.0212361.s004.zip › scale/RS_114.bmp]

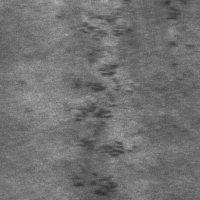

Supplement: S4 Dataset — The fourth typical strip steel surface defects dataset. (ZIP) [file pone.0212361.s004.zip › scale/RS_115.bmp]

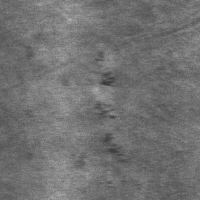

Supplement: S4 Dataset — The fourth typical strip steel surface defects dataset. (ZIP) [file pone.0212361.s004.zip › scale/RS_116.bmp]

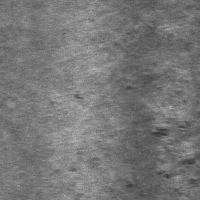

Supplement: S4 Dataset — The fourth typical strip steel surface defects dataset. (ZIP) [file pone.0212361.s004.zip › scale/RS_117.bmp]

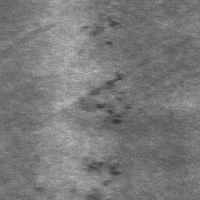

Supplement: S4 Dataset — The fourth typical strip steel surface defects dataset. (ZIP) [file pone.0212361.s004.zip › scale/RS_118.bmp]

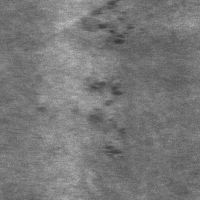

Supplement: S4 Dataset — The fourth typical strip steel surface defects dataset. (ZIP) [file pone.0212361.s004.zip › scale/RS_119.bmp]

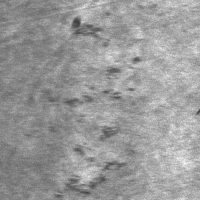

Supplement: S4 Dataset — The fourth typical strip steel surface defects dataset. (ZIP) [file pone.0212361.s004.zip › scale/RS_12.bmp]

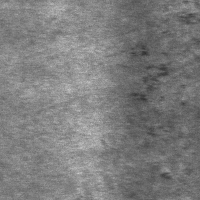

Supplement: S4 Dataset — The fourth typical strip steel surface defects dataset. (ZIP) [file pone.0212361.s004.zip › scale/RS_120.bmp]

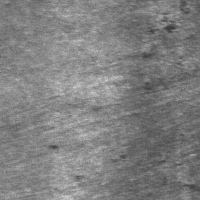

Supplement: S4 Dataset — The fourth typical strip steel surface defects dataset. (ZIP) [file pone.0212361.s004.zip › scale/RS_121.bmp]

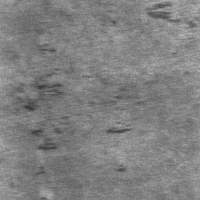

Supplement: S4 Dataset — The fourth typical strip steel surface defects dataset. (ZIP) [file pone.0212361.s004.zip › scale/RS_122.bmp]

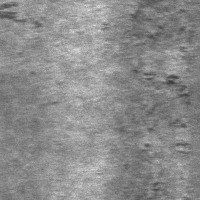

Supplement: S4 Dataset — The fourth typical strip steel surface defects dataset. (ZIP) [file pone.0212361.s004.zip › scale/RS_123.bmp]

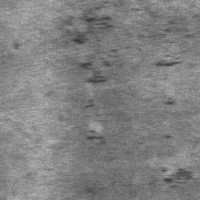

Supplement: S4 Dataset — The fourth typical strip steel surface defects dataset. (ZIP) [file pone.0212361.s004.zip › scale/RS_124.bmp]

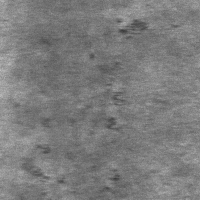

Supplement: S4 Dataset — The fourth typical strip steel surface defects dataset. (ZIP) [file pone.0212361.s004.zip › scale/RS_125.bmp]

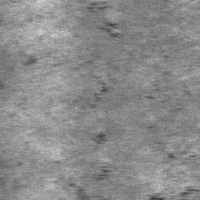

Supplement: S4 Dataset — The fourth typical strip steel surface defects dataset. (ZIP) [file pone.0212361.s004.zip › scale/RS_126.bmp]

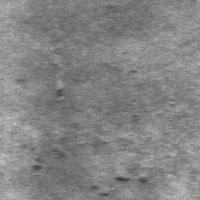

Supplement: S4 Dataset — The fourth typical strip steel surface defects dataset. (ZIP) [file pone.0212361.s004.zip › scale/RS_127.bmp]

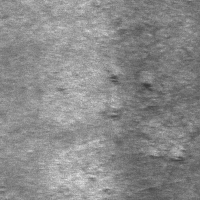

Supplement: S4 Dataset — The fourth typical strip steel surface defects dataset. (ZIP) [file pone.0212361.s004.zip › scale/RS_128.bmp]

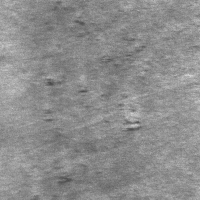

Supplement: S4 Dataset — The fourth typical strip steel surface defects dataset. (ZIP) [file pone.0212361.s004.zip › scale/RS_129.bmp]

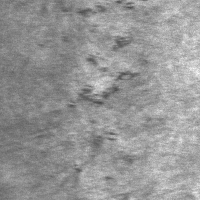

Supplement: S4 Dataset — The fourth typical strip steel surface defects dataset. (ZIP) [file pone.0212361.s004.zip › scale/RS_13.bmp]

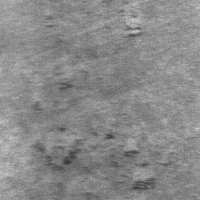

Supplement: S4 Dataset — The fourth typical strip steel surface defects dataset. (ZIP) [file pone.0212361.s004.zip › scale/RS_130.bmp]

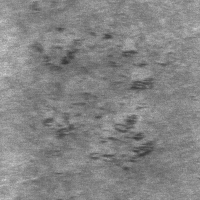

Supplement: S4 Dataset — The fourth typical strip steel surface defects dataset. (ZIP) [file pone.0212361.s004.zip › scale/RS_131.bmp]

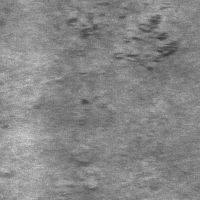

Supplement: S4 Dataset — The fourth typical strip steel surface defects dataset. (ZIP) [file pone.0212361.s004.zip › scale/RS_132.bmp]

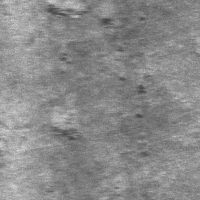

Supplement: S4 Dataset — The fourth typical strip steel surface defects dataset. (ZIP) [file pone.0212361.s004.zip › scale/RS_133.bmp]

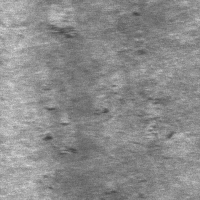

Supplement: S4 Dataset — The fourth typical strip steel surface defects dataset. (ZIP) [file pone.0212361.s004.zip › scale/RS_134.bmp]

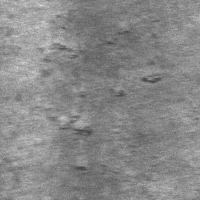

Supplement: S4 Dataset — The fourth typical strip steel surface defects dataset. (ZIP) [file pone.0212361.s004.zip › scale/RS_135.bmp]

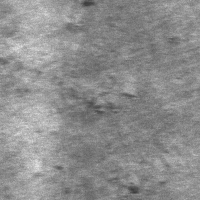

Supplement: S4 Dataset — The fourth typical strip steel surface defects dataset. (ZIP) [file pone.0212361.s004.zip › scale/RS_136.bmp]

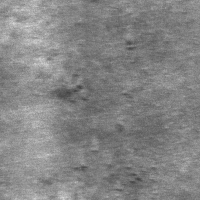

Supplement: S4 Dataset — The fourth typical strip steel surface defects dataset. (ZIP) [file pone.0212361.s004.zip › scale/RS_137.bmp]

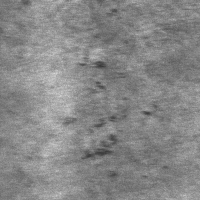

Supplement: S4 Dataset — The fourth typical strip steel surface defects dataset. (ZIP) [file pone.0212361.s004.zip › scale/RS_138.bmp]

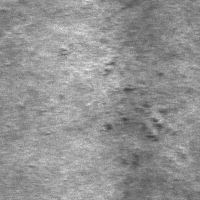

Supplement: S4 Dataset — The fourth typical strip steel surface defects dataset. (ZIP) [file pone.0212361.s004.zip › scale/RS_139.bmp]

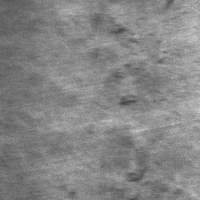

Supplement: S4 Dataset — The fourth typical strip steel surface defects dataset. (ZIP) [file pone.0212361.s004.zip › scale/RS_14.bmp]

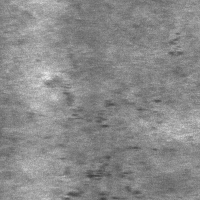

Supplement: S4 Dataset — The fourth typical strip steel surface defects dataset. (ZIP) [file pone.0212361.s004.zip › scale/RS_140.bmp]

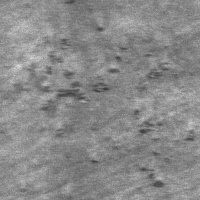

Supplement: S4 Dataset — The fourth typical strip steel surface defects dataset. (ZIP) [file pone.0212361.s004.zip › scale/RS_141.bmp]

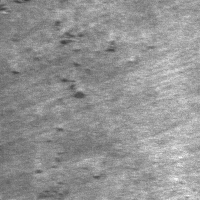

Supplement: S4 Dataset — The fourth typical strip steel surface defects dataset. (ZIP) [file pone.0212361.s004.zip › scale/RS_142.bmp]

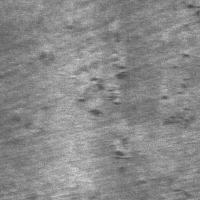

Supplement: S4 Dataset — The fourth typical strip steel surface defects dataset. (ZIP) [file pone.0212361.s004.zip › scale/RS_143.bmp]

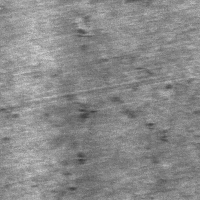

Supplement: S4 Dataset — The fourth typical strip steel surface defects dataset. (ZIP) [file pone.0212361.s004.zip › scale/RS_144.bmp]

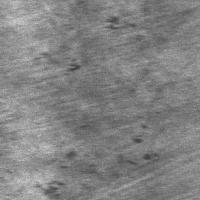

Supplement: S4 Dataset — The fourth typical strip steel surface defects dataset. (ZIP) [file pone.0212361.s004.zip › scale/RS_145.bmp]

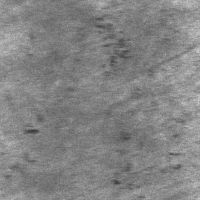

Supplement: S4 Dataset — The fourth typical strip steel surface defects dataset. (ZIP) [file pone.0212361.s004.zip › scale/RS_146.bmp]

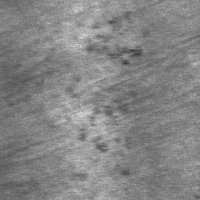

Supplement: S4 Dataset — The fourth typical strip steel surface defects dataset. (ZIP) [file pone.0212361.s004.zip › scale/RS_147.bmp]

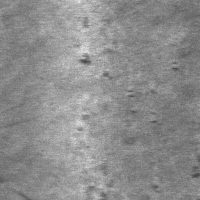

Supplement: S4 Dataset — The fourth typical strip steel surface defects dataset. (ZIP) [file pone.0212361.s004.zip › scale/RS_148.bmp]

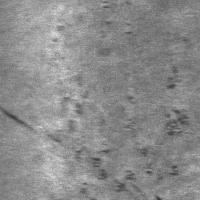

Supplement: S4 Dataset — The fourth typical strip steel surface defects dataset. (ZIP) [file pone.0212361.s004.zip › scale/RS_149.bmp]

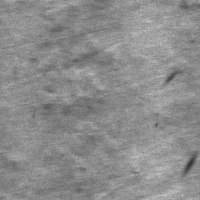

Supplement: S4 Dataset — The fourth typical strip steel surface defects dataset. (ZIP) [file pone.0212361.s004.zip › scale/RS_15.bmp]

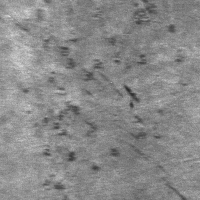

Supplement: S4 Dataset — The fourth typical strip steel surface defects dataset. (ZIP) [file pone.0212361.s004.zip › scale/RS_150.bmp]

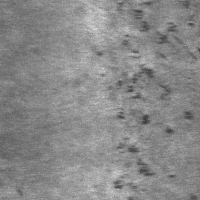

Supplement: S4 Dataset — The fourth typical strip steel surface defects dataset. (ZIP) [file pone.0212361.s004.zip › scale/RS_151.bmp]

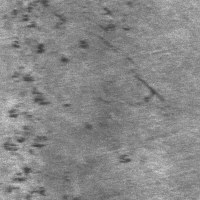

Supplement: S4 Dataset — The fourth typical strip steel surface defects dataset. (ZIP) [file pone.0212361.s004.zip › scale/RS_152.bmp]

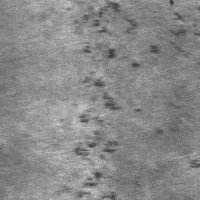

Supplement: S4 Dataset — The fourth typical strip steel surface defects dataset. (ZIP) [file pone.0212361.s004.zip › scale/RS_153.bmp]

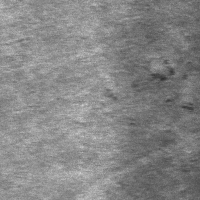

Supplement: S4 Dataset — The fourth typical strip steel surface defects dataset. (ZIP) [file pone.0212361.s004.zip › scale/RS_154.bmp]

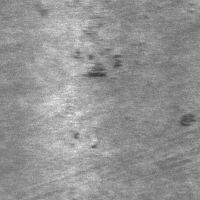

Supplement: S4 Dataset — The fourth typical strip steel surface defects dataset. (ZIP) [file pone.0212361.s004.zip › scale/RS_155.bmp]

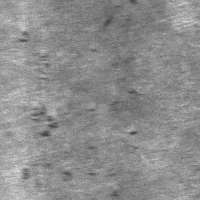

Supplement: S4 Dataset — The fourth typical strip steel surface defects dataset. (ZIP) [file pone.0212361.s004.zip › scale/RS_156.bmp]

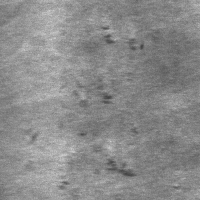

Supplement: S4 Dataset — The fourth typical strip steel surface defects dataset. (ZIP) [file pone.0212361.s004.zip › scale/RS_157.bmp]

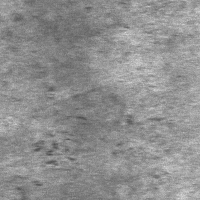

Supplement: S4 Dataset — The fourth typical strip steel surface defects dataset. (ZIP) [file pone.0212361.s004.zip › scale/RS_158.bmp]

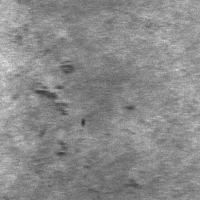

Supplement: S4 Dataset — The fourth typical strip steel surface defects dataset. (ZIP) [file pone.0212361.s004.zip › scale/RS_159.bmp]

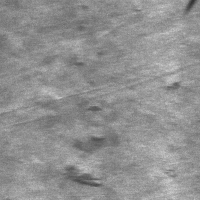

Supplement: S4 Dataset — The fourth typical strip steel surface defects dataset. (ZIP) [file pone.0212361.s004.zip › scale/RS_16.bmp]

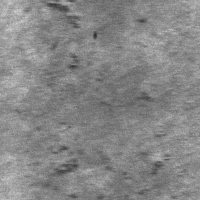

Supplement: S4 Dataset — The fourth typical strip steel surface defects dataset. (ZIP) [file pone.0212361.s004.zip › scale/RS_160.bmp]

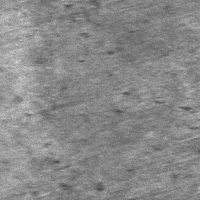

Supplement: S4 Dataset — The fourth typical strip steel surface defects dataset. (ZIP) [file pone.0212361.s004.zip › scale/RS_161.bmp]

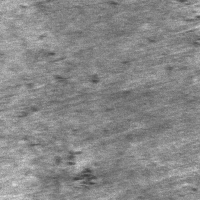

Supplement: S4 Dataset — The fourth typical strip steel surface defects dataset. (ZIP) [file pone.0212361.s004.zip › scale/RS_162.bmp]

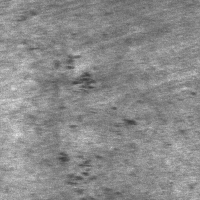

Supplement: S4 Dataset — The fourth typical strip steel surface defects dataset. (ZIP) [file pone.0212361.s004.zip › scale/RS_163.bmp]

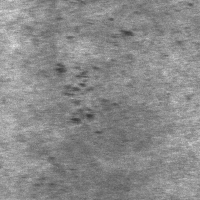

Supplement: S4 Dataset — The fourth typical strip steel surface defects dataset. (ZIP) [file pone.0212361.s004.zip › scale/RS_164.bmp]

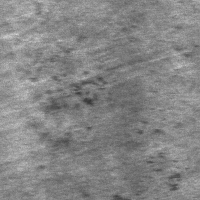

Supplement: S4 Dataset — The fourth typical strip steel surface defects dataset. (ZIP) [file pone.0212361.s004.zip › scale/RS_165.bmp]

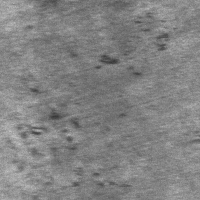

Supplement: S4 Dataset — The fourth typical strip steel surface defects dataset. (ZIP) [file pone.0212361.s004.zip › scale/RS_166.bmp]

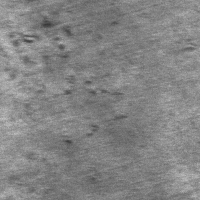

Supplement: S4 Dataset — The fourth typical strip steel surface defects dataset. (ZIP) [file pone.0212361.s004.zip › scale/RS_167.bmp]

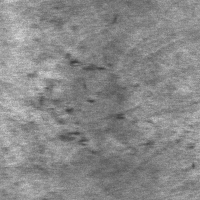

Supplement: S4 Dataset — The fourth typical strip steel surface defects dataset. (ZIP) [file pone.0212361.s004.zip › scale/RS_168.bmp]

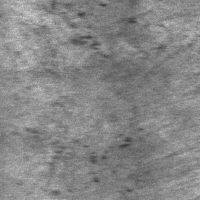

Supplement: S4 Dataset — The fourth typical strip steel surface defects dataset. (ZIP) [file pone.0212361.s004.zip › scale/RS_169.bmp]

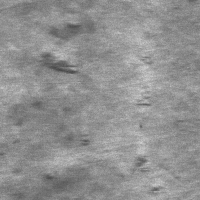

Supplement: S4 Dataset — The fourth typical strip steel surface defects dataset. (ZIP) [file pone.0212361.s004.zip › scale/RS_17.bmp]

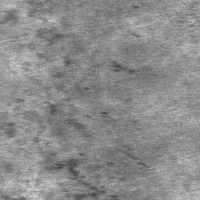

Supplement: S4 Dataset — The fourth typical strip steel surface defects dataset. (ZIP) [file pone.0212361.s004.zip › scale/RS_170.bmp]

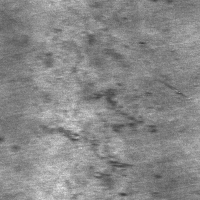

Supplement: S4 Dataset — The fourth typical strip steel surface defects dataset. (ZIP) [file pone.0212361.s004.zip › scale/RS_171.bmp]

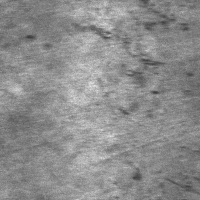

Supplement: S4 Dataset — The fourth typical strip steel surface defects dataset. (ZIP) [file pone.0212361.s004.zip › scale/RS_172.bmp]

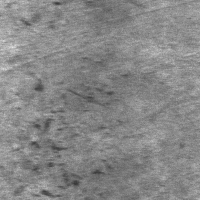

Supplement: S4 Dataset — The fourth typical strip steel surface defects dataset. (ZIP) [file pone.0212361.s004.zip › scale/RS_173.bmp]

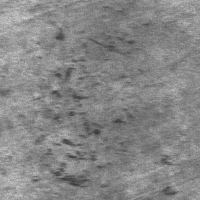

Supplement: S4 Dataset — The fourth typical strip steel surface defects dataset. (ZIP) [file pone.0212361.s004.zip › scale/RS_174.bmp]

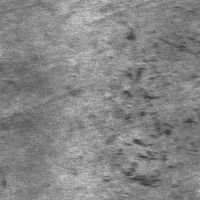

Supplement: S4 Dataset — The fourth typical strip steel surface defects dataset. (ZIP) [file pone.0212361.s004.zip › scale/RS_175.bmp]

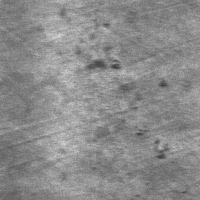

Supplement: S4 Dataset — The fourth typical strip steel surface defects dataset. (ZIP) [file pone.0212361.s004.zip › scale/RS_176.bmp]

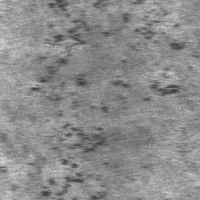

Supplement: S4 Dataset — The fourth typical strip steel surface defects dataset. (ZIP) [file pone.0212361.s004.zip › scale/RS_177.bmp]

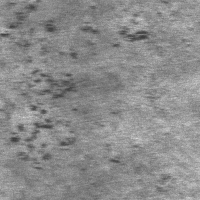

Supplement: S4 Dataset — The fourth typical strip steel surface defects dataset. (ZIP) [file pone.0212361.s004.zip › scale/RS_178.bmp]

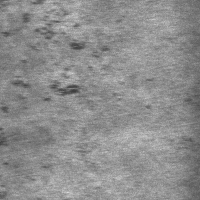

Supplement: S4 Dataset — The fourth typical strip steel surface defects dataset. (ZIP) [file pone.0212361.s004.zip › scale/RS_179.bmp]

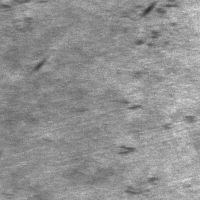

Supplement: S4 Dataset — The fourth typical strip steel surface defects dataset. (ZIP) [file pone.0212361.s004.zip › scale/RS_18.bmp]

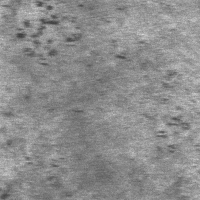

Supplement: S4 Dataset — The fourth typical strip steel surface defects dataset. (ZIP) [file pone.0212361.s004.zip › scale/RS_180.bmp]

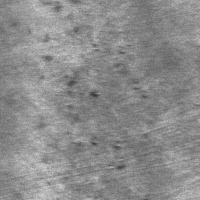

Supplement: S4 Dataset — The fourth typical strip steel surface defects dataset. (ZIP) [file pone.0212361.s004.zip › scale/RS_181.bmp]

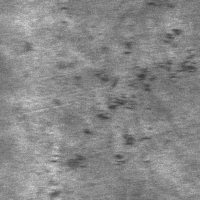

Supplement: S4 Dataset — The fourth typical strip steel surface defects dataset. (ZIP) [file pone.0212361.s004.zip › scale/RS_182.bmp]

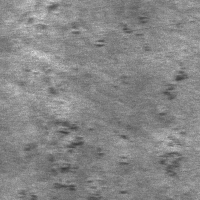

Supplement: S4 Dataset — The fourth typical strip steel surface defects dataset. (ZIP) [file pone.0212361.s004.zip › scale/RS_183.bmp]

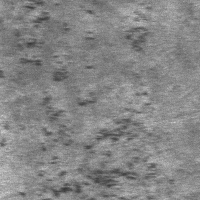

Supplement: S4 Dataset — The fourth typical strip steel surface defects dataset. (ZIP) [file pone.0212361.s004.zip › scale/RS_184.bmp]

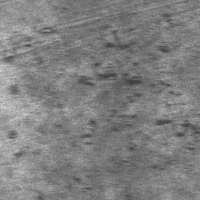

Supplement: S4 Dataset — The fourth typical strip steel surface defects dataset. (ZIP) [file pone.0212361.s004.zip › scale/RS_185.bmp]

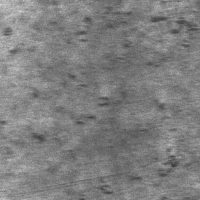

Supplement: S4 Dataset — The fourth typical strip steel surface defects dataset. (ZIP) [file pone.0212361.s004.zip › scale/RS_186.bmp]

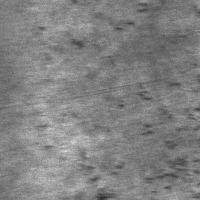

Supplement: S4 Dataset — The fourth typical strip steel surface defects dataset. (ZIP) [file pone.0212361.s004.zip › scale/RS_187.bmp]

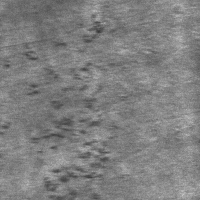

Supplement: S4 Dataset — The fourth typical strip steel surface defects dataset. (ZIP) [file pone.0212361.s004.zip › scale/RS_188.bmp]

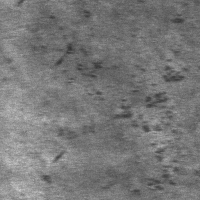

Supplement: S4 Dataset — The fourth typical strip steel surface defects dataset. (ZIP) [file pone.0212361.s004.zip › scale/RS_189.bmp]
